# First Insight into Microbiome Profiles of Myrmecophilous Beetles and Their Host, Red Wood Ant *Formica polyctena* (Hymenoptera: Formicidae)—A Case Study

**DOI:** 10.3390/insects11020134

**Published:** 2020-02-19

**Authors:** Agnieszka Kaczmarczyk-Ziemba, Mirosław Zagaja, Grzegorz K. Wagner, Ewa Pietrykowska-Tudruj, Bernard Staniec

**Affiliations:** 1 Department of Genetics and Biosystematics, Faculty of Biology, University of Gdansk, Wita Stwosza 59, 80-308 Gdansk, Poland; 2 Isobolographic Analysis Laboratory, Institute of Rural Health, Jaczewskiego 2, 20-090 Lublin, Poland; zagaja.miroslaw@imw.lublin.pl; 3 Department of Zoology and Nature Protection, Maria Curie-Sklodowska University, Akademicka 19, 20-033 Lublin, Poland; grzegorz.wagner@poczta.umcs.lublin.pl (G.K.W.); ewa.pietrykowska-tudruj@poczta.umcs.lublin.pl (E.P.-T.); bernard.staniec@poczta.umcs.lublin.pl (B.S.)

**Keywords:** 16S rRNA, *Formica polyctena*, microbiome, myrmecophile, *Rickettsia*, *Wolbachia*

## Abstract

*Formica polyctena* belongs to the red wood ant species group. Its nests provide a stable, food rich, and temperature and humidity controlled environment, utilized by a wide range of species, called myrmecophiles. Here, we used the high-throughput sequencing of the 16S rRNA gene on the Illumina platform for identification of the microbiome profiles of six selected myrmecophilous beetles (*Dendrophilus pygmaeus*, *Leptacinus formicetorum*, *Monotoma angusticollis*, *Myrmechixenus subterraneus*, *Ptenidium formicetorum* and *Thiasophila angulata*) and their host *F. polyctena*. Analyzed bacterial communities consisted of a total of 23 phyla, among which Proteobacteria, Actinobacteria, and Firmicutes were the most abundant. Two known endosymbionts—*Wolbachia* and *Rickettsia*—were found in the analyzed microbiome profiles and *Wolbachia* was dominant in bacterial communities associated with *F. polyctena*, *M. subterraneus*, *L. formicetorum* and *P. formicetorum* (>90% of reads). In turn, *M. angusticollis* was co-infected with both *Wolbachia* and *Rickettsia*, while in the microbiome of *T. angulata,* the dominance of *Rickettsia* has been observed. The relationships among the microbiome profiles were complex, and no relative abundance pattern common to all myrmecophilous beetles tested was observed. However, some subtle, species-specific patterns have been observed for bacterial communities associated with *D. pygmaeus*, *M. angusticollis*, and *T. angulata.*

## 1. Introduction

Myrmecophiles (or ant associates) are organisms that are dependent on various ant species, at least during part of their lifecycle. They are able to penetrate and survive inside the heavily defended ants’ nests [1,2,3]. A very large number of myrmecophiles are insects, mainly beetles [3,4]. Besides them, there are myrmecophiles, among other, often distant, systematic groups, including flies, crickets, butterflies, bristletails, millipedes, isopods, mites, spiders, aphids, scale insects, wasps, and even snails and snakes [1]. Myrmecophiles include both the most morphologically and behaviorally specialized animals as well as poorly specialized ones. As a consequence of interactions with ants, they adopted a variety of unique features to evade or avoid ant attacks [1,5]. Therefore, myrmecophiles are very attractive research subjects in studies on their systematic differentiation, ecology, life history, and phylogeny [6,7,8,9,10].

Despite the great interest in biology and ecology of myrmecophiles, studies on the microorganisms associated with them, the potential similarities of microbiome profiles of ants and their associates, and the probable horizontal transfer of microorganisms among insects inhabiting or visiting ants nests are limited. A recent study focusing on the microbiota of several honeydew-producing *Prociphilus* aphids and *Rhizoecus* mealybugs farming by *Brachymyrmex* and *Lasius* ants revealed a phylogenetic signal in the microbiomes of trophobionts [11]. Each selected species, despite being farmed by the same ants, harbored specific strains of identified endosymbionts (*Buchnera*, *Tremblaya*, *Sodalis*, and *Serratia*). That result was explained by the strict vertical transmission of endosymbionts between generations of trophobionts. In contrast, tested *Brachymyrmex* and *Lasius* microbiome was possibly shaped by host’s partnerships, and ant-bacteria associations were subject to horizontal transmission or social transmission within colonies [11]. Bacterial communities were also profiled for socially parasitic *Megalomyrmex* ants and their fungus-growing ant hosts from the genera *Cyphomyrmex*, *Trachymyrmex,* and *Sericomyrmex* [12]. Performed analyses showed that social parasites and their hosts shared a subset of bacterial symbionts (Entomoplasmatales, Bartonellaceae, *Acinetobacter*, *Wolbachia*, and *Pseudonocardia*) and that Entomoplasmatales and Bartonellaceae can co-infect specifically associated combinations of hosts and social parasites with identical 16S rRNA genotypes. Moreover, obtained results implied that cohabiting ant social parasites and hosts may obtain functional benefits from bacterial symbiont transfer even when they are not closely related.

Although studies by Ivens et al. (2018) [11] and Liberti et al. (2015) [12] shed light on the microbiome profiles of some ants associates, there are still a lot of ambiguities. Because of hidden life, the distribution and abundance of myrmecophiles are unclear and likely underestimated. Nevertheless, it is thought that beetles consist of the richest and the most diverse group among ant associates [3]. Although the exact numbers of myrmecophilous beetles associated with particular ants are usually barely estimated, in some cases (e.g., for ants representing *Formica* genus), this group has been better recognized.

The ants of the genus *Formica* are the widespread and significant component of forest ecosystems. They influence soil qualities and the presence of some plant species. *Formica* ants also have a strong influence on surrounding zoocenosis [13]. Within the *Formica* genus, one may distinguish the *Formica rufa* group (commonly called *red wood ants*). All red wood ant’s species are morphologically very similar and show high intraspecific variability [14]. Therefore, they belong to the most studied ant species in Europe with respect to their biology, ecology, and phylogeny [14,15,16,17,18]. Furthermore, these ants build complex nests with a stable level of temperature and humidity, as well as a constant and diversified food resource, which are the optimal place to live for a great number of myrmecophilous insects. For example, as many as 56 myrmecophilous beetle species have been found in the nests of representatives of the genus *Formica* [7]. Bacterial communities associated with these species have not been investigated exhaustively, and the tri-party relationships occurring among host ant, myrmecophilous beetle, and associated bacterial communities have not been investigated yet for *Formica* representatives.

In the present study, our main goal was to shed light on complex tripartite relationships among *F. polyctena*, selected myrmecophilous beetles commonly inhabiting its nest and microbial communities associated with all species tested. We selected six beetle species belonging to five different families: *Thiasophila angulata* (Erichson, 1837), *Leptacinus formicetorum* (Märkel, 1841) (Staphylinidae), *Dendrophilus pygmaeus* (Linnaeus, 1758) (Histeridae), *Monotoma angusticollis* (Gyllenhal, 1877) (Monotomidae), *Ptenidium formicetorum* (Kraatz, 1851) (Ptiliidae) and *Myrmechixenus subterraneus* (Chevrolat, 1835) (Tenebrionidae). All selected species are commonly found inside *F. polyctena* nests, but they are rather unspecialized in the level of integration into colony [19,20]. Two representatives of Staphylinidae: *Thiasophila angulata* and *Leptacinus formicetorum* are predators, actively seeking out early developmental stages of ants (eggs and larvae) and other invertebrates inhabiting nests of red wood ants. They can also scavenge dead bodies of adult ants and other insects in the anthill [2,21]. What is more, *T. angulate* adapts its behavior to avoid ants aggression or to defend when attacked [21,22]. *Dendrophilus pygmaeus* (Histeridae) is carnivorous, feeding on insect remains or dead ants. It is classified as a synechthran. Convex shape and hard exoskeleton, as well as the ability to conceal its antennae and legs (e.g., *fovea antennalis* as a morphological adaptation), allow them to avoid various ant attacks [23]. *Monotoma angusticollis* (Monotomidae) is a typical scavenger that feeds on animal food, such as dead ants or ants’ prey [2]. This myrmecophile is a small, slow-moving beetle, which retracts its legs when attacked, making it difficult to detect [9]. *Ptenidium formicetorum* (Ptiliidae ) is a very small beetle, which feeds on animal food and probably hunts small mites or springtails, commonly occurring in the nests of red wood ants. The last studies species, *Myrmechixenus subterraneus* (Tenebrionidae), is a well-known Euro-Siberian species, common in the nests of *Formica* ant species. It is also included in the synechthran category [24].

The presented study aimed to define the microbiome profiles of selected myrmecophiles and their host *F. polyctena* using the Next Generation Sequencing of the 16S rRNA gene. The bacterial communities were screened to establish the presence of known endosymbionts and bacterial groups that may be related to the adaptations to the conditions prevailing in the nest.

## 2. Materials and Methods 

### 2.1. Sample Collection

Samples were collected as part of a research project on the fauna of myrmecophilous beetles inhabiting nests of the red wood ant *F. polyctena* in the Polesie National Park (Poland). The permission for field studies has been granted by the Ministry of the Environment, Poland (permission nos. DLP-III.286.148.2016.MGr and DLP-III.286.141.2016.MGr).

Specimens were collected during the summer and autumn of 2017. *Monotoma angusticollis* (n = 5), *Ptenidium formicetorum* (n = 17), *Leptacinus formicetorum* (n = 4), and *F. polyctena* (n = 20) were collected from the nest near the Dlugie Lake (N 51°27′04.0″ E 23°09′39.9″). *Myrmechixenus subterraneus* (n = 15), *Thiasophila angulate* (n = 6), *Dendrophilus pygmaeus* (n = 3) as well as the second sample of *F. polyctena* (n = 20) were collected from a nest near the Lipniak village (N 51°27′51.2″ E 23°06′53.5″).

Approximately three liters of the nest material was taken from a depth of 20–30 cm and then densified using a standard entomological sieve. The obtained nest material, along with the live insects found, was placed in cloth pouches and transferred to the laboratory. Species were identified by the authors, according to particular insect identification keys [25,26,27,28,29,30]. Selected myrmecophiles were extracted from the substrate using a Tullgren funnel and were then stored at −30 °C. Afterward, within 1–2 days after collecting, the tubes containing the insects were sent in dry ice to the Department of Genetics and Biosystematics, the University of Gdansk, where they were stored at −70 °C until further analysis.

### 2.2. DNA Extraction

DNA was extracted from whole insects using a universal Sherlock AX Purification Kit (A&A Biotechnology, Gdynia, Poland) based on spin column technology. Insects were rinsed three times in sterile distilled water prior to DNA extraction (without soaking in ethanol). Sterile equipment was used to avoid cross-contamination of samples. The quantity and quality of the extracted DNA were evaluated by using a NanoDrop ND-1000 spectrophotometer (NanoDrop Technologies Inc., Wilmington, Delaware, USA). After extraction, the DNA was stored at −20 °C until further use.

### 2.3. 16S rRNA Gene Amplification and Sequencing

Twenty-four samples consisting of specimens from six myrmecophilous beetles and red wood ants from two nests were used for further analyses (three individuals per species or nest). Negative controls have not been included. The V3-V4 hypervariable regions of the bacterial 16S rRNA gene were amplified using the following primer set: 341F (CCTACGGGNGGCWGCAG) and 785R (GACTACHVGGGTATCTAATCC). Libraries were prepared with a two-step PCR protocol based on Illumina’s “16S metagenomic library prep guide” (15044223 Rev. B), NEBNext^®^ Q5 Hotstart High-Fidelity DNA polymerase (New England BioLabs Inc., Ipswich, MA, USA), according to the manufacturer’s protocol using Q5^®^ Hot Start High-Fidelity 2X Master Mix (NEBNext—New England BioLabs, PCR conditions were as follows: 98 °C for 30 sec for initial denaturation, 98 °C for 10 s, 55 °C for 30 s, 72 °C for 20 s repeated for 25 cycles that was followed by a final extension at 72 °C for 2 min), and the Nextera Index kit (2 × 250 bp). Paired-end (PE, 2x250nt) sequencing with a 5% PhiX spike-in was performed with an Illumina MiSeq (MiSeq Reagent kit v2) at Genomed, Warsaw, Poland, following the manufacturer’s run protocols (Illumina, Inc., San Diego, CA, USA). The automatic primary analysis and the de-multiplexing of the raw reads were performed with MiSeq with the use of MiSeq Reporter (MSR) v2.6 (16S Metagenomics Protocol).

The data obtained for the independent sequencing were analyzed separately. Samples were marked as follows: DP-*L*—*D. pygmaeus*, LF-*D*—*L. formicetorum*, MA-*D*—*M. angusticollis*, MS-*L*—*M. subterraneus*, PF-*D*—*P. formicetorum*, TA-*L*—*T. angulata*, and FP-*D* and FP-*L* for *F. polyctena*. Indices D and L refer to the sampling sites (Dlugie Lake or Lipniak village, respectively).

The samples were processed and analyzed using the Quantitative Insights Into Microbial Ecology (QIIME, version 1.9.1) pipeline [31]. Paired-end reads from MiSeq sequencing were quality trimmed and joined with PANDAseq version 2.8 [32] with a quality threshold of 0.9. The sequences that did not meet the quality criteria were removed from further analysis (mean quality >20). Chimeric reads detection was performed with VSEARCH, version 1.7.0, an open-source replacement of USEARCH software. The clustering of operational taxonomic units (OTUs) at 97% similarity was performed by using the uclust method, version 1.2.22q [33]. Sample reads were rarefied to 8253 reads. OTUs were assigned to taxa using the SILVA v.132 database as the reference [34], with the taxonomy assignment tool PyNAST [35]. OTU saturation was evaluated with rarefaction curves using the Chao1 richness estimate. The Biological Observation Matrix (BIOM) table was used as the core data for downstream analyses [36]. Any sequences that were classified as Mitochondria or Chloroplast, as well as the singletons, were filtered out of the dataset. The alpha diversity indices, including Shannon, and Simpson indices and also the observed OTUs were estimated for each sample. The bacterial community structure was compared with the use of UniFrac [37] and Emperor [38]. A two-dimensional principal coordinate analysis (PCoA) was conducted from weighted UniFrac distances obtained from core diversity analyses. To determine if observed clusters were significantly dissimilar, an analysis of similarity (ANOSIM) was performed in QIIME with 999 permutations.

Similarity percentage (SIMPER) analysis was performed to calculate the average dissimilarities in bacterial community structures between samples and to access which family was responsible for the observed differences. For this purpose, all profiles were grouped according to the taxonomic position of the host. According to the Diss/SD values, we identified families, which were primarily responsible for the observed differences among profiles (larger number means more consistently contributes to the dissimilarity between profiles). Relative abundances of those bacterial families, which were primarily responsible for differences between samples, were then used for a proximity analysis (non-metric Multidimensional Scaling, nMDS). A similarity profile (SIMPROF) test was used to identify well-defined groups of samples [39]. Finally, to illustrate the most abundant bacterial families and microbiome relationships across tested samples, a heatmap and dendrogram were generated with the Bray–Curtis dissimilarity index. All statistical multivariate analyses were performed using Primer version 7 software [40].

The NGS data are deposited and fully available under study accession number PRJEB31343 in ENA—the European Nucleotide Archive.

## 3. Results

### 3.1. General Description of 16S rRNA Gene Sequencing Results

More than 8200 good quality sequences were obtained for each sample tested, ranging between 8253 (*T. angulata*, specimen TA1-*L*) and 150,363 (*F. polyctena*, specimen FP2-*D*). Chao1 curves almost reached a plateau, suggesting that the sequencing was approximately deep (Figure S1). At least 29 OTUs, ranging from 29-723, were observed in tested samples. High values of the Chao1 index and a number of identified OTUs, as well as high values of Shannon’s and Simpson’s indices, were observed for all tested DP-*L* specimens (*D. pygmaeus*), which suggests the high alpha diversity of those communities. More details of the sequence data for each species, as well as the number of observed OTUs and alpha diversity indices, are given in Table S1.

### 3.2. Bacterial Community Composition

**Figure 1:**
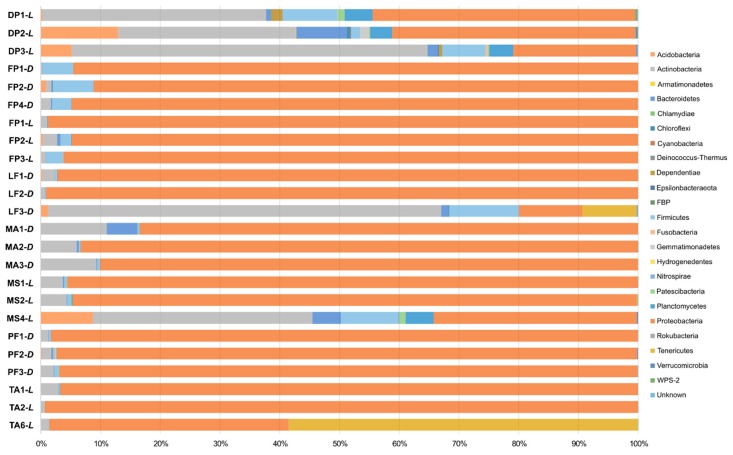
Abundance of bacterial 16S rRNA gene sequences at the phylum level.

Analysis of the microbial communities associated with the tested species showed that all the assigned reads were affiliated with Bacteria (Table S1). At the phylum level, at least 99.82% of all those sequences were classifiable, and the taxonomy-based analysis showed that the bacterial communities consisted of a total of 23 phyla (Figure 1 and Figure S2, Table S1). Proteobacteria, Actinobacteria, and Firmicutes were the most abundant phyla. They were observed in all samples tested and jointly accounted for more than 41% of the total microbial sequences obtained (in the range from 41.38% to 99.99%). The remaining reads in bacterial structure were associated with Acidobacteria, Armatimonadetes, Bacteroidetes, Chlamydiae, Chloroflexi, Cyanobacteria, Deinococcus-Thermus, Dependentiae, Epsilonbacteraeota, FBP, Fusobacteria, Gemmatimonadetes, Hydrogenedentes, Nitrospirae, Patescibacteria, Planctomycetes, Rokubacteria, Tenericutes, Verrucomicrobia, and WSP-2, with different contributions to the population (Figure 1, Table S1).

Relative abundances of identified bacterial groups at lower levels have been presented in interactive sunburst charts (Figure S2). The most numerous bacterial groups at lower taxonomic levels include: Alphaproteobacteria, Actinobacteria, Gammaproteobacteria, Planctomycetacia, and Bacilli at the class level and Rickettsiales, Corynebacteriales, Rhisobiales, Propionibacteriales, Streptomycethases, Acidobacteriales, and Betaproteobacteriales at the order level.

**Figure 2:**
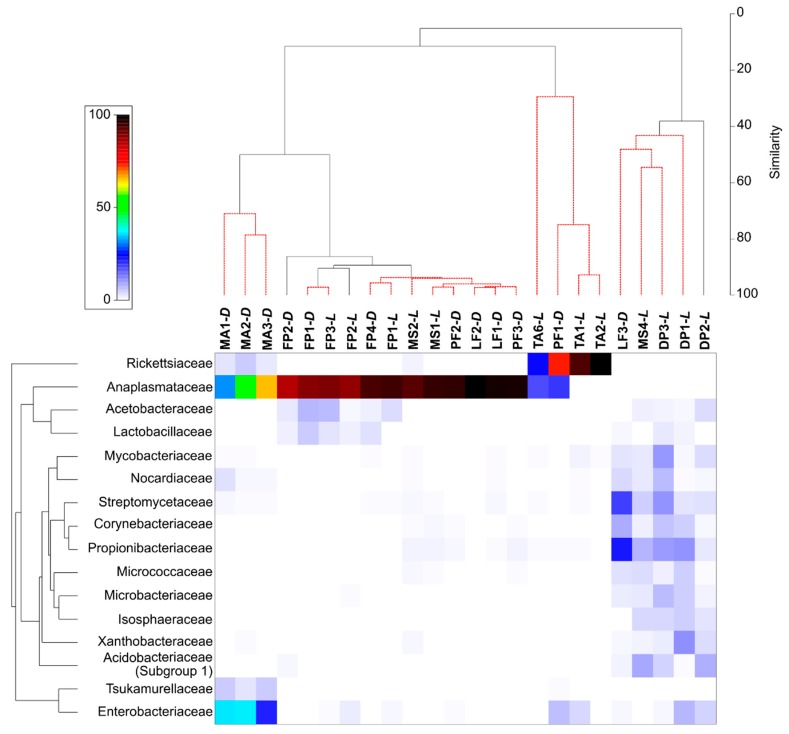
The heatmap showing bacterial families distributed across tested samples. Only those families who were primarily responsible for the observed differences among samples were considered. Both dendrograms were estimated with the Bray–Curtis dissimilarity index.

At the family level, Anaplasmataceae and Rickettsiaceae were generally the most abundant. However, the analysis of SIMPER results revealed that 16 families were primarily responsible for the observed differences among samples. Their relative abundances were indicated on the heatmap (Figure 2). All samples tested were clustered into five groups and three outliers by the SIMPROF test (*p* < 0.05; Figure 2). Five samples (DP1-*L*, DP2-*L*, DP3-*L*, LF3-*D*, and MS4-*L*) clustered together and were characterized, for example, by less abundant Anaplasmataceae, and more abundant Streptomycetaceae and Propionibacteriaceae. Bacterial communities associated with *M. angusticollis* also clustered together and were characterized by more abundant Enterobacteriaceae and Tsukamurellaceae (Figure S3).

**Figure 3:**
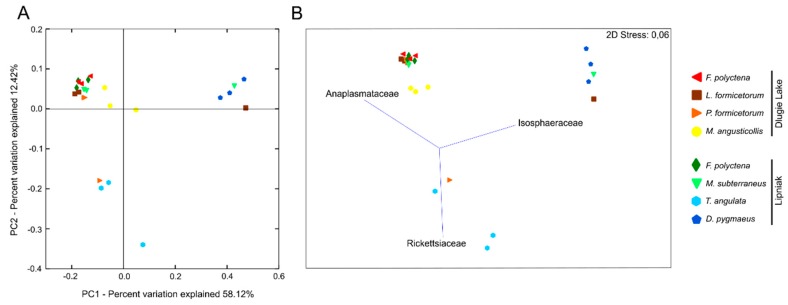
Principal Coordinate Analysis (PCoA) and Non-metric Multidimensional Scaling (nMDS) plots of the tested samples. (**A**) PCoA of bacterial communities associated with tested specimens based on weighted UniFrac distances; (**B**) nMDS based on Bray–Curtis dissimilarity between samples with identified vectors allowing to distinguish among clusters. Both plots show high overlap among *F. polyctena*, *L. formicetorum*, *P. formicetorum*, and *M. angusticollis* samples.

PCoA plot reveals that samples generally grouped into three distinct clusters (ANOSIM: R = 0.91, *p* = 0.001), which was congruent with SIMPROF results at lower levels of similarity of microbiota profiles (about 20%) (Figure 3A). Bacterial communities associated with *F. polyctena* (both from Dlugie Lake and Lipniak), *M. subterraneus* (except MS4-*L*), *L. formicetorum* (except LF3-*D*), and *P. formicetorum* (except PF1-*D*) were the most similar and clustered together. Nonparametric MDS analysis (85% similarity) revealed three vectors (Anaplasmataceae, Rickettsiaceae, and Isosphaeraceae), which allowed for distinguishing among clusters (Figure 3B).

Analysis at the genus-level revealed that *Wolbachia* and *Rickettsia* are the most abundant genera (Figure S2, Table S1). The highest relative abundance of *Wolbachia* has been noticed for *L. formicetorum* (98.75% in LF2-*D*). In the case of *T. angulata*, *Rickettsia* was more abundant than *Wolbachia* and accounted in the range of 22.63% for TA6-*L* to 99.01% for TA2-*L* (Figure S2, Table S1). Only in bacterial communities associated with *D. pygmaeus,* the relative abundances of those two endosymbionts did not exceed 1.00%.

**Figure 4:**
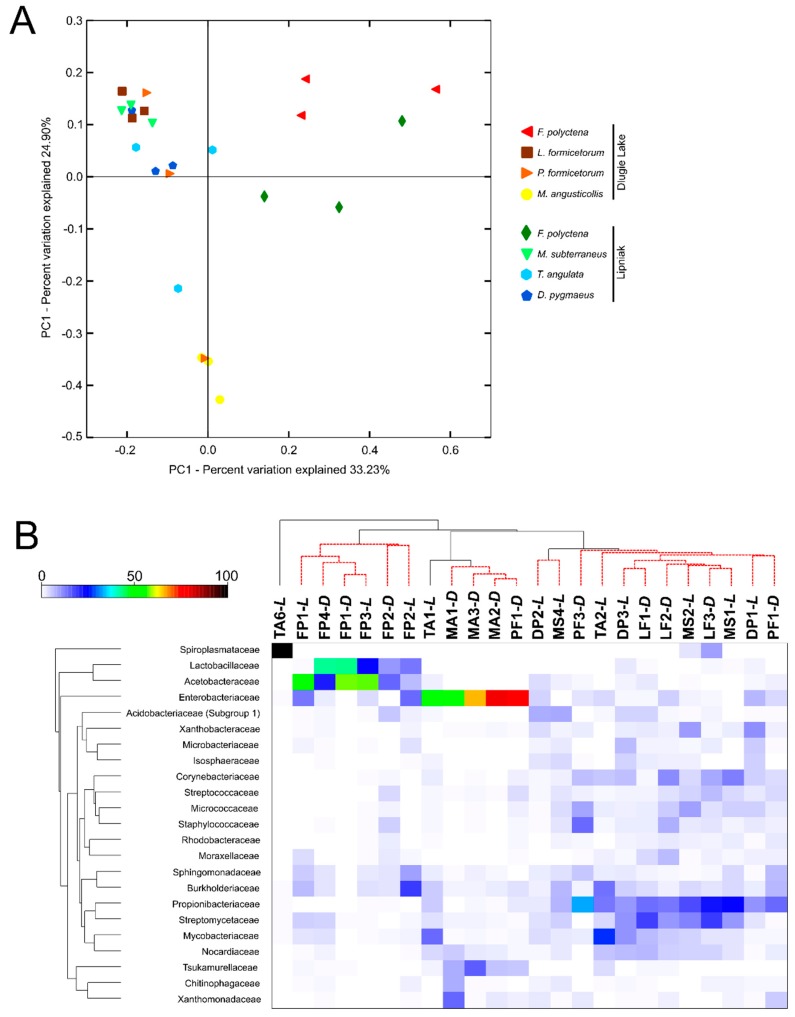
Principal Coordinate Analysis (PCoA) plot and the heatmap showing relationships among tested microbiome profiles after filtering *Wolbachia* and *Rickettsia* reads out. (**A**) PCoA of bacterial communities associated with tested specimens based on weighted UniFrac distances; (**B**) the heatmap showing bacterial families distributed across tested samples. Only those families who were primarily responsible for the observed differences among samples were considered. Both dendrograms were estimated with the Bray–Curtis dissimilarity index.

We could not a priori exclude that high relative abundance of *Wolbachia* and *Rickettsia* hid a host-specific sorting of analyzed microbiome profiles. To test this hypothesis, all analyses were repeated without *Wolbachia* and *Rickettsia* reads in the dataset. PCoA plot revealed that bacterial communities associated with *F. polyctena* differ from those associated with selected myrmecophiles (PERMANOVA: Pseudo-F = 7.7561, *p* = 0.001) (Figure 4A). That finding has been also supported by the result of a SIMPROF analysis that grouped all *F. polyctena* profiles in a homogenous cluster (Figure 4B). Values of ANOSIM calculated for pair-wise comparisons were significant only for pairs including *F. polyctena* (R = 0.846–0.994, *p* = 0.01). 

In all bacterial communities associated with ants, a higher relative abundance of Lactobacillaceae and Acetobacteraceae have been identified compared to other profiles tested (Diss/SD values calculated in SIMPER analysis were 1.44 and 1.73, respectively) (Figure 4B). In the case of bacterial communities associated with selected myrmecophiles, a species-specific separation has not been found and SIMPER analysis revealed that the abundance of Propionibacteriaceae and Streptomycetaceae make those profiles similar (calculated values of Sim/SD ware 0.93 and 0,75, respectively). Despite this general similarity of microbiota profiles associated with selected myrmecophiles, for *L. formicetorum*, *M. angusticollis*, and *F. polyctena,* the average similarity of profiles within species was higher than between species pair (calculated with PERMANOVA pair-wise test; Table 1). Nevertheless, only for *M. angusticollis* (MA1-*D*–MA3-*D*), we were able to connect this result with higher abundance of Enterobacteriaceae in its profiles (but profiles remained similar to *P. formicetorum* PF1-*D* profile and *T. angulata* TA1-*L* profile). The profiles separation according to the locality of the host’s nest has not been identified. (ANOSIM: R = 0.001, *p* = 0.366).

## 4. Discussion

Specialized interactions of bacterial communities with insect hosts are ubiquitous and often beneficial to insects [41]. Nevertheless, those communities may vary from simple to complex and can be influenced by environmental, genetic, and other factors of the host or host’s environment (e.g., host diet, phylogeny (coevolution), life stage, host location or pH) [42,43,44,45,46,47]. For many hosts, their phylogeny and diet have a strong effect on associated bacterial communities [48,49,50].

Although many studies have been focused on revealing the bacterial communities associated with insects, especially social species (e.g., [45,51,52,53,54,55,56,57,58,59]), still relatively little is known about potential similarities and differences among microbiomes of invertebrates, which are closely associated with ants. The study presented here gave the first insight into the bacterial population structure of *F. polyctena* and six unspecialized myrmecophilous beetle species commonly inhabiting its nests [19,20]. Identified microbiome profiles were compared with available data obtained for other known myrmecophiles and their hosts. Nevertheless, it is worth mentioning that such studies are limited and myrmecophiles from five families selected here have not been included in them previously.

Microbiota analyses bear a risk of sample contamination. Thus, it is reasonable to analyze additional negative and positive samples, which was pointed out by Hornung et al. (2019) [60]. Although we did not use negative controls in obtaining libraries, we have made every effort to minimize the risk of contamination. We found that all tested bacterial communities were predominated by three phyla (Proteobacteria, Actinobacteria, and Firmicutes), with each comprising a different share of the microbiome depending on species or sample tested (Table S1). Those phyla are frequently listed as the most abundant in bacterial communities associated with insect taxa [61,62,63,64], including ants [49,65,66,67,68,69,70] and myrmecophilous caterpillars of Lycaenidae butterflies [71,72,73]. In some cases, the joint abundance of those phyla may exceed even 90% of bacterial community membership [63]. Such high values were also observed in the presented study. However, for most samples, only Proteobacteria was predominant. That phylum has also been identified as a significant part of the bacterial communities associated with the leaf-cutting ants genera *Atta* and *Acromyrmex* [65], the plant-ant genera *Allomerus* and *Tetraponera* [74], the turtle ant *Cephalotes rohweli* [67] or the weaver ant *Oecophylla smaragdina* [75]. Interestingly, for *D. pygmaeus* samples, Proteobacteria was less abundant (<44%) and Actinobacteria was more frequently identified than in the rest of bacterial communities tested (>29%) (Table S1).

The high relative abundance of Actinobacteria and Proteobacteria could be explained through the prism of previous studies. Members of Actinobacteria are able to protect different insect species (e.g., ants and beetles) from pathogens by excretion of substances with antibiotic activity [76]. In our study, *Pseudonocardia* and *Streptomyces* have been found (Table S1). Those beneficial bacteria are reared especially by higher fungus-farming ants and housed in their cuticular crypts, tubercles, and other modifications associated with subcuticular exocrine glands [77]. Both *Pseudonocardia* and *Streptomyces* are known to produce antibiotic substances and protect nests and their hosts against numerous bacterial and fungal microbes [78,79,80]. These metabolites are primarily active against *Escovopsis*, a potentially virulent, specialized fungus that attacks the ants’ mutualistic fungus [77,81]. In addition, antibiotics from these bacteria, together with the metapleural gland, contribute to ants’ immune system against their pathogens [65]. Although *F. polyctena* and selected myrmecophiles are not involved in fungus farming, their food resources and brood are constantly in contact with the soil and prone to fungal invasion. Therefore, the protective role of identified Actinobacteria in the host-myrmecophile relationship seems likely. Such activity has been described previously in studies on bacteria associated with other ant species, also *Formica* representatives [65,74,76,82,83,84,85,86].

In the case of Proteobacteria, the explanation seems to be different. Those bacteria are known to be metabolically versatile or specialized and therefore adapted to complex environments, such as the gut of social insects [87]. Therefore, their high abundance in almost all tested samples should not raise suspicions. In bacterial communities associated with *F. polyctena* we found an increased abundance of Acetobacteraceae (Figure 4B). Members of this family have been identified in microbiome profiles of ants feeding on carbohydrate-rich diets, including honeydew-feeding *Camponotus* carpenter ants, *Formica* wood ants, *Linepithema* Argentine ants [88,89,90], and numerous insect species relying on sugar-based diets [91,92]. That increased abundance may be primarily related to the presence of the honeydew in the *F. polyctena* diet. However, the acetic acid bacteria (AAB) have also been shown to actively colonize different insect tissues and organs and being involved in the regulation of the innate immune system homeostasis [93].

Although members of the Acetobacteraceae family were abundant in microbiome profiles of *F. polyctena,* all tested bacterial communities were primarily predominated by other Proteobacteria members—Anaplasmataceae and Rickettsiaceae (Table S1, Figure S2). The predominance of those two families has been directly connected with a high relative abundance of *Wolbachia* and *Rickettsia* endosymbionts. The former potentially infects more than 65% of insect species [94,95] and a wide range of ant genera, for example, *Atta* and *Acromyrmex* [96,97,98], *Camponotus* [99,100], *Cephalotes* [101,102], *Colobopsis* [103], *Formica* [104,105], *Polyrhachis* [49], *Solenopsis* [106,107,108], and others [109,110,111]. *Wolbachia* has been also identified with high relative abundance in bacterial communities associated with the myrmecophilous larvae of *Maculinea alcon* butterfly [71]. As *Wolbachia* could well represent the most widespread symbiont in insects, it remains at the center of researchers’ interests. This endosymbiont acts like a detrimental reproductive manipulator because it can cause the cytoplasmic incompatibility, parthenogenesis, male killing, or male feminization [112,113,114,115]. There are also examples showing that *Wolbachia* protects its host against RNA viruses [116,117]. However, its function in ants is unknown, especially in the sterile workers, who are not able to reproduce [118,119,120]. On the other hand, some evidence suggests that *Wolbachia* may influence sex ratios of these social insects [110].

Nevertheless, the identification of *Wolbachia* endosymbiont may raise the question about its potential horizontal transmission among *F. polyctena* and selected myrmecophiles. This phenomenon may occur very frequently in ants and has been described previously by Reuter et al. (2005) [121] for three ant genera *Linepithema*, *Acromyrmex*, and *Solenopsis* located in Latin America. However, a recent study showed that not all *Wolbachia* strains associated with ants have the same genetic potential for horizontal transmission [122]. Here, we cannot unambiguously state whether endosymbiont is horizontally transmitted among *F. polyctena* and associated myrmecophiles. However, this hypothesis seems likely, because all specimens share the same identified OTUs. At this stage of research, we cannot exclude both direct and indirect transfer. The direct horizontal transfer has been discussed by Dedeine et al. (2005) [123] who investigated *Wolbachia* variants infecting fly parasitoids, inquiline social parasite *Solenopsis daguerrei*, and their hosts (*Solenopsis invicta* and *S. richteri*, respectively), revealing identical symbiont variants shared among *S. daguerrei* and both its hosts. Interaction between the social parasite and its host (such as trophallaxis, egg carrying, or brood feeding) may provide opportunities for the transmission of *Wolbachia* from the host to the social parasite and the other way around [123]. Myrmecophiles, of which microbiome profiles were analyzed in the present study, may acquire endosymbiont in a similar way. As predators and scavengers, they feed on dead hosts or their brood, which may constitute the infection source. The other possible transmission way may be environmentally-mediated. Such examples have been noted, for example, in intertidal amphipod crustaceans or butterflies sharing the same habitat [124,125]. Moreover, a recent study has also confirmed the possibility of horizontal transmission through food sources [126,127]. 

Although all hypotheses mentioned above seem likely, the reason for the lack of infection in *D. pygmaeus* and *T. angulata* remains unsolved. Both species feed on dead ants or their brood and live in close relationships with *F. polyctena*, which would allow expecting that *Wolbachia* will be present in their microbiome profiles as well. However, the results presented here have been obtained for limited samples, and therefore, we cannot exclude that both *D. pygmaeus* and *T. angulata* are infected with *Wolbachia* in a smaller percentage than the other selected species. Moreover, we cannot unambiguously reject the possibility that native microbiota of these two myrmecophiles impede *Wolbachia* transmission. Such observations have been described previously for *Anopheles* mosquitoes [128].

The second known endosymbiotic Proteobacteria member found here with a higher relative abundance was *Rickettsia*. Like *Wolbachia*, *Rickettsia* can also influence the biology of its insect hosts. As vertically transmitted endosymbiont, it is known to have diverse effects on hosts, ranging from influencing host fitness to manipulating its reproduction [129]. Recent studies have shown that *Rickettsia*-containing populations are more tolerant of heat shock than those without this endosymbiont [130]. *Rickettsia*-infected species can also produce more offspring, have a greater survival rate to adulthood, develop faster, and produce a higher proportion of female offspring [131]. Moreover, the presence of *Rickettsia* can be linked to host protection against entomopathogenic bacteria, for example, *Pseudomonas syringae* [132] or a higher level of susceptibility to insecticides [117].

In the present study, *Rickettsia* has been identified in bacterial communities associated with six selected beetle species, comprising a different share of the microbiome depending on the beetle host. However, *Rickettsia* was not found in *F. polyctena* bacterial communities. A similar result has been obtained for another *Formica* ant, *F. cinerea* [133]. In the case of selected beetles, the highest values of *Rickettsia* abundance have been observed for *T. angulata* (range from 22.63 for TA6-*L* to 99.01% for TA2-*L*). In the rest of the tested microbiome profiles that endosymbiont counted for was less than 5% (Table S1). Interestingly, in microbial communities of *M. angusticollis* (MA1-*D*–MA3-*D*), *P. formicetorum* (PF1-*D*) and *T. angulata* (TA6-*L*) *Rickettsia* coexist with *Wolbachia*, which was identified previously for other beetles [134,135,136].

The variation in microbiota in insects is related to many factors. The microbial community can be determined by the host phylogeny, gut morphology, and physic-chemical conditions, such as pH and oxygen availability in the insect gut [137,138]. These and other factors (e.g., developmental stage and diet) were also identified shaping ants’ microbiota [47,49,103,139,140]. In the present study, the bacterial communities associated with selected insects have been influenced also by insects’ dietary habits and other unrevealed biotic or abiotic factors. However, the indirect aim of the presented study was to identify a diagnostic and universal microbiome profile that would be characteristic for myrmecophiles inhabiting *F. polyctena* nests and belonging to the same trophic level. Such a study has been performed previously for predatory and herbivorous ant species [48]. Nevertheless, the relationships among the microbiome profiles identified here were complex, and no relative abundance pattern that would be common to all species was observed. Bacterial communities associated with most selected species (except *D. pygmaeus*, *M. angusticollis*, and *T. angulata*) cluster together, but the observed similarities are rather the results of *Wolbachia* infection than the common microbiome pattern. This trend has been shown in Figure 3B, where Anaplasmataceae is identified as one of the main grouping vectors. Some subtle, species-specific patterns have been observed for bacterial communities associated with *D. pygmaeus*, *M. angusticollis*, and *T. angulata* (Figure 1). Filtering out *Wolbachia* and *Rickettsia* reads from the analyzed dataset revealed that all *F. polyctena* samples cluster together (Figure 3B), and further analyses revealed that Lactobacillaceae and Acetobacteraceae were primarily responsible for observed similarity. In the case of bacterial communities associated with selected beetles, SIMPER analysis revealed that Propionibacteriaceae and Streptomycetaceae are generally more abundant in them. However, ANOSIM and PERMANOVA tests did not support the presence of more complex, species-specific sorting, and sample separation according to the locality of the host’s nest. Thus, at this stage of the study, we cannot identify a common and stable microbiota profile, which would be characteristic for myrmecophilous beetles.

## 5. Conclusions

This paper presents the results of the case study and analyses of bacterial communities associated with *F. polyctena* and six myrmecophilous beetles inhabiting its nests. The host phylogeny does not directly affect the composition of the microbiome. Most of the analyzed microbiome profiles were clustering together, but it might be strictly correlated with a high relative abundance of *Wolbachia* reads. This endosymbiont has been previously identified for *F. polyctena*, but here, we identified its occurrence in the microbiome profiles of six myrmecophilous beetles for the first time. Furthermore, for all species except *F. polyctena*, the *Rickettsia* endosymbiosis has been identified. However, after filtering out *Wolbachia* and *Rickettsia* reads, we revealed that bacterial communities associated with *F. polyctena* differ from those associated with selected myrmecophiles. Nevertheless, microbiota profiles of selected myrmecophiles were not homogenous.

## Figures and Tables

**Table 1:** Average similarity between and within groups of identified microbiota profiles. Profiles were grouped according to host species.

Host Species	*D. pygmaeus*	*F. polyctena*	*L. formicetorum*	*M. angusticollis*	*M. subterraneus*	*P. formicetorum*	*T. angulata*
*D. pygmaeus*	51.066						
*F. polyctena*	22.568	48.954					
*L. formicetorum*	51.785	17.534	65.864				
*M. angusticollis*	16.151	11.77	13.216	77.465			
*M. subterraneus*	53.743	18.352	62.895	11.372	58.866		
*P. formicetorum*	35.998	16.628	40.068	35.943	43.313	33.288	
*T. angulata*	28.933	13.482	30.611	27.323	28.206	26.899	18.352

## Supplementary Materials

The following are available online at https://www.mdpi.com/2075-4450/11/2/134/s1, Figure S1: Rarefaction curves with Chao1 diversity indices, indicating insect microbiome sampling depth and saturation, Figure S2: Detailed taxonomic analyses at different ranks for DNA. Sunburst charts show the relative abundance of microbial 16S rDNA sequences at different taxonomic levels. The first level represents the kingdom; the second level represents all phyla present in a particular sample; subsequent next levels represent the class, order, family and genus, Figure S3: Relative abundances of bacterial families primarily responsible for observed differences among profiles, Table S1: Sheet 1—Summary of the sequencing data and statistical analysis of bacterial communities associated with *F. polyctena* and selected myrmecophilous beetles. Sheets 2-6—Detailed taxonomic analyses at different ranks with the data obtained for the samples tested. The table shows the relative abundance of microbial 16S rDNA sequences at different taxonomic levels.
