# Supplementary figures and images for: First Insight into Microbiome Profiles of Myrmecophilous Beetles and Their Host, Red Wood Ant Formica polyctena (Hymenoptera: Formicidae)—A Case Study
Source: Insects. 2020 Feb 19;11(2):134. doi: 10.3390/insects11020134 (PMC7073670; doi:10.3390/insects11020134)

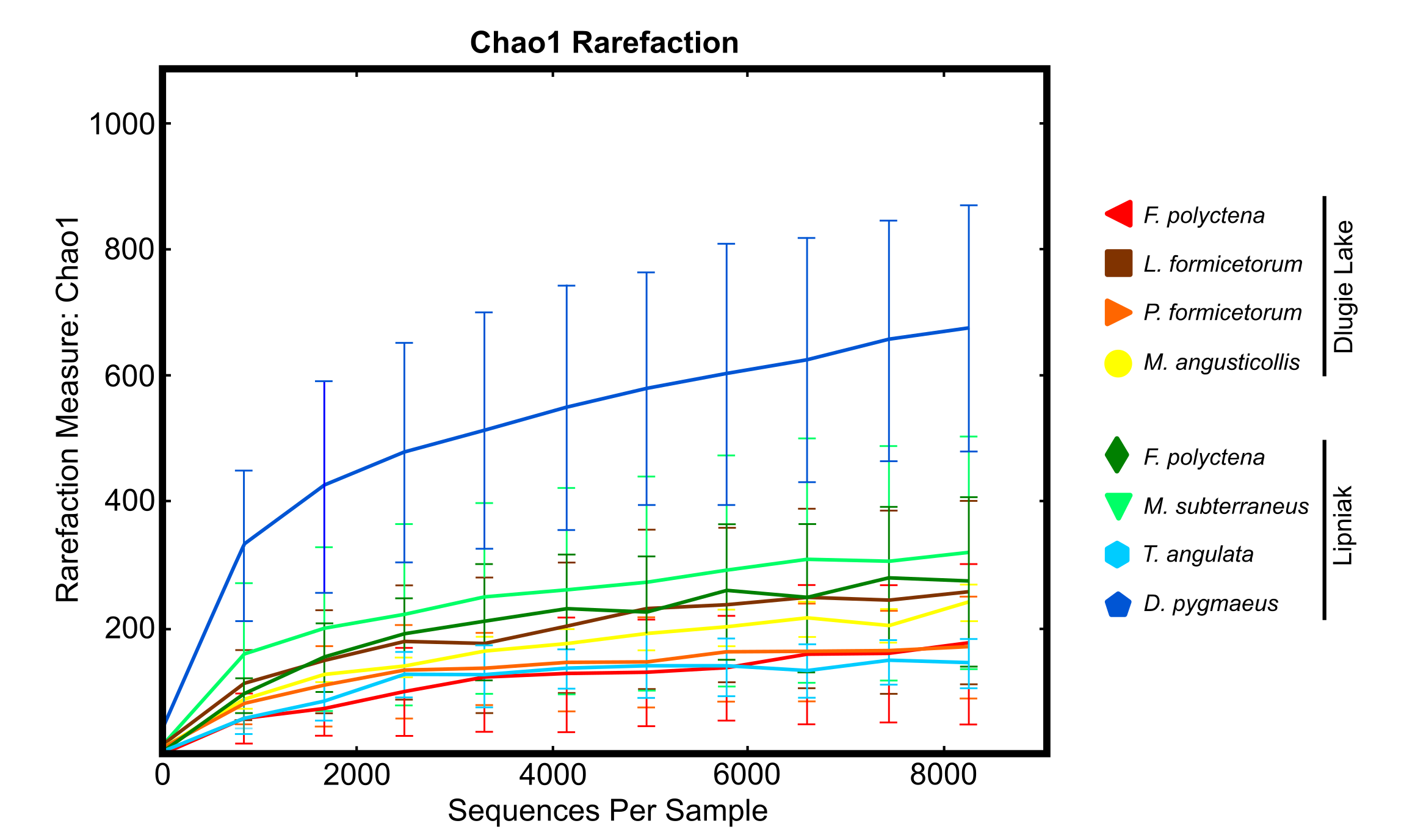

Supplement: Supplementary file 1 [file insects-11-00134-s001.zip › Supplementary Files/S1_Figure.png]

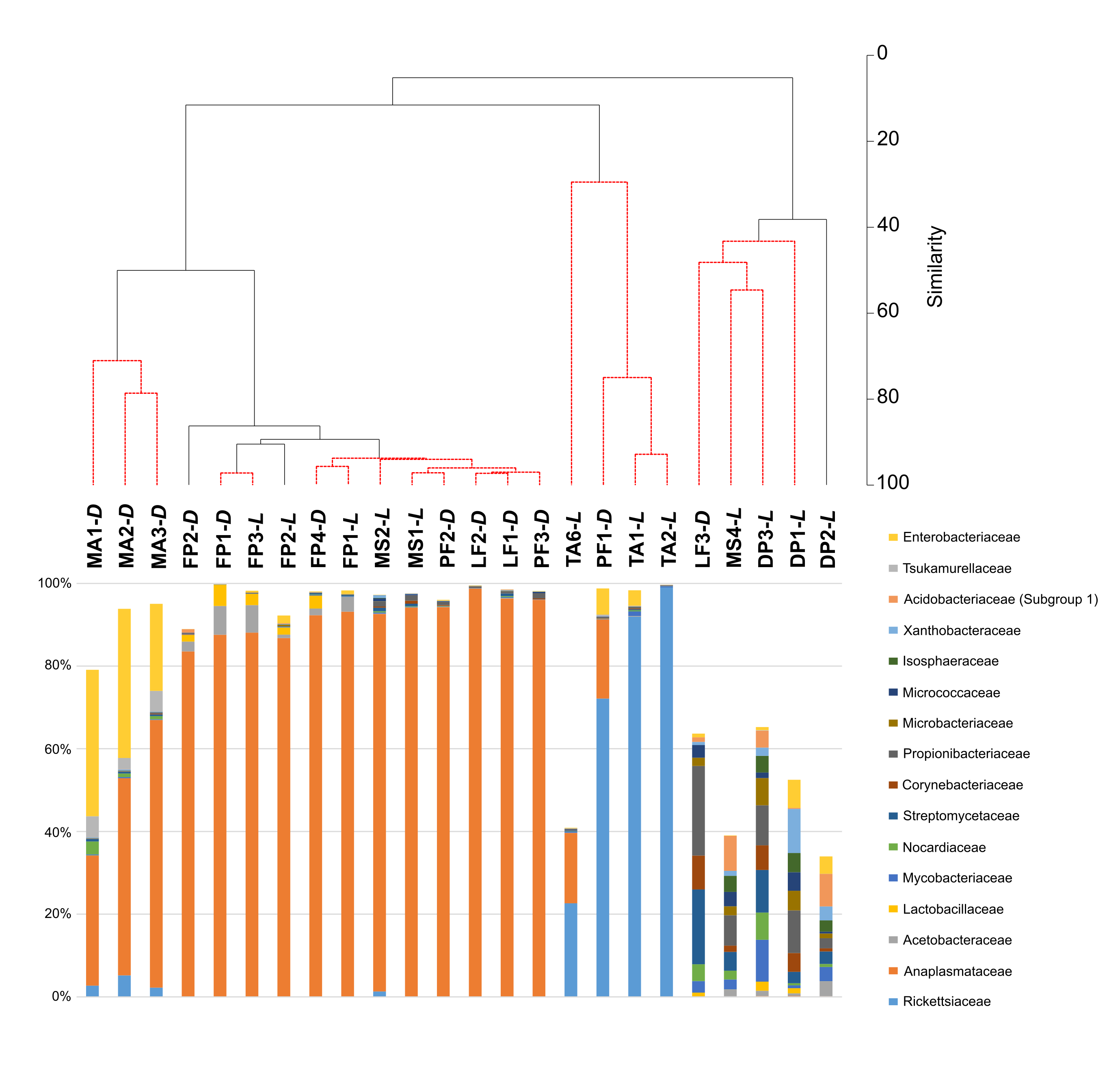

Supplement: Supplementary file 1 [file insects-11-00134-s001.zip › Supplementary Files/S3_Figure.png]
