# Supplementary material for: First Insight into Microbiome Profiles of Myrmecophilous Beetles and Their Host, Red Wood Ant Formica polyctena (Hymenoptera: Formicidae)—A Case Study
Source: Insects. 2020 Feb 19;11(2):134. doi: 10.3390/insects11020134 (PMC7073670; doi:10.3390/insects11020134)
Supplement: Supplementary file 1 [file insects-11-00134-s001.zip › Supplementary Files/S2_Figure.zip!/Supplemental_Figure_S2/Supplemental_Figure_S2_DP1.html]

Javascript must be enabled to view this page.

magnitude

 .999999999999706

 .999999999999706

 8.809619486258E-04

 8.809619486258E-04

 8.809619486258E-04

 8.809619486258E-04

 3.09109455658E-05

 .00085005100306

 2.3492318630008E-03

 2.3492318630008E-03

 2.3492318630008E-03

 2.3492318630008E-03

 .0011746159315

 0

 0

 3.09109455658E-05

 0

 .000494575129053

 0

 0

 .000649129856882

 0

 0

 0

 0

 0

 0

 0

 0

 0

 0

 0

 0

 0

 0

 0

 0

 0

 0

 0

 0

 0

 0

 0

 0

 0

 0

 0

 0

 0

 0

 0

 0

 0

 0

 0

 0

 0

 0

 0

 0

 0

 0

 0

 0

 0

 0

 0

 0

 0

 0

 0

 0

 0

 0

 0

 0

 0

 0

 0

 0

 0

 0

 0

 0

 0

 0

 0

 0

 0

 0

 0

 0

 .374687026675658

 0

 0

 0

 0

 .008747797595126

 0

 0

 0

 0

 .001097338567587

 .000154554727829

 .000154554727829

 0

 0

 0

 0

 .000942783839758

 .000942783839758

 0

 0

 0

 0

 0

 0

 0

 0

 0

 0

 0

 .007650459027539

 .000633674384099

 .000633674384099

 0

 0

 .00701678464344

 .00701678464344

 0

 0

 .351828382429735

 0

 0

 0

 .00336929306667

 .00336929306667

 .00336929306667

 0

 0

 0

 0

 .00389477914129

 .00389477914129

 .00389477914129

 .057679824425845

 .04567092207352

 0

 0

 .0265679577138

 .00179283484282

 .0173101295169

 0

 0

 0

 0

 .0067849525517

 .0067849525517

 .005223949800625

 0

 .00455936447096

 0

 0

 0

 0

 .000664585329665

 0

 0

 0

 0

 0

 0

 .04299712528209

 0

 0

 .00846959908504

 .00846959908504

 0

 0

 0

 .0314982535316

 0

 .0314982535316

 0

 0

 0

 0

 0

 .00302927266545

 .00302927266545

 0

 0

 0

 0

 0

 0

 0

 0

 0

 0

 0

 0

 0

 0

 9.90232141200499E-02

 0

 0

 0

 0

 0

 0

 0

 0

 1.4373589688158E-03

 0

 0

 0

 0

 3.09109455658E-05

 0

 .00140644802325

 0

 0

 .00404933386912

 .00404933386912

 0

 1.1746159314987E-03

 .00102006120367

 0

 1.54554727829E-05

 0

 0

 3.09109455658E-05

 .00010818830948

 4.75719452257718E-02

 .0269234335878

 0

 3.09109455658E-05

 4.63664183487E-05

 .000123643782263

 1.54554727829E-05

 0

 0

 0

 .00905690705079

 0

 .00128280424098

 .00377113535903

 9.27328366975E-05

 6.18218911316E-05

 .000154554727829

 .00282835151927

 0

 1.54554727829E-05

 1.54554727829E-05

 0

 9.27328366975E-05

 .00306018361102

 4.47899601248436E-02

 3.09109455658E-05

 .000278198510092

 .00369385799512

 .0407560817285

 0

 0

 0

 3.09109455658E-05

 0

 0

 0

 0

 0

 0

 0

 0

 0

 0

 0

 0

 .11378319062739

 .01086519736639

 .00315291644771

 .00279744057371

 0

 .0019010231523

 .00301381719267

 .102917993261

 .102917993261

 0

 0

 0

 0

 .0035238477945

 .0035238477945

 0

 0

 0

 0

 0

 .0035238477945

 0

 .0275571079719

 .0275571079719

 0

 0

 .0275571079719

 0

 0

 0

 0

 0

 0

 0

 0

 0

 0

 0

 0

 0

 0

 0

 .014110846650797

 .00344657043059

 0

 0

 .00344657043059

 .00179283484282

 0

 0

 .00165373558777

 .010664276220207

 0

 0

 .010076968254456

 .0096751259621

 .000401842292356

 .000587307965751

 0

 .000587307965751

 0

 0

 0

 0

 0

 0

 0

 0

 0

 0

 0

 0

 0

 0

 0

 0

 0

 0

 0

 0

 8.4077771939067E-03

 8.4077771939067E-03

 .000525486074619

 0

 0

 0

 0

 .000525486074619

 0

 .000525486074619

 0

 0

 0

 .007217705789623

 0

 0

 .007217705789623

 0

 0

 0

 0

 0

 0

 0

 0

 0

 0

 0

 .000757318166363

 0

 0

 0

 .00646038762326

 0

 0

 0

 0

 0

 0

 0

 0

 0

 0

 0

 6.491298568818E-04

 0

 0

 1.54554727829E-05

 0

 1.54554727829E-05

 0

 0

 6.336743840989E-04

 0

 1.54554727829E-05

 .000618218911316

 0

 1.54554727829E-05

 0

 0

 0

 0

 1.54554727829E-05

 0

 0

 1.54554727829E-05

 0

 0

 0

 0

 0

 0

 0

 0

 1.700102006117E-04

 1.700102006117E-04

 1.700102006117E-04

 0

 0

 1.700102006117E-04

 .000123643782263

 4.63664183487E-05

 0

 0

 0

 0

 0

 0

 0

 0

 0

 0

 0

 0

 0

 0

 0

 0

 0

 0

 0

 0

 0

 0

 0

 0

 0

 0

 0

 0

 0

 0

 0

 0

 0

 0

 0

 0

 0

 0

 0

 0

 0

 0

 0

 0

 0

 0

 0

 0

 0

 0

 0

 0

 0

 0

 0

 0

 0

 0

 0

 0

 0

 0

 0

 0

 0

 0

 0

 0

 0

 0

 0

 0

 0

 0

 0

 0

 0

 0

 0

 0

 0

 0

 0

 0

 0

 .018902043213551

 .018902043213551

 .018902043213551

 0

 0

 .000587307965751

 .000587307965751

 .0183147352478

 0

 .0183147352478

 0

 0

 0

 0

 0

 0

 0

 0

 0

 0

 0

 0

 0

 0

 9.17745973850125E-02

 7.53917962351017E-02

 3.38629408673497E-02

 3.09109455658E-05

 3.09109455658E-05

 .00567215851133

 .00567215851133

 0

 0

 0

 0

 0

 0

 .00632128836821

 .00632128836821

 0

 0

 0

 0

 0

 3.400204012239E-04

 0

 0

 .000324564928441

 1.54554727829E-05

 0

 0

 0

 0

 0

 0

 0

 0

 0

 0

 .02149856264102

 .00179283484282

 0

 .0197057277982

 0

 0

 .041528855367752

 0

 0

 0

 0

 0

 .000540941547402

 0

 .000540941547402

 .00746499335415

 0

 .00746499335415

 .0125189329542

 .0125189329542

 0

 0

 .021003987512

 0

 .021003987512

 1.57182158202458E-02

 1.57182158202458E-02

 0

 0

 0

 0

 0

 0

 0

 0

 .01568730487468

 .0142190349603

 0

 0

 0

 .00146826991438

 0

 0

 0

 0

 0

 0

 0

 0

 0

 0

 0

 0

 0

 3.09109455658E-05

 0

 1.54554727829E-05

 0

 0

 1.54554727829E-05

 0

 .000664585329665

 .000664585329665

 .000664585329665

 0

 .000664585329665

 0

 0

 0

 0

 0

 0

 0

 0

 0

 0

 0

 0

 0

 0

 0

 0

 7.418626935797E-04

 7.418626935797E-04

 7.418626935797E-04

 7.418626935797E-04

 4.63664183487E-05

 .000695496275231

 0

 0

 0

 0

 0

 0

 0

 0

 0

 0

 0

 0

 0

 0

 1.16843374238858E-02

 0

 0

 0

 0

 0

 0

 0

 0

 0

 0

 0

 0

 1.16843374238858E-02

 1.16843374238858E-02

 1.54554727829E-05

 1.54554727829E-05

 0

 0

 0

 0

 0

 1.54554727829E-05

 1.54554727829E-05

 .00179283484282

 .00179283484282

 .0098605916355

 .0098605916355

 0

 0

 0

 0

 0

 0

 .046845538004964

 .00037093134679

 0

 0

 0

 0

 .00037093134679

 0

 0

 .00037093134679

 .00037093134679

 0

 0

 0

 0

 0

 0

 0

 0

 .046474606658174

 0

 0

 0

 0

 .046474606658174

 .046474606658174

 .0135544496306

 .00757318166363

 0

 .0119470804612

 .000231832091744

 .013168062811

 0

 0

 0

 0

 0

 0

 0

 0

 0

 0

 .43966183425562

 .179716237519711

 6.1049117492529E-03

 6.1049117492529E-03

 1.54554727829E-05

 0

 .000571852492968

 .000587307965751

 0

 0

 0

 0

 0

 0

 0

 0

 .000958239312541

 0

 0

 0

 0

 .00397205650521

 0

 0

 0

 0

 0

 0

 0

 2.6737967914416E-03

 2.6737967914416E-03

 0

 0

 6.18218911316E-05

 0

 .00261197490031

 0

 0

 0

 0

 0

 0

 0

 0

 0

 0

 0

 0

 0

 0

 0

 0

 0

 0

 0

 0

 0

 0

 0

 0

 0

 0

 0

 0

 0

 0

 0

 0

 .00539396000124

 .00539396000124

 .00539396000124

 0

 .153689221353291

 0

 0

 0

 0

 .020107570090589

 0

 .000417297765139

 0

 0

 .0013600816049

 0

 0

 0

 0

 .00655312045995

 .0117770702606

 0

 0

 4.5902754165235E-03

 .00350839232172

 0

 9.27328366975E-05

 .000989150258106

 .00426571048808

 .00426571048808

 0

 0

 0

 0

 0

 0

 0

 0

 0

 0

 0

 1.68310098605909E-02

 .000772773639146

 7.72773639146E-05

 0

 1.54554727829E-05

 4.63664183487E-05

 .00999969089054

 0

 0

 1.54554727829E-05

 3.09109455658E-05

 0

 0

 .00480665203549

 .00106642762202

 .00109733856759

 .00109733856759

 0

 0

 0

 0

 .106797316929918

 .0239714382863

 .0717133937127

 0

 .00125189329542

 0

 .000139099255046

 .00242650922692

 .000170010200612

 0

 .00712497295292

 0

 0

 0

 0

 4.1111557602545E-03

 4.1111557602545E-03

 0

 0

 1.54554727829E-05

 .00188556767952

 .00214831071682

 0

 0

 6.18218911316E-05

 0

 0

 0

 0

 0

 0

 0

 0

 0

 0

 .002256499026301

 0

 0

 .000958239312541

 .000958239312541

 .00129825971376

 0

 .00129825971376

 0

 .00548669283793

 .00548669283793

 0

 0

 0

 0

 0

 0

 0

 0

 0

 0

 .00548669283793

 0

 0

 0

 0

 0

 0

 0

 0

 0

 0

 0

 0

 0

 .02132855244038

 0

 0

 0

 .00213285524404

 0

 0

 0

 .00213285524404

 .00213285524404

 0

 0

 0

 0

 .01919569719634

 0

 0

 0

 0

 0

 0

 0

 0

 .00295199530154

 .00295199530154

 0

 0

 0

 0

 0

 0

 0

 0

 0

 0

 0

 .0162437018948

 .0162437018948

 0

 0

 0

 0

 0

 0

 0

 0

 0

 0

 0

 .238617044295529

 0

 0

 0

 .111356681401

 .111356681401

 .111356681401

 0

 0

 0

 0

 0

 2.47442119254469E-02

 0

 0

 2.05712342740581E-02

 .00737226051745

 0

 0

 0

 0

 0

 .0055176037835

 6.18218911316E-05

 0

 .00625946647708

 9.27328366975E-05

 0

 0

 0

 0

 0

 0

 .000154554727829

 0

 0

 0

 0

 0

 0

 0

 .00111279404037

 0

 0

 0

 0

 0

 0

 0

 0

 0

 0

 0

 0

 .000386386819573

 0

 .000386386819573

 0

 0

 0

 3.09109455658E-05

 0

 3.09109455658E-05

 0

 0

 0

 0

 0

 0

 .00375567988625

 0

 .00010818830948

 0

 .00364749157677

 0

 0

 0

 0

 0

 0

 0

 0

 0

 0

 .000880961948626

 .000880961948626

 .000880961948626

 0

 0

 6.79113474081124E-02

 6.79113474081124E-02

 .0416834100955

 .00344657043059

 0

 0

 .00550214831072

 1.54554727829E-05

 .00808321226546

 0

 .00544032641959

 6.18218911316E-05

 0

 0

 1.54554727829E-05

 9.27328366975E-05

 0

 .00140644802325

 9.27328366975E-05

 0

 .00207103335291

 0

 0

 0

 0

 0

 0

 0

 0

 .00026274303731

 .00026274303731

 .00026274303731

 .00319928286606

 .00319928286606

 .00319928286606

 0

 0

 0

 0

 0

 .000757318166363

 .000757318166363

 .000757318166363

 .00675404160613

 .00675404160613

 .00675404160613

 .0196593613799

 .01678464344228

 0

 0

 .00010818830948

 0

 .0166764551328

 0

 .00287471793762

 .00287471793762

 0

 0

 0

 4.63664183487E-05

 4.63664183487E-05

 0

 4.63664183487E-05

 0

 0

 0

 0

 0

 0

 0

 0

 0

 0

 0

 0

 3.0447281382316E-03

 1.54554727829E-05

 0

 0

 1.54554727829E-05

 0

 0

 0

 0

 3.0292726654487E-03

 0

 0

 0

 0

 3.09109455658E-05

 1.54554727829E-05

 .0029829062471

 0

 0

 0

 0

 0

 0

 0

 0

 0

 0

 0

 0

 0

 0

 0

 0

 0

 0

 0

 0

 0

 0

 0

 0

 0

 0

 0

 0

 0

 0

 0

 0

 0

 0

 0

 0

 0

 0

 0

 0

 0

 0

 0

 0

 0

 0

 0

 0

 0

 0

 0

 0

 0

 0

 0

 0

 0

 .00389477914129

 .00281289604649

 .00281289604649

 .00281289604649

 .00281289604649

 .0010818830948

 .0010818830948

 .0010818830948

 .0010818830948
