# Supplementary material for: First Insight into Microbiome Profiles of Myrmecophilous Beetles and Their Host, Red Wood Ant Formica polyctena (Hymenoptera: Formicidae)—A Case Study
Source: Insects. 2020 Feb 19;11(2):134. doi: 10.3390/insects11020134 (PMC7073670; doi:10.3390/insects11020134)
Supplement: Supplementary file 1 [file insects-11-00134-s001.zip › Supplementary Files/S2_Figure.zip!/Supplemental_Figure_S2/Supplemental_Figure_S2_DP2.html]

Javascript must be enabled to view this page.

magnitude

 .999999999999888

 .999999999999888

 .000153407845716

 .000153407845716

 .000153407845716

 .000153407845716

 0

 .000153407845716

 .129629629629637

 .12721893491125

 8.07363576594626E-02

 .078961209730469

 .0261012491782

 2.19154065308E-05

 .0113740959895

 8.76616261232E-05

 .0170721016875

 .00293666447513

 0

 .000701293008985

 .0206662283585

 .000525969756739

 .000525969756739

 1.2491781722546E-03

 4.38308130616E-05

 .000788954635108

 .000416392724085

 4.54963839579024E-02

 4.54963839579024E-02

 .0429322813938

 .00249835634451

 6.57462195924E-05

 .000394477317554

 .000394477317554

 .000394477317554

 5.917159763306E-04

 4.38308130616E-05

 4.38308130616E-05

 .000262984878369

 .000262984878369

 .0002849002849

 .0002849002849

 8.766162612326E-04

 .000219154065308

 0

 0

 .000219154065308

 .000219154065308

 1.972386587776E-04

 1.972386587776E-04

 0

 0

 .000153407845716

 4.38308130616E-05

 .000460223537147

 .000460223537147

 .000460223537147

 4.163927240848E-04

 0

 0

 0

 0

 4.163927240848E-04

 0

 0

 6.57462195924E-05

 6.57462195924E-05

 .0002849002849

 .0002849002849

 6.57462195924E-05

 6.57462195924E-05

 0

 0

 0

 0

 0

 0

 0

 0

 .000394477317554

 .000394477317554

 .000394477317554

 .000394477317554

 7.232084155164E-04

 .000416392724085

 .000416392724085

 .000416392724085

 6.57462195924E-05

 6.57462195924E-05

 6.57462195924E-05

 .000241069471839

 .000241069471839

 .000241069471839

 .297260574183653

 0

 0

 0

 0

 4.56059609905712E-02

 0

 0

 0

 0

 .005500767039224

 .00335305719921

 .00335305719921

 .000109577032654

 .000109577032654

 0

 0

 .00203813280736

 .00203813280736

 .003637957484111

 0

 0

 .00306815691431

 0

 0

 .00306815691431

 0

 0

 .000569800569801

 .000569800569801

 3.64672364672362E-02

 .00186280955512

 .00186280955512

 .000635546789393

 .000635546789393

 .0338812184966

 .0338812184966

 8.76616261232E-05

 8.76616261232E-05

 .21385053692752

 0

 0

 0

 .000525969756739

 .000525969756739

 .000525969756739

 0

 0

 0

 0

 .0025641025641

 .0025641025641

 .0025641025641

 4.96164803857356E-02

 .007692307692303

 0

 .000635546789393

 .00254218715757

 .00422967346044

 .0002849002849

 0

 0

 0

 0

 .0347140039448

 .0347140039448

 7.0786763094476E-03

 0

 .0017094017094

 .000986193293886

 .0043392504931

 4.38308130616E-05

 0

 0

 0

 0

 .000131492439185

 .000131492439185

 0

 0

 .067784352399733

 .000920447074293

 .000920447074293

 .0262108262108

 .0262108262108

 .001161516546131

 .000898531667762

 .000262984878369

 .032434801665593

 .000350646504493

 .0320841551611

 0

 0

 0

 0

 0

 .000613631382862

 .000613631382862

 .000175323252246

 0

 .000175323252246

 .006267806267808

 .0057637519176

 .000504054350208

 0

 .000109577032654

 .000109577032654

 4.38308130616E-05

 0

 0

 6.57462195924E-05

 0

 .019132149901385

 0

 0

 0

 0

 0

 0

 0

 0

 2.1915406530808E-03

 0

 0

 0

 0

 .00216962524655

 2.19154065308E-05

 0

 0

 0

 0

 0

 0

 1.2053473591936E-03

 .000109577032654

 .000745123822047

 0

 4.38308130616E-05

 0

 .000306815691431

 0

 1.13302651764256E-02

 .00322156476003

 0

 .000131492439185

 .000131492439185

 0

 4.38308130616E-05

 2.19154065308E-05

 0

 0

 .0057637519176

 0

 .000525969756739

 .00109577032654

 0

 0

 0

 4.38308130616E-05

 0

 0

 0

 4.38308130616E-05

 0

 .000306815691431

 .004404996712685

 0

 .00054788516327

 .000679377602455

 .00317773394696

 0

 0

 0

 0

 0

 0

 0

 0

 .000854700854701

 .000854700854701

 .000854700854701

 0

 0

 0

 0

 0

 .034560596099063

 .010300241069477

 .000350646504493

 0

 0

 .000109577032654

 .00984001753233

 .024260355029586

 .0232741617357

 0

 0

 .000986193293886

 0

 .00451457374534

 .00451457374534

 0

 .00151216305062

 0

 0

 0

 .00300241069472

 0

 3.05281612974308E-02

 3.05281612974308E-02

 0

 2.19154065308E-05

 .0305062458909

 3.6598728906384E-03

 3.6598728906384E-03

 6.57462195924E-05

 .000175323252246

 .0034188034188

 0

 0

 0

 0

 0

 0

 0

 0

 0

 0

 .037804076265562

 1.37190444882636E-02

 0

 0

 1.37190444882636E-02

 .0116809116809

 4.38308130616E-05

 .00111768573307

 .000876616261232

 2.40850317772984E-02

 .00107385492001

 .00107385492001

 1.41135218057924E-02

 .0140477755862

 6.57462195924E-05

 .008897655051496

 .0002849002849

 .00843743151435

 0

 0

 .000175323252246

 .000920447074293

 .000394477317554

 .000394477317554

 .000219154065308

 .000219154065308

 .000175323252246

 .000175323252246

 .000525969756739

 .000525969756739

 .000525969756739

 .000525969756739

 0

 0

 0

 0

 0

 0

 .084374315143515

 .084155161078207

 .000372561911023

 0

 0

 0

 0

 .000372561911023

 0

 0

 .000372561911023

 0

 0

 1.22068814376942E-02

 0

 0

 1.22068814376942E-02

 0

 4.38308130616E-05

 2.19154065308E-05

 0

 0

 0

 .000350646504493

 2.19154065308E-05

 0

 0

 .000219154065308

 0

 0

 .000810870041639

 .000591715976331

 .0101468332238

 .002783256629406

 0

 0

 .000153407845716

 .000153407845716

 .00083278544817

 0

 .00083278544817

 .00179706333552

 .00179706333552

 0

 2.41288625903598E-02

 .000416392724085

 .000416392724085

 .019307473153585

 .000416392724085

 .0188910804295

 .000482138943677

 .000482138943677

 3.9228577690128E-03

 0

 .00357221126452

 2.19154065308E-05

 .000328731097962

 .044663598509724

 .000131492439185

 .000131492439185

 .000504054350208

 .000504054350208

 .044028051720331

 .000482138943677

 .000175323252246

 .0431514354591

 .000219154065308

 0

 0

 0

 .000219154065308

 .000219154065308

 .000219154065308

 .000219154065308

 0

 0

 0

 0

 0

 0

 0

 0

 6.8156914310776E-03

 0

 0

 0

 0

 .000591715976331

 .000591715976331

 .000591715976331

 .000591715976331

 0

 0

 0

 0

 0

 0

 0

 0

 0

 0

 .0022792022792

 0

 0

 0

 0

 0

 .0022792022792

 .00144641683103

 0

 .00144641683103

 .00083278544817

 .00083278544817

 .000613631382862

 .000613631382862

 .000613631382862

 .000613631382862

 0

 0

 0

 0

 .000131492439185

 0

 0

 0

 .000131492439185

 .000131492439185

 .000131492439185

 .000219154065308

 .000219154065308

 .000219154065308

 .000219154065308

 2.9804952881916E-03

 4.38308130616E-05

 4.38308130616E-05

 4.38308130616E-05

 .00293666447513

 .00293666447513

 .00293666447513

 .000284900284901

 .000131492439185

 .000131492439185

 .000131492439185

 .000131492439185

 0

 0

 0

 0

 0

 0

 0

 0

 0

 .000153407845716

 0

 0

 0

 .000153407845716

 .000153407845716

 .000153407845716

 0

 0

 0

 0

 0

 0

 0

 .000241069471839

 .000241069471839

 .000241069471839

 0

 0

 .000241069471839

 .000241069471839

 0

 0

 0

 0

 0

 0

 0

 0

 1.972386587774E-04

 6.57462195924E-05

 6.57462195924E-05

 6.57462195924E-05

 6.57462195924E-05

 .000131492439185

 .000131492439185

 .000131492439185

 .000131492439185

 1.46175761560342E-02

 1.41573526188872E-02

 7.2101687486302E-03

 0

 0

 0

 0

 .00221345605961

 0

 .00221345605961

 0

 0

 0

 .000197238658777

 .000197238658777

 0

 0

 8.76616261232E-05

 8.76616261232E-05

 0

 0

 0

 0

 0

 0

 0

 0

 0

 0

 0

 0

 0

 0

 0

 0

 .00471181240412

 0

 0

 .00471181240412

 0

 0

 .006947183870257

 0

 0

 .000504054350208

 .000504054350208

 0

 0

 0

 0

 .000153407845716

 0

 .000153407845716

 .000394477317554

 .000394477317554

 .000241069471839

 .000241069471839

 .00565417488494

 .00203813280736

 .00361604207758

 .000460223537147

 .000460223537147

 0

 0

 0

 0

 0

 0

 0

 0

 .000153407845716

 0

 .000153407845716

 0

 0

 0

 0

 0

 0

 .000306815691431

 .000131492439185

 0

 .000175323252246

 0

 0

 0

 0

 0

 0

 0

 0

 0

 0

 0

 0

 0

 0

 0

 0

 0

 0

 0

 0

 0

 0

 0

 0

 0

 0

 0

 0

 0

 0

 0

 0

 0

 0

 .015538023230347

 .015538023230347

 .015538023230347

 .015538023230347

 .000460223537147

 .0150777996932

 0

 0

 0

 0

 0

 0

 0

 0

 0

 0

 0

 0

 0

 0

 1.4683322375638E-03

 5.698005698006E-04

 .000175323252246

 .000175323252246

 .000175323252246

 0

 0

 0

 3.944773175546E-04

 4.38308130616E-05

 4.38308130616E-05

 .000350646504493

 .000350646504493

 8.985316677632E-04

 8.985316677632E-04

 .000131492439185

 .000131492439185

 0

 0

 0

 0

 0

 .000153407845716

 .000153407845716

 8.76616261232E-05

 8.76616261232E-05

 .000525969756739

 .000525969756739

 0

 0

 0

 0

 0

 0

 3.68617137847926E-02

 7.2978303747564E-03

 5.259697567394E-04

 5.259697567394E-04

 6.57462195924E-05

 .000460223537147

 .006771860618017

 .000350646504493

 .000350646504493

 .000306815691431

 .000306815691431

 .006114398422093

 .000306815691431

 .0054788516327

 .000328731097962

 0

 0

 0

 0

 2.95638834100362E-02

 .000810870041639

 .000810870041639

 0

 .000810870041639

 .027240850317774

 .027240850317774

 .00607056760903

 .00385711154942

 .000197238658777

 .000460223537147

 .00716633793557

 .00948937102783

 .0014245014245

 .0014245014245

 0

 .0014245014245

 8.76616261232E-05

 0

 0

 8.76616261232E-05

 0

 8.76616261232E-05

 .406903353057088

 .189173789173753

 3.55029585799316E-02

 3.55029585799316E-02

 .000131492439185

 .00241069471839

 .0130615822924

 .000701293008985

 .00118343195266

 .00116151654613

 0

 4.38308130616E-05

 0

 0

 0

 0

 .00683760683761

 0

 0

 0

 0

 .00646504492658

 .00350646504493

 0

 0

 0

 .000131492439185

 .000131492439185

 .000131492439185

 .005150120534725

 .005150120534725

 .00122726276572

 0

 0

 .000416392724085

 .00149024764409

 .00201621740083

 0

 0

 0

 0

 0

 0

 0

 .012469866316005

 .012469866316005

 .000350646504493

 .000328731097962

 0

 .0104755643217

 .00103002410695

 .0002849002849

 0

 0

 0

 0

 .034188034188016

 .034188034188016

 .000175323252246

 .0328950252027

 .00111768573307

 0

 0

 0

 0

 6.57462195924E-05

 6.57462195924E-05

 6.57462195924E-05

 .001490247644094

 .001490247644094

 .000964277887355

 .000525969756739

 7.44466359850516E-02

 0

 0

 .000460223537147

 .000460223537147

 .013061582292348

 .000569800569801

 .000306815691431

 .0002849002849

 0

 .000723208415516

 0

 0

 0

 0

 .0094674556213

 .0017094017094

 .000328731097962

 .000328731097962

 4.5364891518768E-03

 .00295857988166

 2.19154065308E-05

 .00083278544817

 .000723208415516

 2.3011176857308E-03

 .0022792022792

 2.19154065308E-05

 .00054788516327

 .00054788516327

 .000219154065308

 .000219154065308

 .000350646504493

 .000350646504493

 0

 0

 0

 0

 .01790488713566

 .00383519614289

 .0135218058295

 0

 0

 0

 .000241069471839

 0

 .000306815691431

 0

 0

 0

 0

 0

 0

 .00081087004164

 .000350646504493

 0

 .000460223537147

 4.38308130616E-05

 4.38308130616E-05

 3.38812184965544E-02

 .00710059171598

 .0107823800131

 0

 .000262984878369

 .000350646504493

 4.38308130616E-05

 .00648696033311

 .0017094017094

 2.19154065308E-05

 .00712250712251

 0

 0

 0

 0

 .001599824676748

 .001599824676748

 0

 0

 0

 .000745123822047

 .000854700854701

 0

 0

 0

 0

 0

 .00103002410695

 0

 0

 0

 .00103002410695

 .00103002410695

 0

 0

 .001643655489809

 0

 0

 .000723208415516

 .000723208415516

 .000920447074293

 0

 .000920447074293

 0

 .02051282051282

 .02051282051282

 .000416392724085

 0

 0

 0

 0

 0

 .000241069471839

 .000153407845716

 0

 .00120534735919

 .0161516546132

 .00234494849879

 0

 0

 0

 0

 0

 .000942362480825

 .000679377602455

 .000679377602455

 .000153407845716

 .000153407845716

 .000109577032654

 .000109577032654

 .008086785009861

 0

 0

 0

 .000701293008985

 0

 0

 0

 .000701293008985

 .000701293008985

 0

 .000306815691431

 .000306815691431

 .000306815691431

 .006289721674337

 0

 0

 0

 0

 .000504054350208

 .000504054350208

 0

 0

 .00221345605961

 .00221345605961

 0

 0

 .000394477317554

 0

 .000394477317554

 0

 0

 .000131492439185

 0

 0

 .000131492439185

 .00304624150778

 .00304624150778

 0

 0

 0

 0

 .000306815691431

 .000306815691431

 .000306815691431

 0

 .000482138943677

 .000482138943677

 .000482138943677

 .209642778873474

 0

 0

 0

 .000197238658777

 .000197238658777

 .000197238658777

 4.38308130616E-05

 4.38308130616E-05

 4.38308130616E-05

 0

 0

 .02031558185405

 .00054788516327

 .00054788516327

 1.66118781503432E-02

 .00153407845716

 0

 4.38308130616E-05

 0

 .00175323252246

 2.19154065308E-05

 .00569800569801

 8.76616261232E-05

 0

 .00186280955512

 0

 0

 0

 0

 .0002849002849

 8.76616261232E-05

 0

 .00225728687267

 0

 0

 .0002849002849

 0

 0

 .00118343195266

 0

 2.19154065308E-05

 0

 0

 4.38308130616E-05

 0

 0

 0

 .000854700854701

 .0002849002849

 0

 .000306815691431

 0

 0

 0

 0

 0

 .000350646504493

 .000131492439185

 .000219154065308

 4.38308130616E-05

 0

 4.38308130616E-05

 2.4764409379822E-03

 .00186280955512

 8.76616261232E-05

 .000175323252246

 .000350646504493

 0

 0

 0

 0

 0

 0

 .0002849002849

 .0002849002849

 0

 0

 0

 2.19154065308E-05

 0

 0

 2.19154065308E-05

 2.19154065308E-05

 3.068156914312E-04

 3.068156914312E-04

 8.76616261232E-05

 0

 .000219154065308

 4.17050186280686E-02

 4.17050186280686E-02

 .0348235809774

 0

 0

 0

 .00252027175104

 2.19154065308E-05

 .00129300898532

 0

 .000504054350208

 0

 2.19154065308E-05

 .0017094017094

 0

 0

 .000525969756739

 0

 0

 8.76616261232E-05

 .000175323252246

 2.19154065308E-05

 .00486522024984

 .00486522024984

 .00486522024984

 0

 0

 0

 0

 .0017094017094

 .0017094017094

 .0017094017094

 2.19154065308E-05

 2.19154065308E-05

 2.19154065308E-05

 4.38308130616E-05

 4.38308130616E-05

 4.38308130616E-05

 0

 0

 4.38308130616E-05

 4.38308130616E-05

 4.38308130616E-05

 8.76616261232E-05

 8.76616261232E-05

 8.76616261232E-05

 .047578347578323

 .000372561911023

 .000372561911023

 0

 0

 0

 0

 0

 .0472057856673

 .0472057856673

 0

 0

 0

 .0002849002849

 .0002849002849

 0

 .0002849002849

 .000241069471839

 .000241069471839

 .000241069471839

 0

 0

 0

 0

 0

 0

 6.57462195924E-05

 6.57462195924E-05

 6.57462195924E-05

 .09202279202276

 .069822485207099

 .00111768573307

 .0285338593031

 .0149243918475

 .000262984878369

 .00464606618453

 .00322156476003

 .0171159325005

 .022200306815661

 .000175323252246

 .000701293008985

 0

 0

 .0175980714442

 .00372561911023

 0

 0

 8.76616261232E-05

 8.76616261232E-05

 8.76616261232E-05

 .001008108700417

 .001008108700417

 .001008108700417

 .000460223537147

 .000460223537147

 .00054788516327

 .00054788516327

 0

 0

 0

 0

 0

 2.0162174008332E-03

 2.0162174008332E-03

 .000525969756739

 .000525969756739

 .000372561911023

 .000153407845716

 0

 0

 0

 0

 .000219154065308

 .000219154065308

 .000219154065308

 .000131492439185

 .000131492439185

 .000131492439185

 0

 0

 0

 0

 7.232084155162E-04

 7.232084155162E-04

 .000525969756739

 4.38308130616E-05

 .000109577032654

 4.38308130616E-05

 .0002849002849

 .0002849002849

 .0002849002849

 .000131492439185

 .000131492439185

 .000131492439185

 0

 0

 0

 0

 0

 0

 0

 0

 0

 .001709401709402

 .000986193293886

 .000986193293886

 .000986193293886

 .000986193293886

 .000723208415516

 .000723208415516

 .000723208415516

 .000723208415516
