# Supplementary material for: First Insight into Microbiome Profiles of Myrmecophilous Beetles and Their Host, Red Wood Ant Formica polyctena (Hymenoptera: Formicidae)—A Case Study
Source: Insects. 2020 Feb 19;11(2):134. doi: 10.3390/insects11020134 (PMC7073670; doi:10.3390/insects11020134)
Supplement: Supplementary file 1 [file insects-11-00134-s001.zip › Supplementary Files/S2_Figure.zip!/Supplemental_Figure_S2/Supplemental_Figure_S2_DP3.html]

Javascript must be enabled to view this page.

magnitude

 .999999999999037

 .999999999999037

 .00178630972229

 .00178630972229

 .00178630972229

 .00178630972229

 0

 .00178630972229

 .051612442242579

 .051612442242579

 .041275663316279

 .041275663316279

 .0110513028152

 0

 .02105463726

 0

 .00664507216691

 .000119087314819

 0

 0

 .00240556375935

 0

 0

 0

 0

 0

 0

 .0103367789263

 .0103367789263

 .0103367789263

 0

 0

 0

 0

 0

 0

 0

 0

 0

 0

 0

 0

 0

 0

 0

 0

 0

 0

 0

 0

 0

 0

 0

 0

 0

 0

 0

 0

 0

 0

 0

 0

 0

 0

 0

 0

 0

 0

 0

 0

 0

 0

 0

 0

 0

 0

 0

 0

 0

 0

 0

 0

 0

 0

 0

 0

 0

 0

 0

 0

 0

 0

 0

 .595460391558164

 .000357261944458

 .000357261944458

 .000357261944458

 .000357261944458

 1.6910398704368E-03

 0

 0

 0

 0

 0

 0

 0

 0

 0

 0

 0

 0

 0

 .00121469061116

 0

 0

 0

 0

 0

 0

 0

 0

 .00121469061116

 .00121469061116

 4.763492592768E-04

 2.38174629638E-05

 2.38174629638E-05

 0

 0

 .000452531796313

 .000452531796313

 0

 0

 .588600962224573

 0

 0

 0

 .00221502405564

 .00221502405564

 .00221502405564

 0

 0

 0

 0

 .00838374696327

 .00838374696327

 .00838374696327

 .227504406230217

 .0596389272615

 0

 0

 .0246987090935

 .0149097318154

 .0200304863526

 4.76349259277E-05

 4.76349259277E-05

 0

 0

 .101128947744

 .101128947744

 6.57361977802358E-02

 2.38174629638E-05

 .0431572428905

 .00404896870385

 .0181250893155

 .000381079407422

 0

 0

 0

 0

 .000952698518554

 .000952698518554

 0

 0

 .01874434335255

 0

 0

 .00092888105559

 .00092888105559

 0

 0

 0

 .00766922307436

 0

 .00766922307436

 0

 0

 0

 0

 0

 .0101462392226

 .0101462392226

 0

 0

 0

 0

 0

 0

 0

 .01652931929688

 .01652931929688

 0

 0

 0

 .0135521364264

 .00297718287048

 9.53174867812942E-02

 0

 0

 0

 0

 0

 0

 0

 0

 1.58624303339311E-02

 0

 0

 .000333444481494

 0

 .0144333825561

 0

 .000952698518554

 4.76349259277E-05

 9.52698518554E-05

 2.38174629638E-05

 0

 2.38174629638E-05

 0

 0

 0

 0

 0

 0

 0

 0

 6.57600152431536E-02

 .025055971038

 0

 .000119087314819

 .000142904777783

 .000119087314819

 0

 0

 .000333444481494

 2.38174629638E-05

 .0217929786119

 0

 .00304863525937

 .0132186919449

 9.52698518554E-05

 0

 0

 9.52698518554E-05

 7.14523888915E-05

 2.38174629638E-05

 4.76349259277E-05

 .000190539703711

 0

 .0013814128519

 1.36712237412457E-02

 0

 .000238174629638

 .0042156909446

 .00624017529653

 0

 4.76349259277E-05

 .00292954794455

 0

 0

 0

 0

 0

 .000785976277806

 .000785976277806

 0

 .000238174629638

 .000547801648168

 0

 0

 0

 9.74134235220906E-02

 8.097937407706E-04

 2.38174629638E-05

 .000547801648168

 0

 2.38174629638E-05

 .000214357166675

 .09660362978132

 .0947696851331

 0

 0

 .00183394464822

 0

 1.52431762968538E-02

 1.52431762968538E-02

 0

 .00488257990759

 2.38174629638E-05

 0

 0

 .0103367789263

 0

 .103201067021928

 .103201067021928

 2.38174629638E-05

 2.38174629638E-05

 .103153432096

 .003262992426043

 .003262992426043

 .000142904777783

 0

 .00312008764826

 0

 0

 0

 0

 0

 0

 0

 0

 0

 0

 4.8111275186968E-03

 .000142904777783

 0

 0

 .000142904777783

 .000142904777783

 0

 0

 0

 4.6682227409138E-03

 0

 0

 3.9298813890338E-03

 .00390606392607

 2.38174629638E-05

 .00073834135188

 .000381079407422

 .000357261944458

 0

 0

 0

 .000166722240747

 .000166722240747

 .000166722240747

 0

 0

 .000166722240747

 .000166722240747

 0

 0

 0

 0

 0

 0

 0

 0

 0

 0

 1.75534702043538E-02

 1.75534702043538E-02

 .00523984185205

 0

 0

 0

 0

 .00523984185205

 0

 .00523984185205

 0

 0

 0

 .006430715000232

 0

 0

 .006430715000232

 0

 .00312008764826

 0

 0

 0

 0

 0

 0

 0

 0

 0

 0

 0

 .000571619111132

 0

 .00273900824084

 .000285809555566

 0

 0

 .000285809555566

 .000285809555566

 0

 0

 0

 0

 0

 0

 7.145238889158E-04

 0

 0

 .000690706425952

 0

 .000690706425952

 0

 0

 2.38174629638E-05

 0

 2.38174629638E-05

 0

 0

 .00488257990759

 0

 0

 0

 0

 .00488257990759

 0

 .00295336540752

 0

 .00192921450007

 0

 0

 0

 0

 0

 0

 0

 0

 0

 0

 0

 0

 0

 0

 0

 .00204830181489

 0

 0

 0

 0

 0

 0

 0

 0

 0

 0

 0

 0

 0

 0

 0

 0

 0

 0

 .00204830181489

 0

 0

 0

 0

 0

 .00204830181489

 .00204830181489

 0

 .00204830181489

 0

 0

 0

 0

 0

 0

 0

 0

 0

 0

 0

 0

 0

 0

 0

 0

 0

 0

 0

 0

 0

 0

 0

 0

 0

 0

 0

 0

 0

 0

 0

 0

 0

 0

 0

 0

 0

 0

 0

 0

 0

 0

 0

 0

 0

 0

 0

 0

 0

 0

 0

 0

 0

 0

 0

 0

 .00547801648168

 .00547801648168

 .00547801648168

 0

 0

 0

 0

 .00547801648168

 .0013814128519

 .00409660362978

 0

 0

 0

 0

 0

 0

 0

 0

 0

 0

 0

 0

 0

 0

 7.22860000953183E-02

 6.16634116134453E-02

 2.53894155194915E-02

 2.38174629638E-05

 2.38174629638E-05

 0

 0

 0

 0

 0

 0

 0

 0

 4.76349259277E-05

 4.76349259277E-05

 0

 0

 .00176249225932

 .00176249225932

 0

 .00323917496308

 0

 0

 0

 .00323917496308

 0

 0

 0

 0

 0

 0

 0

 0

 0

 0

 .0203162959082

 0

 0

 .0203162959082

 0

 0

 3.62739960939538E-02

 0

 0

 0

 0

 0

 0

 0

 0

 2.38174629638E-05

 0

 2.38174629638E-05

 .0223407802601

 .0223407802601

 .00323917496308

 .00323917496308

 .01067022340781

 .00531129424094

 .00535892916687

 .010360596389271

 .010360596389271

 0

 0

 0

 0

 0

 0

 0

 0

 .003524984518651

 .00302481779641

 0

 0

 .000500166722241

 0

 .00683561187062

 .00516838946315

 .00166722240747

 0

 0

 0

 0

 0

 0

 0

 0

 0

 0

 0

 0

 0

 0

 0

 0

 0

 0

 0

 0

 0

 0

 0

 .000261992092602

 .000261992092602

 .000261992092602

 .000261992092602

 0

 0

 0

 0

 .00271519077788

 .00271519077788

 .00271519077788

 .00271519077788

 .00271519077788

 0

 0

 .00109560329634

 .00109560329634

 .00109560329634

 .00109560329634

 0

 .00109560329634

 0

 0

 0

 0

 0

 0

 0

 0

 0

 0

 0

 0

 0

 0

 .002524651074167

 0

 0

 0

 0

 0

 0

 0

 0

 0

 0

 0

 0

 .002524651074167

 .002524651074167

 0

 0

 .002262658981565

 .00204830181489

 .000214357166675

 0

 0

 0

 0

 0

 0

 .000261992092602

 .000261992092602

 0

 0

 0

 0

 0

 0

 .04008479016818

 0

 0

 0

 0

 0

 0

 0

 0

 0

 0

 0

 0

 0

 0

 0

 0

 0

 0

 .04008479016818

 0

 0

 0

 0

 .04008479016818

 .04008479016818

 .00145286524079

 .003953698852

 .00126232553708

 .00466822274091

 .017791644834

 .0109560329634

 0

 0

 0

 0

 0

 0

 0

 0

 0

 0

 .205830514933509

 9.98666222074117E-02

 .012480350593064

 .012480350593064

 0

 0

 .00597818320393

 0

 .000357261944458

 0

 0

 0

 0

 0

 .000357261944458

 0

 .000976515981518

 0

 0

 0

 0

 .00240556375935

 .00240556375935

 0

 0

 0

 0

 0

 0

 0

 0

 0

 0

 0

 0

 0

 0

 0

 0

 0

 0

 0

 0

 0

 .000381079407422

 .000381079407422

 0

 0

 0

 0

 0

 .000381079407422

 0

 0

 0

 0

 .00100033344448

 .00100033344448

 0

 .00100033344448

 0

 0

 0

 0

 0

 0

 0

 0

 0

 0

 0

 0

 4.92545134092848E-02

 0

 0

 0

 0

 .007859762778073

 0

 0

 0

 0

 .00600200066689

 0

 0

 0

 0

 .00140523031487

 .000452531796313

 0

 0

 2.4770161482417E-03

 .00209593674082

 4.76349259277E-05

 .000333444481494

 0

 .00266755585195

 .00266755585195

 0

 0

 0

 0

 0

 .00314390511123

 .00314390511123

 0

 0

 0

 0

 9.9080645929631E-03

 .000166722240747

 .000261992092602

 0

 0

 4.76349259277E-05

 .00883627875959

 0

 0

 0

 0

 0

 0

 9.52698518554E-05

 .000500166722241

 .00319154003716

 0

 .00319154003716

 0

 0

 0

 .020006668889667

 .0013814128519

 .0135283189635

 0

 .000333444481494

 0

 0

 .00135759538894

 0

 .000762158814843

 .00264373838899

 0

 0

 0

 0

 1.25041680560128E-02

 1.25041680560128E-02

 0

 0

 0

 .00440623064831

 .00764540561139

 .000428714333349

 0

 2.38174629638E-05

 0

 0

 0

 0

 0

 0

 0

 0

 0

 0

 .002095936740818

 0

 0

 .00154813509265

 .00154813509265

 .000547801648168

 0

 .000547801648168

 0

 2.15786214451981E-02

 2.15786214451981E-02

 4.76349259277E-05

 0

 0

 .000714523888915

 0

 0

 4.76349259277E-05

 0

 0

 0

 .0207211927785

 4.76349259277E-05

 0

 0

 .000571619111132

 .000571619111132

 .000571619111132

 0

 0

 0

 0

 0

 0

 0

 5.9067308150415E-03

 0

 0

 0

 0

 0

 0

 0

 0

 0

 0

 0

 0

 0

 5.9067308150415E-03

 0

 0

 0

 0

 0

 0

 0

 0

 0

 0

 0

 0

 .0017148573334

 0

 .0017148573334

 0

 0

 7.14523888915E-05

 0

 7.14523888915E-05

 0

 .00412042109275

 .00412042109275

 0

 0

 0

 0

 0

 0

 0

 0

 0

 0

 0

 .100057161911056

 .00204830181489

 .00204830181489

 .00204830181489

 0

 0

 0

 0

 0

 0

 0

 0

 4.52293621683244E-02

 0

 0

 4.52055447053606E-02

 .0050731196113

 0

 0

 0

 .00100033344448

 .000119087314819

 .01052731863

 2.38174629638E-05

 4.76349259277E-05

 .0146239222598

 0

 0

 0

 0

 0

 4.76349259277E-05

 0

 .00650216738913

 0

 0

 0

 9.52698518554E-05

 .00419187348164

 0

 0

 .000476349259277

 0

 0

 0

 0

 0

 0

 0

 0

 0

 .00247701614824

 0

 0

 0

 0

 0

 0

 0

 0

 0

 0

 0

 0

 0

 0

 0

 0

 0

 2.38174629638E-05

 0

 2.38174629638E-05

 0

 0

 0

 0

 0

 0

 0

 0

 0

 0

 0

 0

 .00121469061116

 .00121469061116

 .00121469061116

 0

 0

 7.5501357595338E-03

 7.5501357595338E-03

 .00254846853713

 0

 0

 0

 .00235792883342

 0

 .00261992092602

 0

 2.38174629638E-05

 0

 0

 0

 0

 0

 0

 0

 0

 0

 0

 0

 0

 0

 0

 0

 0

 0

 0

 0

 0

 0

 .000333444481494

 .000333444481494

 .000333444481494

 0

 0

 0

 0

 0

 0

 0

 0

 .00240556375935

 .00240556375935

 .00240556375935

 1.05511360929777E-02

 9.0982708521877E-03

 .00905063592626

 0

 4.76349259277E-05

 0

 0

 0

 .00145286524079

 .00145286524079

 0

 0

 0

 0

 0

 0

 0

 0

 0

 0

 0

 0

 0

 0

 0

 0

 0

 0

 0

 3.07245272233258E-02

 2.80093364454438E-02

 .00240556375935

 .015124088982

 .00600200066689

 2.38174629638E-05

 0

 0

 .00445386557424

 .002715190777882

 0

 0

 .000261992092602

 0

 0

 .00245319868528

 0

 0

 0

 0

 0

 0

 0

 0

 0

 0

 0

 0

 0

 0

 0

 0

 0

 .001357595388939

 .001357595388939

 .000571619111132

 0

 0

 0

 0

 0

 .000571619111132

 .000571619111132

 0

 0

 0

 .000785976277807

 .000785976277807

 0

 .000785976277807

 0

 0

 0

 0

 0

 0

 0

 0

 0

 0

 0

 0

 0

 0

 0

 0

 0

 0

 0

 0

 0

 0

 0

 0

 0

 0

 0

 0

 0

 0

 0

 0

 0
