# Supplementary material for: First Insight into Microbiome Profiles of Myrmecophilous Beetles and Their Host, Red Wood Ant Formica polyctena (Hymenoptera: Formicidae)—A Case Study
Source: Insects. 2020 Feb 19;11(2):134. doi: 10.3390/insects11020134 (PMC7073670; doi:10.3390/insects11020134)
Supplement: Supplementary file 1 [file insects-11-00134-s001.zip › Supplementary Files/S2_Figure.zip!/Supplemental_Figure_S2/Supplemental_Figure_S2_FPD1.html]

Javascript must be enabled to view this page.

magnitude

 .999999999999568

 .999999999999568

 0

 0

 0

 0

 0

 0

 8.51926632078E-06

 8.51926632078E-06

 8.51926632078E-06

 8.51926632078E-06

 0

 0

 0

 0

 0

 0

 0

 8.51926632078E-06

 0

 0

 0

 0

 0

 0

 0

 0

 0

 0

 0

 0

 0

 0

 0

 0

 0

 0

 0

 0

 0

 0

 0

 0

 0

 0

 0

 0

 0

 0

 0

 0

 0

 0

 0

 0

 0

 0

 0

 0

 0

 0

 0

 0

 0

 0

 0

 0

 0

 0

 0

 0

 0

 0

 0

 0

 0

 0

 0

 0

 0

 0

 0

 0

 0

 0

 0

 0

 0

 0

 0

 0

 0

 2.54726062991414E-03

 0

 0

 0

 0

 1.70385326416E-05

 0

 0

 0

 0

 1.70385326416E-05

 1.70385326416E-05

 1.70385326416E-05

 0

 0

 0

 0

 0

 0

 0

 0

 0

 0

 0

 0

 0

 0

 0

 0

 0

 0

 0

 0

 0

 0

 0

 0

 0

 0

 2.4876257656686E-03

 0

 0

 0

 8.51926632078E-06

 8.51926632078E-06

 8.51926632078E-06

 0

 8.51926632078E-06

 8.51926632078E-06

 8.51926632078E-06

 0

 0

 0

 2.03610465066708E-03

 3.407706528318E-05

 0

 0

 8.51926632078E-06

 2.55577989624E-05

 0

 0

 0

 0

 0

 .000204462391699

 .000204462391699

 2.215009243399E-04

 0

 3.40770652831E-05

 8.51926632078E-05

 .000102231195849

 0

 0

 0

 .000673022039342

 .000673022039342

 .000306693587548

 .000306693587548

 .000596348642455

 .000596348642455

 4.25963316039E-05

 0

 0

 4.25963316039E-05

 4.25963316039E-05

 0

 0

 0

 0

 0

 0

 0

 0

 0

 0

 0

 0

 0

 0

 0

 0

 0

 0

 0

 0

 0

 0

 0

 0

 0

 0

 0

 7.667339688706E-05

 0

 0

 0

 0

 0

 0

 0

 0

 8.51926632078E-06

 0

 0

 0

 0

 8.51926632078E-06

 0

 0

 0

 0

 0

 0

 0

 0

 0

 0

 0

 0

 0

 0

 0

 2.55577989624E-05

 0

 0

 0

 0

 0

 0

 0

 2.55577989624E-05

 0

 0

 0

 0

 0

 0

 0

 0

 0

 0

 0

 0

 0

 0

 0

 4.259633160388E-05

 0

 0

 8.51926632078E-06

 0

 0

 0

 3.40770652831E-05

 0

 0

 0

 0

 0

 0

 0

 0

 0

 0

 0

 0

 0

 0

 0

 0

 0

 0

 0

 0

 0

 0

 0

 0

 0

 0

 .000042596331604

 .000042596331604

 0

 2.55577989624E-05

 0

 0

 0

 1.70385326416E-05

 0

 .000272616522265

 .000272616522265

 0

 0

 .000272616522265

 0

 0

 0

 0

 0

 0

 0

 0

 0

 0

 0

 0

 0

 0

 0

 4.259633160394E-05

 2.555779896238E-05

 0

 0

 2.555779896238E-05

 8.51926632078E-06

 0

 0

 1.70385326416E-05

 1.703853264156E-05

 0

 0

 8.51926632078E-06

 8.51926632078E-06

 0

 8.51926632078E-06

 0

 0

 8.51926632078E-06

 0

 0

 0

 0

 0

 0

 0

 0

 0

 0

 0

 0

 0

 0

 0

 0

 0

 0

 0

 1.70385326416E-05

 1.70385326416E-05

 0

 0

 0

 0

 0

 0

 0

 0

 0

 0

 0

 1.70385326416E-05

 0

 0

 1.70385326416E-05

 0

 1.70385326416E-05

 0

 0

 0

 0

 0

 0

 0

 0

 0

 0

 0

 0

 0

 0

 0

 0

 0

 0

 0

 0

 0

 0

 0

 0

 0

 0

 0

 0

 0

 0

 0

 0

 0

 0

 0

 0

 0

 0

 0

 0

 0

 0

 0

 0

 0

 0

 0

 0

 0

 0

 0

 0

 0

 0

 0

 0

 0

 0

 0

 0

 0

 0

 0

 0

 0

 0

 0

 0

 0

 0

 0

 0

 0

 0

 0

 0

 0

 0

 0

 0

 0

 0

 0

 0

 0

 0

 0

 0

 0

 0

 0

 0

 0

 0

 0

 0

 0

 0

 0

 0

 0

 0

 0

 0

 0

 0

 0

 0

 0

 0

 0

 0

 0

 0

 0

 0

 0

 0

 0

 0

 0

 0

 0

 0

 0

 0

 0

 0

 0

 0

 0

 0

 0

 0

 0

 0

 0

 0

 0

 0

 0

 0

 0

 0

 0

 0

 0

 0

 0

 0

 0

 0

 0

 0

 0

 0

 0

 0

 0

 0

 0

 0

 0

 0

 0

 0

 0

 0

 0

 0

 0

 0

 5.16267539039988E-02

 5.15841575723949E-02

 1.107504621702E-04

 0

 0

 0

 0

 4.25963316039E-05

 0

 4.25963316039E-05

 0

 0

 0

 0

 0

 0

 0

 0

 0

 0

 0

 0

 0

 0

 0

 0

 0

 0

 0

 0

 0

 0

 0

 0

 0

 6.81541305663E-05

 0

 0

 6.81541305663E-05

 0

 0

 5.14734071102247E-02

 0

 0

 0

 0

 0

 0

 0

 0

 0

 0

 0

 .0514222915123

 .0514222915123

 0

 0

 5.11155979247E-05

 0

 5.11155979247E-05

 4.25963316039E-05

 4.25963316039E-05

 0

 0

 0

 0

 0

 0

 0

 0

 0

 0

 0

 0

 0

 0

 0

 0

 0

 0

 0

 0

 0

 0

 0

 0

 4.25963316039E-05

 4.25963316039E-05

 0

 0

 0

 0

 0

 0

 0

 0

 0

 0

 0

 0

 0

 0

 0

 0

 0

 0

 0

 0

 0

 0

 0

 0

 0

 0

 0

 0

 0

 1.70385326416E-05

 1.70385326416E-05

 1.70385326416E-05

 1.70385326416E-05

 1.70385326416E-05

 0

 0

 0

 0

 0

 0

 0

 0

 0

 0

 0

 0

 0

 0

 0

 0

 0

 0

 0

 0

 0

 0

 0

 0

 0

 0

 0

 0

 0

 0

 0

 0

 0

 0

 0

 0

 0

 0

 0

 0

 0

 0

 0

 0

 0

 0

 0

 0

 0

 0

 0

 0

 0

 0

 0

 0

 0

 0

 0

 0

 0

 0

 0

 0

 0

 0

 0

 0

 0

 0

 0

 0

 0

 0

 0

 0

 0

 0

 0

 0

 0

 0

 0

 0

 0

 0

 0

 0

 0

 0

 0

 .94577486986773

 .945408541415937

 .0693212700522

 .0693212700522

 0

 0

 0

 0

 0

 0

 0

 0

 0

 0

 0

 0

 0

 0

 0

 0

 0

 .0693212700522

 0

 0

 0

 0

 0

 0

 0

 0

 0

 0

 0

 0

 0

 0

 0

 0

 0

 0

 0

 0

 0

 0

 0

 0

 0

 0

 0

 0

 0

 0

 0

 0

 0

 0

 0

 0

 0

 0

 0

 0

 0

 0

 0

 0

 0

 0

 0

 0

 0

 0

 5.111559792468E-05

 0

 0

 0

 0

 8.51926632078E-06

 0

 0

 0

 0

 0

 0

 8.51926632078E-06

 0

 0

 0

 0

 0

 0

 0

 0

 0

 0

 0

 0

 0

 0

 0

 0

 0

 0

 8.51926632078E-06

 8.51926632078E-06

 0

 0

 0

 0

 1.703853264156E-05

 0

 8.51926632078E-06

 0

 0

 0

 0

 0

 0

 0

 0

 0

 0

 8.51926632078E-06

 0

 0

 0

 0

 0

 0

 0

 1.703853264156E-05

 8.51926632078E-06

 0

 0

 0

 0

 0

 0

 0

 0

 8.51926632078E-06

 0

 0

 0

 0

 1.70385326416E-05

 1.70385326416E-05

 0

 0

 0

 1.70385326416E-05

 0

 0

 0

 0

 0

 0

 0

 0

 0

 0

 0

 0

 0

 0

 .875942443836283

 0

 0

 .875908366771

 .875908366771

 3.40770652831E-05

 0

 3.40770652831E-05

 0

 7.66733968871E-05

 7.66733968871E-05

 0

 0

 0

 0

 0

 0

 0

 0

 0

 0

 7.66733968871E-05

 0

 0

 0

 0

 0

 0

 0

 0

 0

 0

 0

 0

 0

 8.51926632078E-06

 0

 0

 0

 0

 0

 0

 0

 0

 0

 0

 0

 0

 0

 0

 0

 0

 0

 0

 0

 0

 0

 0

 0

 0

 0

 0

 0

 0

 0

 0

 0

 0

 0

 0

 0

 0

 0

 0

 0

 0

 0

 8.51926632078E-06

 8.51926632078E-06

 8.51926632078E-06

 0

 0

 0

 0

 3.5780918547328E-04

 0

 0

 0

 0

 0

 0

 0

 0

 0

 0

 0

 5.111559792478E-05

 0

 0

 2.555779896238E-05

 8.51926632078E-06

 0

 0

 0

 0

 0

 0

 0

 0

 0

 0

 0

 0

 0

 0

 0

 0

 0

 0

 0

 0

 0

 0

 0

 0

 1.70385326416E-05

 0

 0

 0

 0

 0

 0

 0

 0

 0

 0

 0

 0

 0

 0

 0

 0

 0

 0

 2.55577989624E-05

 2.55577989624E-05

 0

 0

 0

 0

 0

 0

 0

 0

 0

 0

 0

 0

 0

 0

 0

 0

 0

 1.70385326416E-05

 1.70385326416E-05

 1.70385326416E-05

 0

 0

 2.55577989624E-05

 2.55577989624E-05

 0

 2.55577989624E-05

 0

 1.703853264156E-05

 1.703853264156E-05

 8.51926632078E-06

 0

 0

 0

 0

 0

 0

 0

 0

 0

 0

 0

 0

 0

 8.51926632078E-06

 0

 0

 0

 0

 0

 0

 0

 0

 0

 0

 0

 0

 0

 0

 0

 0

 0

 0

 0

 0

 0

 0

 0

 8.51926632078E-06

 8.51926632078E-06

 8.51926632078E-06

 0

 0

 0

 2.215009243406E-04

 1.70385326416E-05

 1.70385326416E-05

 0

 0

 0

 0

 0

 .000204462391699

 .000204462391699

 0

 0

 0

 0

 0

 0

 0

 0

 0

 0

 0

 0

 0

 8.51926632078E-06

 8.51926632078E-06

 8.51926632078E-06

 0

 0

 0

 8.51926632078E-06

 8.51926632078E-06

 0

 8.51926632078E-06

 0

 0

 0

 0

 0

 0

 0

 0

 0

 0

 0

 0

 0

 0

 0

 0

 0

 0

 0

 0

 0

 0

 0

 0

 0

 0

 0

 0

 0

 0

 0

 0

 0

 0

 0

 0

 0

 0

 0

 0

 0

 0

 0

 0

 0

 0

 0

 0

 0

 0

 0

 0

 0

 0

 0

 0

 0

 0

 0

 0

 0

 0

 0

 0

 0

 0

 0

 0

 0

 0

 8.51926632078E-06

 8.51926632078E-06

 8.51926632078E-06

 8.51926632078E-06

 8.51926632078E-06

 0

 0

 0

 0
