# Supplementary material for: First Insight into Microbiome Profiles of Myrmecophilous Beetles and Their Host, Red Wood Ant Formica polyctena (Hymenoptera: Formicidae)—A Case Study
Source: Insects. 2020 Feb 19;11(2):134. doi: 10.3390/insects11020134 (PMC7073670; doi:10.3390/insects11020134)
Supplement: Supplementary file 1 [file insects-11-00134-s001.zip › Supplementary Files/S2_Figure.zip!/Supplemental_Figure_S2/Supplemental_Figure_S2_FPD2.html]

Javascript must be enabled to view this page.

magnitude

 1.00000000000003

 1.00000000000003

 0

 0

 0

 0

 0

 0

 8.49943137607174E-03

 8.49943137607174E-03

 8.49943137607174E-03

 8.49943137607174E-03

 6.65057228174E-06

 0

 0

 0

 0

 .00429626969401

 0

 .00419651110978

 0

 0

 0

 0

 0

 0

 0

 0

 0

 0

 0

 0

 0

 0

 0

 0

 0

 0

 0

 0

 0

 0

 0

 0

 0

 0

 0

 0

 0

 0

 0

 0

 0

 0

 0

 0

 0

 0

 0

 0

 0

 0

 0

 0

 0

 0

 0

 0

 0

 0

 0

 0

 0

 0

 0

 0

 0

 0

 0

 0

 0

 0

 0

 0

 0

 0

 0

 0

 0

 0

 0

 0

 0

 .009357355200422

 0

 0

 0

 0

 0

 0

 0

 0

 0

 0

 0

 0

 0

 0

 0

 0

 0

 0

 0

 0

 0

 0

 0

 0

 0

 0

 0

 0

 0

 0

 0

 0

 0

 0

 0

 0

 0

 0

 .007175967492012

 0

 0

 0

 0

 0

 0

 0

 .00177570279923

 .00177570279923

 .00177570279923

 0

 0

 0

 .002846444936591

 .00144317418514

 0

 0

 0

 .00144317418514

 0

 0

 0

 0

 0

 .0010574409928

 .0010574409928

 0

 0

 0

 0

 0

 0

 0

 0

 0

 0

 0

 0

 .000345829758651

 .000345829758651

 0

 0

 0

 0

 0

 0

 0

 0

 0

 0

 0

 0

 0

 0

 0

 0

 0

 0

 0

 0

 0

 0

 0

 0

 0

 0

 0

 0

 0

 0

 0

 0

 .00178900394379

 0

 0

 0

 0

 0

 0

 0

 0

 0

 0

 0

 0

 0

 0

 0

 0

 0

 0

 0

 0

 0

 0

 0

 0

 0

 0

 0

 0

 0

 0

 0

 0

 0

 0

 0

 0

 0

 0

 0

 0

 0

 0

 0

 0

 0

 0

 0

 0

 0

 0

 0

 0

 0

 .00178900394379

 0

 0

 0

 0

 0

 0

 .00178900394379

 0

 0

 0

 0

 0

 0

 0

 0

 0

 0

 0

 0

 0

 .000764815812401

 0

 0

 0

 0

 0

 0

 .000764815812401

 .000764815812401

 0

 0

 0

 0

 0

 0

 0

 0

 0

 0

 0

 0

 0

 0

 0

 0

 0

 0

 0

 0

 0

 0

 0

 .00218138770841

 .00218138770841

 0

 0

 .00218138770841

 .00218138770841

 0

 0

 0

 0

 0

 0

 0

 0

 0

 0

 0

 0

 0

 0

 0

 0

 0

 0

 0

 0

 0

 0

 0

 0

 0

 0

 0

 0

 0

 0

 0

 0

 0

 0

 0

 0

 0

 0

 0

 0

 0

 0

 .000518744637976

 .000518744637976

 0

 0

 0

 0

 0

 0

 0

 0

 0

 0

 0

 0

 0

 0

 0

 0

 0

 0

 0

 0

 0

 0

 0

 0

 0

 0

 0

 0

 0

 0

 0

 0

 0

 0

 0

 0

 0

 0

 0

 0

 0

 0

 .000518744637976

 0

 0

 0

 0

 0

 0

 0

 .000518744637976

 0

 .000518744637976

 0

 0

 0

 0

 0

 0

 0

 0

 0

 0

 0

 0

 0

 0

 0

 0

 0

 0

 0

 0

 0

 0

 0

 0

 0

 0

 0

 .00158948677534

 0

 0

 0

 0

 0

 0

 0

 0

 0

 0

 0

 0

 0

 0

 0

 0

 0

 0

 .00158948677534

 0

 0

 0

 0

 0

 .00158948677534

 .00158948677534

 0

 .00158948677534

 0

 0

 0

 0

 0

 0

 0

 0

 0

 0

 0

 0

 0

 0

 0

 0

 0

 0

 0

 0

 0

 0

 0

 0

 0

 0

 0

 0

 0

 0

 0

 0

 0

 0

 0

 0

 0

 0

 0

 0

 0

 0

 0

 0

 0

 0

 0

 0

 0

 0

 0

 0

 0

 0

 0

 0

 0

 0

 0

 0

 0

 0

 0

 0

 0

 0

 0

 0

 0

 0

 0

 0

 0

 0

 0

 0

 0

 0

 0

 0

 .068208269321565

 6.08859892393605E-02

 3.59197408937205E-02

 .000026602289127

 .000026602289127

 0

 0

 2.54118366885635E-02

 0

 .025398535544

 1.33011445635E-05

 0

 0

 0

 0

 0

 0

 .00307256439417

 0

 .00307256439417

 0

 0

 0

 0

 0

 0

 0

 0

 0

 0

 0

 0

 0

 0

 0

 .00740873752186

 0

 0

 .00740873752186

 0

 0

 .02496624834564

 0

 0

 0

 0

 0

 0

 0

 0

 0

 0

 0

 .0162739503734

 .0162739503734

 .00256047032847

 .00256047032847

 .00613182764377

 0

 .00613182764377

 7.3222800822045E-03

 7.3222800822045E-03

 0

 0

 0

 0

 0

 2.3010980094835E-03

 1.33011445635E-05

 .00228779686492

 .005021182072721

 .00421646282663

 .000804719246091

 0

 0

 0

 0

 0

 0

 0

 0

 0

 0

 0

 0

 0

 0

 0

 0

 0

 0

 0

 0

 0

 0

 0

 0

 0

 0

 0

 0

 0

 0

 0

 0

 0

 0

 0

 0

 0

 0

 0

 0

 0

 0

 0

 0

 0

 0

 0

 0

 0

 0

 0

 0

 0

 0

 0

 0

 0

 0

 0

 0

 0

 0

 0

 0

 0

 0

 0

 0

 0

 0

 0

 0

 0

 0

 0

 0

 0

 0

 0

 0

 0

 0

 0

 0

 0

 0

 0

 0

 0

 0

 0

 0

 0

 0

 0

 0

 0

 0

 0

 0

 0

 0

 0

 0

 0

 0

 0

 0

 0

 0

 0

 0

 0

 0

 0

 0

 0

 0

 0

 0

 0

 0

 0

 0

 0

 0

 0

 0

 0

 0

 0

 0

 0

 0

 0

 0

 0

 0

 0

 0

 .911826712688651

 .889667005845871

 .0239420602143

 .0239420602143

 0

 0

 0

 0

 0

 0

 0

 0

 0

 0

 0

 0

 0

 0

 0

 0

 0

 .0239420602143

 0

 0

 0

 0

 0

 0

 0

 .00230109800948

 .00230109800948

 0

 0

 0

 .00230109800948

 0

 0

 0

 0

 0

 0

 0

 0

 0

 0

 0

 0

 0

 0

 0

 0

 0

 0

 0

 0

 0

 0

 0

 0

 0

 0

 0

 0

 0

 0

 0

 0

 0

 0

 0

 0

 0

 2.10025072657809E-02

 0

 0

 0

 0

 0

 0

 0

 0

 0

 0

 0

 0

 0

 0

 0

 0

 0

 0

 0

 0

 0

 0

 0

 0

 0

 0

 0

 0

 0

 0

 0

 0

 0

 0

 0

 0

 2.09360015429635E-02

 1.33011445635E-05

 .0209227003984

 0

 0

 0

 0

 0

 0

 0

 0

 0

 0

 0

 0

 0

 0

 0

 0

 0

 0

 6.650572281744E-05

 0

 5.98551505357E-05

 0

 6.65057228174E-06

 0

 0

 0

 0

 0

 0

 0

 0

 0

 0

 .002952854093095

 .002952854093095

 0

 0

 0

 .00213483370244

 .00057194921623

 0

 0

 .000246071174425

 0

 0

 0

 0

 0

 0

 0

 0

 0

 0

 .835305228014845

 0

 0

 .835285276298

 .835285276298

 1.99517168452E-05

 0

 1.99517168452E-05

 0

 .00416325824837

 .00416325824837

 0

 0

 0

 0

 0

 0

 0

 0

 0

 0

 .00416325824837

 0

 0

 0

 0

 0

 0

 0

 0

 0

 0

 0

 0

 0

 0

 0

 0

 0

 0

 0

 0

 0

 0

 0

 0

 0

 0

 0

 0

 0

 0

 0

 0

 0

 0

 0

 0

 0

 0

 0

 0

 0

 0

 0

 0

 0

 0

 0

 0

 0

 0

 0

 0

 0

 0

 0

 0

 0

 0

 0

 0

 0

 0

 .02215970684278

 0

 0

 0

 0

 0

 0

 0

 0

 0

 0

 0

 .00416990882066

 0

 0

 .00416990882066

 .00201512340137

 0

 0

 0

 0

 0

 .00215478541929

 0

 0

 0

 0

 0

 0

 0

 0

 0

 0

 0

 0

 0

 0

 0

 0

 0

 0

 0

 0

 0

 0

 0

 0

 0

 0

 0

 0

 0

 0

 0

 0

 0

 0

 0

 0

 0

 0

 0

 0

 0

 0

 0

 0

 0

 0

 0

 0

 0

 0

 0

 0

 0

 0

 0

 0

 0

 0

 0

 0

 0

 .00206832797962

 .00206832797962

 0

 .00206832797962

 0

 .00011305972879

 .00011305972879

 0

 0

 0

 0

 0

 0

 .00011305972879

 0

 0

 0

 0

 0

 0

 0

 0

 0

 0

 0

 0

 0

 0

 0

 0

 0

 0

 0

 0

 0

 0

 0

 0

 0

 0

 .00636459767363

 .00636459767363

 .00636459767363

 0

 0

 .00184220852204

 .00184220852204

 .00184220852204

 .00376422391147

 .00376422391147

 .00376422391147

 .00383738020657

 .00383738020657

 .00383738020657

 0

 0

 0

 0

 0

 0

 0

 0

 0

 0

 0

 0

 0

 0

 0

 0

 0

 0

 0

 0

 0

 0

 0

 0

 0

 0

 0

 0

 0

 0

 0

 0

 0

 0

 0

 0

 0

 0

 0

 0

 0

 0

 0

 0

 0

 0

 0

 0

 0

 0

 0

 0

 0

 0

 0

 0

 0

 0

 0

 0

 0

 0

 0

 0

 0

 0

 0

 0

 0

 0

 0

 0

 0

 0

 0

 0

 0

 0

 0

 0

 0

 0

 0

 0

 0

 0

 0

 0

 0

 0

 0

 0

 0

 0

 0

 0

 0

 0

 0

 0

 0

 0

 0

 0

 0

 0

 0

 0

 0
