# Supplementary material for: First Insight into Microbiome Profiles of Myrmecophilous Beetles and Their Host, Red Wood Ant Formica polyctena (Hymenoptera: Formicidae)—A Case Study
Source: Insects. 2020 Feb 19;11(2):134. doi: 10.3390/insects11020134 (PMC7073670; doi:10.3390/insects11020134)
Supplement: Supplementary file 1 [file insects-11-00134-s001.zip › Supplementary Files/S2_Figure.zip!/Supplemental_Figure_S2/Supplemental_Figure_S2_FPD4.html]

Javascript must be enabled to view this page.

magnitude

 .999999999999958

 .999999999999958

 0

 0

 0

 0

 0

 0

 7.557131917297E-04

 6.851799605015E-04

 6.146467292734E-04

 6.045705533837E-04

 .000191447341905

 0

 8.06094071178E-05

 0

 4.03047035589E-05

 2.01523517795E-05

 0

 .000251904397243

 2.01523517795E-05

 0

 0

 1.00761758897E-05

 1.00761758897E-05

 0

 0

 7.05332312281E-05

 7.05332312281E-05

 7.05332312281E-05

 0

 0

 0

 0

 0

 0

 0

 0

 0

 0

 0

 0

 5.03808794487E-05

 3.02285276692E-05

 3.02285276692E-05

 3.02285276692E-05

 0

 0

 2.01523517795E-05

 2.01523517795E-05

 0

 2.01523517795E-05

 0

 0

 0

 0

 0

 0

 0

 0

 0

 0

 0

 0

 0

 0

 0

 0

 0

 0

 0

 0

 0

 0

 0

 0

 0

 0

 0

 0

 0

 0

 0

 2.01523517795E-05

 2.01523517795E-05

 2.01523517795E-05

 2.01523517795E-05

 0

 0

 0

 0

 0

 0

 .015920357905777

 0

 0

 0

 0

 1.612188142358E-04

 0

 0

 0

 0

 6.04570553384E-05

 3.02285276692E-05

 3.02285276692E-05

 0

 0

 0

 0

 3.02285276692E-05

 3.02285276692E-05

 .000040304703559

 2.01523517795E-05

 2.01523517795E-05

 2.01523517795E-05

 0

 0

 2.01523517795E-05

 0

 0

 0

 0

 6.04570553384E-05

 0

 0

 0

 0

 6.04570553384E-05

 6.04570553384E-05

 0

 0

 1.46205312160019E-02

 0

 0

 0

 0

 0

 0

 0

 0

 0

 0

 0

 0

 0

 8.2926927572488E-03

 1.410664624557E-04

 1.00761758897E-05

 0

 .000130990286566

 0

 0

 0

 0

 0

 0

 .00222683487163

 .00222683487163

 8.867034782961E-04

 0

 .000100761758897

 .00038289468381

 .000332513804361

 0

 0

 7.05332312281E-05

 .00380879448632

 .00380879448632

 .000302285276692

 .000302285276692

 .000927008181855

 .000927008181855

 1.1083793478705E-03

 0

 0

 .000483656442707

 .000483656442707

 4.03047035589E-05

 0

 4.03047035589E-05

 .000453427915038

 0

 .000453427915038

 0

 3.02285276692E-05

 0

 3.02285276692E-05

 0

 6.04570553384E-05

 6.04570553384E-05

 2.01523517795E-05

 0

 2.01523517795E-05

 2.01523517795E-05

 2.01523517795E-05

 0

 0

 0

 0

 0

 0

 0

 0

 0

 8.161702470678E-04

 0

 0

 3.02285276692E-05

 3.02285276692E-05

 0

 0

 0

 0

 .000201523517795

 0

 0

 0

 0

 .000201523517795

 0

 0

 0

 0

 0

 0

 0

 7.05332312281E-05

 4.03047035589E-05

 0

 0

 0

 2.01523517795E-05

 1.00761758897E-05

 0

 .000423199387368

 8.06094071178E-05

 0

 1.00761758897E-05

 0

 0

 0

 0

 0

 0

 5.03808794486E-05

 0

 3.02285276692E-05

 .000241828221353

 0

 0

 0

 1.00761758897E-05

 0

 0

 0

 0

 0

 0

 9.06855830075E-05

 0

 5.03808794486E-05

 1.00761758897E-05

 3.02285276692E-05

 0

 0

 0

 0

 0

 0

 0

 0

 1.511426383456E-04

 1.511426383456E-04

 0

 0

 4.03047035589E-05

 0

 1.00761758897E-05

 .000100761758897

 7.355608399502E-04

 .000634799081053

 5.03808794486E-05

 .000181371166015

 0

 6.04570553384E-05

 .000342589980251

 1.007617588972E-04

 9.06855830075E-05

 0

 0

 1.00761758897E-05

 0

 3.929708596995E-04

 3.929708596995E-04

 0

 .000171294990125

 2.01523517795E-05

 0

 0

 .000201523517795

 0

 .00310346217404

 .00310346217404

 0

 0

 .00310346217404

 2.01523517795E-05

 2.01523517795E-05

 0

 0

 2.01523517795E-05

 0

 0

 0

 0

 0

 0

 0

 0

 0

 0

 1.1386078755393E-03

 1.007617588973E-04

 3.02285276692E-05

 3.02285276692E-05

 7.05332312281E-05

 1.00761758897E-05

 0

 0

 6.04570553384E-05

 .001037846116642

 2.01523517795E-05

 2.01523517795E-05

 .000231752045464

 .000231752045464

 0

 7.859417193985E-04

 2.01523517795E-05

 .000110837934787

 .000554189673935

 0

 .000100761758897

 0

 0

 0

 0

 0

 0

 0

 0

 0

 0

 0

 0

 0

 0

 0

 0

 0

 1.9043972431577E-03

 1.9043972431577E-03

 0

 0

 0

 0

 0

 0

 0

 0

 0

 0

 0

 .000624722905163

 0

 0

 .000624722905163

 0

 1.00761758897E-05

 .00035266615614

 0

 0

 0

 2.01523517795E-05

 0

 .000120914110677

 0

 0

 0

 0

 3.02285276692E-05

 2.01523517795E-05

 7.05332312281E-05

 .000241828221353

 0

 0

 .000241828221353

 .000241828221353

 0

 0

 0

 0

 0

 0

 .000513884970376

 0

 0

 .000272056749023

 0

 .000272056749023

 0

 0

 .000241828221353

 0

 .000241828221353

 0

 0

 5.239611462657E-04

 0

 0

 0

 0

 5.038087944862E-04

 0

 .000141066462456

 3.02285276692E-05

 .000332513804361

 0

 2.01523517795E-05

 2.01523517795E-05

 0

 0

 0

 0

 0

 0

 0

 0

 0

 0

 0

 0

 2.01523517795E-05

 0

 0

 0

 0

 0

 0

 0

 0

 0

 0

 0

 0

 0

 0

 0

 0

 0

 0

 2.01523517795E-05

 0

 0

 0

 0

 0

 2.01523517795E-05

 2.01523517795E-05

 2.01523517795E-05

 0

 0

 0

 0

 0

 0

 0

 0

 0

 0

 0

 0

 0

 0

 0

 0

 0

 0

 0

 0

 0

 0

 0

 0

 0

 0

 0

 0

 0

 0

 0

 0

 0

 0

 0

 0

 0

 0

 0

 0

 0

 0

 0

 0

 0

 0

 0

 0

 0

 0

 0

 0

 0

 0

 0

 0

 0

 6.04570553384E-05

 6.04570553384E-05

 6.04570553384E-05

 6.04570553384E-05

 6.04570553384E-05

 0

 0

 0

 0

 0

 0

 0

 0

 0

 0

 0

 0

 0

 0

 0

 0

 0

 0

 0

 3.19616299221652E-02

 3.18709443391576E-02

 4.030470355884E-04

 0

 0

 0

 0

 .000171294990125

 0

 .000171294990125

 0

 4.03047035589E-05

 4.03047035589E-05

 0

 0

 0

 0

 0

 0

 0

 0

 0

 0

 0

 0

 0

 0

 0

 0

 0

 0

 0

 0

 0

 0

 .000171294990125

 0

 0

 .000171294990125

 2.01523517795E-05

 2.01523517795E-05

 3.14678973035692E-02

 0

 0

 0

 0

 0

 0

 0

 0

 0

 0

 0

 .0314376687759

 .0314376687759

 0

 0

 3.02285276692E-05

 0

 3.02285276692E-05

 9.06855830076E-05

 9.06855830076E-05

 0

 0

 0

 0

 0

 0

 0

 0

 0

 0

 0

 0

 0

 0

 0

 0

 0

 0

 0

 0

 0

 0

 0

 0

 0

 0

 0

 9.06855830076E-05

 7.05332312281E-05

 0

 0

 0

 0

 2.01523517795E-05

 0

 0

 0

 0

 0

 0

 0

 0

 0

 0

 0

 0

 0

 0

 0

 0

 0

 0

 0

 0

 0

 0

 0

 0

 0

 0

 0

 0

 0

 0

 0

 0

 0

 0

 0

 0

 0

 0

 0

 0

 0

 7.05332312281E-05

 0

 0

 0

 0

 0

 0

 0

 0

 0

 0

 0

 0

 7.05332312281E-05

 7.05332312281E-05

 4.03047035589E-05

 4.03047035589E-05

 0

 0

 0

 0

 0

 0

 0

 0

 0

 3.02285276692E-05

 3.02285276692E-05

 0

 0

 0

 0

 0

 0

 3.728185079202E-04

 3.02285276692E-05

 0

 0

 0

 0

 3.02285276692E-05

 0

 0

 0

 0

 3.02285276692E-05

 2.01523517795E-05

 1.00761758897E-05

 0

 3.02285276692E-05

 3.02285276692E-05

 3.02285276692E-05

 3.02285276692E-05

 3.123614525818E-04

 8.06094071178E-05

 8.06094071178E-05

 8.06094071178E-05

 0

 2.015235177948E-04

 2.015235177948E-04

 4.03047035589E-05

 0

 0

 0

 .000120914110677

 4.03047035589E-05

 0

 0

 0

 0

 3.02285276692E-05

 2.01523517795E-05

 2.01523517795E-05

 1.00761758897E-05

 0

 1.00761758897E-05

 .948772721776627

 .943059530047146

 1.60311958405917E-02

 1.60311958405917E-02

 0

 2.01523517795E-05

 1.00761758897E-05

 0

 4.03047035589E-05

 0

 3.02285276692E-05

 0

 0

 0

 0

 .000141066462456

 1.00761758897E-05

 0

 2.01523517795E-05

 3.02285276692E-05

 0

 .0157289105639

 0

 0

 0

 0

 0

 0

 0

 1.612188142352E-04

 1.612188142352E-04

 0

 1.00761758897E-05

 0

 0

 .000130990286566

 2.01523517795E-05

 0

 0

 0

 0

 0

 0

 0

 0

 0

 0

 0

 0

 0

 0

 0

 0

 0

 0

 0

 1.914473419047E-04

 1.914473419047E-04

 1.00761758897E-05

 .000181371166015

 0

 3.02285276692E-05

 3.02285276692E-05

 0

 3.02285276692E-05

 0

 0

 0

 .000151142638346

 .000151142638346

 .000151142638346

 0

 1.3300552174437E-03

 0

 0

 0

 0

 4.231993873681E-04

 0

 8.06094071178E-05

 0

 1.00761758897E-05

 .000130990286566

 0

 3.02285276692E-05

 3.02285276692E-05

 0

 .000110837934787

 3.02285276692E-05

 0

 0

 1.410664624561E-04

 5.03808794486E-05

 0

 9.06855830075E-05

 0

 1.00761758897E-05

 0

 1.00761758897E-05

 0

 0

 0

 0

 2.01523517795E-05

 2.01523517795E-05

 0

 0

 0

 0

 1.813711660151E-04

 1.00761758897E-05

 .000151142638346

 0

 0

 0

 1.00761758897E-05

 1.00761758897E-05

 0

 0

 0

 0

 0

 0

 0

 0

 0

 0

 0

 1.00761758897E-05

 1.00761758897E-05

 5.441134980455E-04

 .000272056749023

 .000181371166015

 0

 1.00761758897E-05

 0

 0

 0

 0

 5.03808794486E-05

 3.02285276692E-05

 0

 0

 0

 0

 0

 0

 0

 0

 0

 0

 0

 0

 0

 0

 0

 0

 2.01523517795E-05

 0

 0

 0

 0

 0

 2.01523517795E-05

 2.01523517795E-05

 .92306839708189

 0

 0

 .923058320906

 .923058320906

 1.00761758897E-05

 0

 1.00761758897E-05

 0

 2.0756922332859E-03

 2.0756922332859E-03

 .000100761758897

 0

 7.05332312281E-05

 0

 0

 0

 .000120914110677

 4.03047035589E-05

 0

 4.03047035589E-05

 .0015718834388

 .000130990286566

 0

 0

 0

 0

 0

 0

 0

 0

 0

 0

 0

 0

 9.06855830077E-05

 0

 0

 0

 3.02285276692E-05

 0

 0

 0

 3.02285276692E-05

 3.02285276692E-05

 0

 0

 0

 0

 6.04570553385E-05

 0

 0

 0

 0

 0

 0

 0

 0

 2.01523517795E-05

 2.01523517795E-05

 0

 0

 0

 0

 0

 0

 0

 2.01523517795E-05

 0

 0

 2.01523517795E-05

 2.01523517795E-05

 2.01523517795E-05

 0

 0

 0

 0

 0

 0

 0

 0

 0

 0

 0

 5.6225061464729E-03

 0

 0

 0

 0

 0

 0

 0

 0

 0

 0

 0

 2.0656160573942E-03

 0

 0

 1.9850066502764E-03

 .000584418201604

 0

 0

 0

 3.02285276692E-05

 0

 .00111845552376

 0

 4.03047035589E-05

 0

 0

 0

 0

 0

 0

 0

 0

 .000110837934787

 0

 5.03808794486E-05

 0

 0

 0

 2.01523517795E-05

 0

 2.01523517795E-05

 0

 0

 0

 0

 0

 0

 0

 0

 1.00761758897E-05

 0

 1.00761758897E-05

 1.00761758897E-05

 0

 0

 0

 0

 0

 0

 0

 0

 0

 0

 0

 0

 0

 0

 0

 0

 0

 0

 0

 0

 7.05332312281E-05

 7.05332312281E-05

 0

 0

 0

 0

 0

 0

 0

 0

 0

 0

 0

 0

 0

 1.4610455040114E-03

 1.4610455040114E-03

 .00112853169965

 0

 0

 0

 .000120914110677

 0

 0

 0

 4.03047035589E-05

 0

 2.01523517795E-05

 0

 0

 0

 .000151142638346

 0

 0

 0

 0

 0

 0

 0

 0

 0

 0

 0

 0

 0

 0

 0

 0

 0

 0

 0

 0

 0

 0

 0

 0

 0

 0

 0

 0

 0

 2.821329249125E-04

 2.01523517795E-05

 0

 2.01523517795E-05

 0

 0

 0

 0

 .000261980573133

 .000261980573133

 0

 0

 0

 0

 0

 0

 0

 0

 0

 0

 0

 0

 0

 2.01523517795E-05

 2.01523517795E-05

 2.01523517795E-05

 0

 0

 0

 1.7935593083753E-03

 1.5718834388011E-03

 0

 0

 7.05332312281E-05

 0

 .00124944581033

 0

 .000251904397243

 2.216758695742E-04

 0

 0

 5.03808794486E-05

 5.03808794486E-05

 0

 .000120914110677

 0

 0

 0

 0

 0

 0

 0

 0

 0

 0

 0

 0

 0

 0

 0

 0

 0

 1.612188142358E-04

 1.612188142358E-04

 6.04570553384E-05

 6.04570553384E-05

 0

 6.04570553384E-05

 0

 0

 0

 0

 0

 0

 0

 0

 0

 0

 0

 0

 0

 0

 0

 0

 0

 0

 0

 0

 0

 0

 0

 1.007617588974E-04

 2.01523517795E-05

 2.01523517795E-05

 0

 0

 6.04570553384E-05

 6.04570553384E-05

 2.01523517795E-05

 2.01523517795E-05

 0

 0

 0

 0

 0

 0

 0

 0

 0

 0

 0

 0
