# Supplementary material for: First Insight into Microbiome Profiles of Myrmecophilous Beetles and Their Host, Red Wood Ant Formica polyctena (Hymenoptera: Formicidae)—A Case Study
Source: Insects. 2020 Feb 19;11(2):134. doi: 10.3390/insects11020134 (PMC7073670; doi:10.3390/insects11020134)
Supplement: Supplementary file 1 [file insects-11-00134-s001.zip › Supplementary Files/S2_Figure.zip!/Supplemental_Figure_S2/Supplemental_Figure_S2_FPL1.html]

Javascript must be enabled to view this page.

magnitude

 1.00000000000025

 1.00000000000025

 0

 0

 0

 0

 0

 0

 1.3358268768366E-04

 1.3358268768366E-04

 1.3358268768366E-04

 1.3358268768366E-04

 0

 0

 3.81664821953E-05

 0

 9.54162054883E-06

 0

 0

 7.63329643907E-05

 9.54162054883E-06

 0

 0

 0

 0

 0

 0

 0

 0

 0

 0

 0

 0

 0

 0

 0

 0

 0

 0

 0

 0

 0

 0

 0

 0

 0

 0

 0

 0

 0

 0

 0

 0

 0

 0

 0

 0

 0

 0

 0

 0

 0

 0

 0

 0

 0

 0

 0

 0

 0

 0

 0

 0

 0

 0

 0

 0

 0

 0

 0

 0

 0

 0

 0

 0

 0

 0

 0

 0

 0

 0

 0

 0

 1.05053242242675E-02

 0

 0

 0

 0

 7.63329643907E-05

 0

 0

 0

 0

 0

 0

 0

 0

 0

 0

 0

 0

 0

 .000057249723293

 .000057249723293

 .000057249723293

 0

 0

 0

 0

 0

 0

 0

 0

 1.90832410977E-05

 0

 0

 0

 0

 1.90832410977E-05

 1.90832410977E-05

 0

 0

 9.67520323651865E-03

 0

 0

 0

 0

 0

 0

 0

 0

 0

 0

 0

 0

 0

 3.27277584824563E-03

 3.81664821953E-05

 0

 3.81664821953E-05

 0

 0

 0

 0

 0

 0

 0

 .00176519980153

 .00176519980153

 3.5303996030703E-04

 0

 9.54162054883E-06

 .000057249723293

 4.77081027442E-05

 .000104957826037

 0

 .000133582687684

 3.81664821953E-05

 3.81664821953E-05

 .000553413991832

 .000553413991832

 .000524789130186

 .000524789130186

 4.198313041492E-04

 2.86248616465E-05

 2.86248616465E-05

 .000057249723293

 .000057249723293

 0

 0

 0

 .000257623754819

 0

 .000257623754819

 0

 1.90832410977E-05

 0

 1.90832410977E-05

 0

 .000057249723293

 .000057249723293

 0

 0

 0

 0

 0

 0

 0

 0

 0

 0

 0

 0

 0

 0

 1.12591122476212E-03

 0

 0

 0

 0

 0

 0

 0

 0

 .000267165375367

 0

 0

 0

 0

 .000267165375367

 0

 0

 0

 0

 0

 0

 0

 0

 0

 0

 0

 0

 0

 0

 0

 8.5874584939512E-04

 6.67913438418E-05

 0

 3.81664821953E-05

 9.54162054883E-06

 1.90832410977E-05

 2.86248616465E-05

 0

 8.58745849395E-05

 0

 0

 0

 .000124041067135

 .000457997786344

 0

 9.54162054883E-06

 9.54162054883E-06

 0

 0

 0

 0

 9.54162054883E-06

 0

 0

 0

 0

 0

 0

 0

 0

 0

 0

 0

 0

 0

 0

 0

 8.587458493953E-05

 8.587458493953E-05

 9.54162054883E-06

 0

 1.90832410977E-05

 0

 3.81664821953E-05

 1.90832410977E-05

 9.446204343348E-04

 8.492042288465E-04

 2.86248616465E-05

 .00016220754933

 0

 .000133582687684

 .000524789130186

 9.54162054883E-05

 3.81664821953E-05

 .000057249723293

 0

 0

 0

 6.2020533567436E-04

 6.2020533567436E-04

 0

 .000209915652074

 1.90832410977E-05

 9.54162054883E-06

 9.54162054883E-06

 .000372123201405

 0

 .00314873478112

 .00314873478112

 0

 0

 .00314873478112

 .000057249723293

 .000057249723293

 0

 0

 .000057249723293

 0

 0

 0

 0

 0

 0

 0

 0

 0

 0

 7.5378802335816E-04

 0

 0

 0

 0

 0

 0

 0

 0

 7.5378802335816E-04

 0

 0

 9.541620548833E-05

 8.58745849395E-05

 9.54162054883E-06

 6.5837181786983E-04

 1.90832410977E-05

 9.54162054883E-05

 .000534330750735

 0

 9.54162054883E-06

 1.90832410977E-05

 1.90832410977E-05

 1.90832410977E-05

 0

 0

 1.90832410977E-05

 1.90832410977E-05

 0

 0

 0

 0

 0

 0

 0

 0

 0

 0

 6.9653830006543E-04

 6.9653830006543E-04

 0

 0

 0

 0

 0

 0

 0

 0

 0

 0

 0

 2.385405137212E-04

 0

 0

 2.385405137212E-04

 0

 0

 0

 0

 0

 0

 .000057249723293

 0

 .000133582687684

 0

 0

 0

 0

 0

 0

 4.77081027442E-05

 1.335826876837E-04

 0

 0

 6.67913438418E-05

 6.67913438418E-05

 0

 0

 0

 6.67913438419E-05

 4.77081027442E-05

 1.90832410977E-05

 1.3358268768383E-04

 0

 0

 9.54162054883E-06

 0

 9.54162054883E-06

 0

 0

 .000124041067135

 0

 .000124041067135

 0

 0

 1.908324109767E-04

 0

 0

 0

 0

 1.908324109767E-04

 0

 6.67913438418E-05

 7.63329643907E-05

 2.86248616465E-05

 1.90832410977E-05

 0

 0

 0

 0

 0

 0

 2.86248616465E-05

 2.86248616465E-05

 2.86248616465E-05

 2.86248616465E-05

 2.86248616465E-05

 0

 0

 0

 0

 0

 0

 0

 0

 0

 0

 0

 0

 0

 0

 0

 0

 0

 0

 0

 0

 0

 0

 0

 0

 0

 0

 0

 0

 0

 0

 0

 0

 0

 0

 0

 0

 0

 0

 0

 0

 0

 0

 0

 0

 0

 0

 0

 0

 0

 0

 0

 0

 0

 0

 0

 0

 0

 0

 0

 0

 0

 0

 0

 0

 0

 0

 0

 0

 0

 0

 0

 0

 0

 0

 0

 0

 0

 0

 0

 0

 0

 0

 0

 0

 0

 0

 0

 0

 0

 0

 0

 0

 0

 0

 0

 0

 0

 0

 0

 0

 0

 0

 0

 1.90832410977E-05

 1.90832410977E-05

 1.90832410977E-05

 1.90832410977E-05

 1.90832410977E-05

 0

 0

 0

 0

 4.102896836002E-04

 .000362581580856

 .00016220754933

 0

 0

 0

 0

 .000057249723293

 0

 .000057249723293

 0

 0

 0

 0

 0

 0

 0

 0

 0

 0

 0

 0

 0

 0

 0

 0

 0

 0

 0

 0

 0

 0

 0

 0

 0

 .000104957826037

 0

 0

 .000104957826037

 0

 0

 .000200374031526

 0

 0

 0

 0

 0

 3.81664821953E-05

 3.81664821953E-05

 0

 0

 0

 0

 .000143124308233

 .000143124308233

 0

 0

 1.90832410977E-05

 0

 1.90832410977E-05

 2.86248616465E-05

 2.86248616465E-05

 0

 0

 0

 0

 0

 0

 0

 0

 2.86248616465E-05

 0

 0

 0

 0

 2.86248616465E-05

 0

 0

 0

 0

 0

 0

 0

 0

 0

 0

 0

 0

 0

 0

 0

 0

 0

 0

 0

 0

 1.90832410977E-05

 1.90832410977E-05

 1.90832410977E-05

 0

 0

 1.90832410977E-05

 0

 0

 0

 0

 0

 0

 0

 0

 0

 0

 0

 0

 0

 0

 0

 4.77081027442E-05

 4.77081027442E-05

 4.77081027442E-05

 4.77081027442E-05

 4.77081027442E-05

 0

 0

 0

 0

 0

 0

 0

 0

 0

 0

 0

 0

 0

 0

 0

 1.90832410977E-05

 0

 0

 0

 0

 0

 0

 0

 0

 0

 0

 0

 0

 1.90832410977E-05

 1.90832410977E-05

 0

 0

 0

 0

 0

 0

 0

 0

 0

 0

 0

 0

 0

 1.90832410977E-05

 1.90832410977E-05

 0

 0

 0

 0

 9.54162054883E-06

 0

 0

 0

 0

 0

 0

 0

 0

 0

 0

 0

 0

 0

 0

 0

 0

 0

 0

 9.54162054883E-06

 0

 0

 0

 0

 9.54162054883E-06

 9.54162054883E-06

 0

 0

 0

 0

 0

 9.54162054883E-06

 0

 0

 0

 0

 0

 0

 0

 0

 0

 0

 .9881111407964

 .971785428037336

 3.53898706155883E-02

 3.53898706155883E-02

 0

 0

 0

 1.90832410977E-05

 0

 0

 0

 0

 0

 0

 3.81664821953E-05

 0

 0

 0

 3.81664821953E-05

 0

 0

 .0352944544101

 0

 1.90832410977E-05

 1.90832410977E-05

 1.90832410977E-05

 0

 0

 0

 1.240410671349E-04

 1.240410671349E-04

 0

 0

 1.90832410977E-05

 0

 .000057249723293

 4.77081027442E-05

 0

 0

 0

 0

 0

 0

 0

 0

 0

 0

 0

 0

 0

 0

 0

 0

 0

 0

 0

 0

 0

 0

 0

 0

 0

 0

 0

 0

 0

 0

 0

 0

 0

 0

 0

 1.45032632342373E-03

 0

 0

 0

 0

 8.7782909049333E-04

 0

 .000238540513721

 0

 0

 .000543872371284

 9.54162054883E-06

 .000057249723293

 0

 2.86248616465E-05

 0

 0

 0

 0

 8.58745849395E-05

 2.86248616465E-05

 0

 3.81664821953E-05

 1.90832410977E-05

 0

 0

 0

 0

 0

 0

 0

 0

 0

 0

 0

 0

 0

 2.767069959165E-04

 0

 .00024808213427

 0

 2.86248616465E-05

 0

 0

 0

 0

 0

 0

 0

 0

 0

 0

 0

 0

 0

 0

 0

 0

 2.099156520744E-04

 8.58745849395E-05

 7.63329643907E-05

 0

 0

 0

 0

 0

 0

 4.77081027442E-05

 0

 0

 0

 0

 0

 3.81664821954E-05

 3.81664821954E-05

 0

 0

 0

 1.90832410977E-05

 1.90832410977E-05

 0

 0

 0

 0

 0

 0

 0

 0

 0

 0

 0

 0

 0

 .931472081218549

 0

 0

 .931462539598

 .931462539598

 9.54162054883E-06

 0

 9.54162054883E-06

 0

 3.2918590893478E-03

 3.2918590893478E-03

 0

 0

 8.58745849395E-05

 0

 0

 0

 0

 0

 0

 0

 .00311056829892

 9.54162054883E-05

 0

 0

 0

 0

 0

 0

 0

 0

 0

 0

 0

 0

 1.90832410977E-05

 0

 0

 0

 0

 0

 0

 0

 0

 0

 0

 0

 0

 0

 1.90832410977E-05

 0

 0

 0

 0

 0

 0

 0

 0

 1.90832410977E-05

 1.90832410977E-05

 0

 0

 0

 0

 0

 0

 0

 0

 0

 0

 0

 0

 0

 0

 0

 0

 0

 0

 0

 0

 0

 0

 0

 0

 1.63066295179664E-02

 0

 0

 0

 3.81664821953E-05

 3.81664821953E-05

 3.81664821953E-05

 0

 0

 0

 0

 0

 4.35097897027102E-03

 0

 0

 4.33189572917332E-03

 .000181290790428

 0

 0

 0

 0

 0

 .00142170146178

 0

 0

 9.54162054883E-06

 0

 0

 0

 0

 0

 0

 0

 .00269073699477

 0

 0

 0

 0

 0

 9.54162054883E-06

 0

 9.54162054883E-06

 0

 0

 0

 0

 0

 0

 0

 0

 9.54162054883E-06

 0

 1.90832410977E-05

 1.90832410977E-05

 0

 0

 0

 0

 0

 0

 0

 0

 0

 0

 0

 0

 0

 0

 0

 0

 0

 0

 0

 0

 0

 0

 0

 0

 0

 0

 0

 0

 0

 0

 0

 0

 0

 0

 0

 8.77829090493206E-03

 8.77829090493206E-03

 .00854929201176

 9.54162054883E-06

 0

 0

 7.63329643907E-05

 0

 0

 0

 1.90832410977E-05

 0

 .000114499446586

 0

 0

 0

 9.54162054883E-06

 0

 0

 0

 0

 0

 0

 0

 0

 0

 0

 0

 0

 0

 0

 0

 0

 0

 0

 4.77081027442E-05

 4.77081027442E-05

 4.77081027442E-05

 0

 0

 9.54162054883E-06

 9.54162054883E-06

 9.54162054883E-06

 0

 0

 0

 2.53807106599183E-03

 2.39494675775883E-03

 .00238540513721

 9.54162054883E-06

 0

 0

 0

 0

 .000143124308233

 .000143124308233

 0

 0

 0

 0

 0

 0

 0

 0

 0

 0

 0

 0

 0

 9.54162054883E-06

 9.54162054883E-06

 9.54162054883E-06

 0

 0

 0

 5.343307507343E-04

 4.770810274413E-04

 3.81664821953E-05

 0

 0

 0

 .000438914545246

 0

 0

 .000057249723293

 0

 0

 0

 0

 0

 .000057249723293

 0

 0

 0

 0

 0

 0

 0

 0

 0

 0

 0

 0

 0

 0

 0

 0

 0

 0

 0

 0

 0

 0

 0

 0

 0

 0

 0

 0

 0

 0

 0

 0

 0

 0

 0

 0

 0

 0

 0

 0

 0

 0

 0

 0

 0

 0

 0

 0

 0

 0

 0

 0

 0

 0

 0

 0

 0

 0

 0

 0

 0

 0

 0

 0

 0

 0

 0
