# Supplementary material for: First Insight into Microbiome Profiles of Myrmecophilous Beetles and Their Host, Red Wood Ant Formica polyctena (Hymenoptera: Formicidae)—A Case Study
Source: Insects. 2020 Feb 19;11(2):134. doi: 10.3390/insects11020134 (PMC7073670; doi:10.3390/insects11020134)
Supplement: Supplementary file 1 [file insects-11-00134-s001.zip › Supplementary Files/S2_Figure.zip!/Supplemental_Figure_S2/Supplemental_Figure_S2_FPL2.html]

Javascript must be enabled to view this page.

magnitude

 .999999999999931

 .999999999999931

 0

 0

 0

 0

 0

 0

 2.9337960824447E-03

 2.3720053432527E-03

 1.8351830813586E-03

 1.8351830813586E-03

 .000124842386487

 .00038701139811

 3.74527159461E-05

 1.24842386487E-05

 0

 4.99369545948E-05

 0

 .00104867604649

 .000174779341082

 0

 0

 0

 0

 0

 0

 5.368222618941E-04

 5.368222618941E-04

 .000499369545948

 3.74527159461E-05

 0

 0

 0

 0

 0

 0

 0

 0

 0

 0

 0

 .000561790739192

 0

 0

 0

 0

 0

 .000561790739192

 .000561790739192

 .000561790739192

 0

 0

 0

 0

 0

 0

 0

 0

 0

 0

 0

 0

 0

 0

 0

 0

 0

 0

 0

 0

 0

 0

 0

 0

 0

 0

 0

 0

 0

 0

 0

 0

 0

 0

 0

 0

 0

 0

 0

 0

 0

 0

 2.41070648306569E-02

 0

 0

 0

 0

 2.247162956766E-04

 0

 0

 0

 0

 0

 0

 0

 0

 0

 0

 0

 0

 0

 .000124842386487

 0

 0

 .000124842386487

 0

 0

 .000124842386487

 0

 0

 0

 0

 9.98739091896E-05

 1.24842386487E-05

 1.24842386487E-05

 0

 0

 8.73896705409E-05

 8.73896705409E-05

 0

 0

 2.20346812149672E-02

 0

 0

 0

 0

 0

 0

 0

 1.24842386487E-05

 1.24842386487E-05

 1.24842386487E-05

 0

 0

 0

 2.9837330370424E-03

 0

 0

 0

 0

 0

 0

 0

 0

 0

 0

 .00137326625136

 .00137326625136

 3.870113981096E-04

 0

 0

 0

 9.98739091896E-05

 .000174779341082

 0

 .000112358147838

 .00057427497784

 .00057427497784

 4.99369545948E-05

 4.99369545948E-05

 .000599243455138

 .000599243455138

 1.5730140697315E-03

 1.24842386487E-05

 1.24842386487E-05

 6.24211932435E-05

 6.24211932435E-05

 2.49684772974E-05

 2.49684772974E-05

 0

 .001260908103514

 0

 .00111109723973

 .000149810863784

 8.73896705409E-05

 0

 6.24211932435E-05

 2.49684772974E-05

 0

 0

 9.98739091896E-05

 9.98739091896E-05

 0

 2.49684772974E-05

 2.49684772974E-05

 0

 0

 7.49054318922E-05

 7.49054318922E-05

 0

 4.99369545948E-05

 1.24842386487E-05

 1.24842386487E-05

 0

 5.2683487097575E-03

 0

 0

 0

 0

 0

 0

 0

 0

 1.2983608194674E-03

 2.49684772974E-05

 0

 0

 0

 .00127339234217

 0

 0

 0

 0

 0

 0

 0

 7.49054318922E-05

 2.49684772974E-05

 0

 0

 0

 2.49684772974E-05

 2.49684772974E-05

 0

 3.8950824583979E-03

 .000499369545948

 0

 0

 7.49054318922E-05

 1.24842386487E-05

 4.99369545948E-05

 0

 .00124842386487

 1.24842386487E-05

 0

 0

 .000174779341082

 .00176027764947

 0

 0

 2.49684772974E-05

 1.24842386487E-05

 0

 0

 0

 2.49684772974E-05

 0

 0

 0

 0

 0

 0

 0

 0

 0

 0

 0

 0

 0

 0

 0

 4.744010686504E-04

 4.744010686504E-04

 2.49684772974E-05

 0

 .000299621727569

 0

 0

 .000149810863784

 1.02121072146438E-02

 .010162170260049

 .000112358147838

 .000324590204866

 0

 .000624211932435

 .00910100997491

 4.99369545948E-05

 1.24842386487E-05

 3.74527159461E-05

 0

 0

 0

 5.118537845966E-04

 5.118537845966E-04

 0

 6.24211932435E-05

 0

 3.74527159461E-05

 0

 .000411979875407

 0

 .000886380944058

 .000886380944058

 0

 0

 .000886380944058

 3.74527159461E-05

 3.74527159461E-05

 0

 0

 3.74527159461E-05

 0

 0

 0

 0

 0

 0

 0

 0

 0

 0

 1.8476673200131E-03

 8.73896705409E-05

 0

 0

 8.73896705409E-05

 4.99369545948E-05

 0

 0

 3.74527159461E-05

 1.7602776494722E-03

 0

 0

 2.372005343255E-04

 .000174779341082

 6.24211932435E-05

 1.5230771151467E-03

 0

 .000374527159461

 .00101122333055

 1.24842386487E-05

 .000124842386487

 2.746532502714E-04

 .000124842386487

 .000124842386487

 4.99369545948E-05

 4.99369545948E-05

 7.49054318922E-05

 7.49054318922E-05

 0

 0

 0

 0

 1.498108637844E-04

 1.498108637844E-04

 1.498108637844E-04

 2.49684772974E-05

 0

 .000124842386487

 5.0186639367731E-03

 5.0186639367731E-03

 0

 0

 0

 0

 0

 0

 0

 0

 0

 0

 0

 1.9974781837908E-03

 0

 0

 1.9974781837908E-03

 .000124842386487

 0

 .000162295102433

 2.49684772974E-05

 2.49684772974E-05

 0

 .000174779341082

 0

 .00113606571703

 8.73896705409E-05

 0

 0

 3.74527159461E-05

 0

 0

 .000224716295677

 3.495586821628E-04

 0

 0

 .000149810863784

 .000149810863784

 0

 0

 0

 1.997478183788E-04

 .000149810863784

 4.99369545948E-05

 .000811475512166

 0

 0

 .000274653250272

 0

 .000274653250272

 0

 0

 .000536822261894

 0

 .000536822261894

 0

 0

 1.8601515586535E-03

 0

 0

 0

 0

 1.8601515586535E-03

 0

 .00179773036541

 2.49684772974E-05

 3.74527159461E-05

 0

 0

 0

 0

 0

 0

 0

 1.24842386487E-05

 1.24842386487E-05

 1.24842386487E-05

 1.24842386487E-05

 1.24842386487E-05

 0

 0

 0

 4.99369545948E-05

 3.74527159461E-05

 3.74527159461E-05

 3.74527159461E-05

 3.74527159461E-05

 0

 0

 0

 0

 0

 0

 0

 0

 0

 0

 0

 0

 0

 0

 0

 0

 0

 0

 0

 0

 0

 0

 0

 0

 0

 0

 0

 0

 0

 0

 1.24842386487E-05

 1.24842386487E-05

 1.24842386487E-05

 1.24842386487E-05

 0

 0

 0

 0

 0

 0

 0

 0

 0

 0

 0

 0

 0

 0

 0

 0

 0

 0

 1.24842386487E-05

 1.24842386487E-05

 1.24842386487E-05

 1.24842386487E-05

 1.24842386487E-05

 0

 0

 0

 0

 0

 0

 0

 0

 0

 0

 0

 0

 0

 0

 0

 0

 0

 0

 0

 0

 0

 0

 0

 0

 0

 0

 0

 0

 0

 0

 0

 0

 0

 0

 0

 0

 0

 0

 4.99369545948E-05

 1.24842386487E-05

 1.24842386487E-05

 1.24842386487E-05

 1.24842386487E-05

 3.74527159461E-05

 3.74527159461E-05

 3.74527159461E-05

 3.74527159461E-05

 1.75902922560151E-02

 .017552839540069

 2.496847729742E-04

 0

 0

 0

 0

 2.49684772974E-05

 0

 2.49684772974E-05

 0

 0

 0

 0

 0

 0

 0

 0

 0

 0

 4.99369545948E-05

 0

 0

 0

 4.99369545948E-05

 0

 0

 0

 0

 0

 0

 0

 0

 0

 0

 .000174779341082

 0

 0

 .000174779341082

 0

 0

 1.73031547670948E-02

 0

 0

 0

 0

 0

 0

 0

 0

 0

 0

 0

 .0172532178125

 .0172532178125

 0

 0

 4.99369545948E-05

 1.24842386487E-05

 3.74527159461E-05

 3.74527159461E-05

 3.74527159461E-05

 0

 0

 0

 0

 0

 0

 0

 0

 3.74527159461E-05

 3.74527159461E-05

 0

 0

 0

 0

 0

 0

 0

 0

 0

 0

 0

 0

 0

 0

 0

 0

 0

 0

 0

 0

 0

 0

 0

 0

 0

 0

 0

 0

 0

 0

 0

 0

 0

 0

 0

 0

 0

 0

 0

 0

 0

 0

 0

 0

 0

 .000237200534325

 .000237200534325

 .000237200534325

 .000237200534325

 .000124842386487

 .000112358147838

 0

 0

 0

 0

 0

 0

 0

 0

 0

 0

 0

 0

 0

 0

 5.617907391915E-04

 0

 0

 0

 0

 0

 0

 0

 0

 0

 0

 0

 0

 5.617907391915E-04

 5.617907391915E-04

 9.98739091896E-05

 9.98739091896E-05

 0

 0

 0

 0

 0

 .000249684772974

 .000249684772974

 3.74527159461E-05

 3.74527159461E-05

 .000124842386487

 .000124842386487

 4.99369545948E-05

 4.99369545948E-05

 0

 0

 0

 0

 5.992434551375E-04

 7.49054318922E-05

 0

 0

 0

 0

 7.49054318922E-05

 0

 0

 0

 0

 7.49054318922E-05

 4.99369545948E-05

 2.49684772974E-05

 0

 0

 0

 0

 0

 5.243380232453E-04

 4.99369545948E-05

 4.99369545948E-05

 4.99369545948E-05

 0

 4.744010686505E-04

 4.744010686505E-04

 3.74527159461E-05

 0

 0

 0

 .000411979875407

 2.49684772974E-05

 0

 0

 0

 0

 0

 0

 0

 0

 0

 0

 .948315251994303

 .896081197488181

 8.4393453265253E-03

 8.4393453265253E-03

 0

 0

 0

 3.74527159461E-05

 4.99369545948E-05

 0

 2.49684772974E-05

 0

 6.24211932435E-05

 0

 2.49684772974E-05

 3.74527159461E-05

 0

 0

 0

 0

 0

 .0082021447922

 0

 0

 0

 0

 0

 0

 0

 .001672887978926

 .001672887978926

 .000237200534325

 .000124842386487

 0

 .000137326625136

 .000749054318922

 .000424464114056

 0

 0

 0

 0

 0

 0

 0

 2.49684772974E-05

 2.49684772974E-05

 0

 0

 0

 0

 2.49684772974E-05

 0

 4.99369545948E-05

 4.99369545948E-05

 0

 4.99369545948E-05

 .000461916830002

 .000461916830002

 0

 .000461916830002

 0

 0

 0

 0

 0

 0

 0

 0

 6.24211932435E-05

 6.24211932435E-05

 6.24211932435E-05

 0

 5.5804546759711E-03

 7.49054318922E-05

 7.49054318922E-05

 4.99369545948E-05

 4.99369545948E-05

 2.1597732862229E-03

 .000112358147838

 .000461916830002

 0

 .000149810863784

 .00123593962622

 1.24842386487E-05

 0

 0

 6.24211932435E-05

 1.24842386487E-05

 .000112358147838

 0

 0

 .000112358147838

 .000112358147838

 0

 0

 0

 0

 0

 0

 0

 0

 0

 0

 .000162295102433

 .000162295102433

 0

 0

 0

 0

 1.6604037402818E-03

 2.49684772974E-05

 .0014856243992

 0

 6.24211932435E-05

 0

 0

 6.24211932435E-05

 0

 0

 0

 0

 2.49684772974E-05

 0

 0

 3.74527159461E-05

 0

 3.74527159461E-05

 0

 2.49684772974E-05

 2.49684772974E-05

 1.2983608194649E-03

 .000786507034868

 .000149810863784

 2.49684772974E-05

 3.74527159461E-05

 0

 0

 0

 2.49684772974E-05

 .000274653250272

 0

 0

 0

 0

 0

 0

 0

 0

 0

 0

 0

 0

 0

 0

 0

 0

 0

 0

 0

 0

 0

 0

 0

 0

 0

 .867816881187541

 2.49684772974E-05

 2.49684772974E-05

 .867729491517

 .867729491517

 6.24211932435E-05

 0

 3.74527159461E-05

 2.49684772974E-05

 1.19723848640802E-02

 1.19723848640802E-02

 .000174779341082

 .000237200534325

 9.98739091896E-05

 0

 0

 0

 6.24211932435E-05

 2.49684772974E-05

 1.24842386487E-05

 .000174779341082

 .0108238349084

 .000362042920812

 0

 0

 0

 0

 0

 0

 0

 0

 0

 0

 0

 0

 9.238336600038E-04

 0

 0

 0

 0

 0

 0

 0

 0

 0

 0

 0

 0

 0

 8.863809440577E-04

 5.742749778403E-04

 .000461916830002

 8.73896705409E-05

 2.49684772974E-05

 0

 0

 0

 0

 .000162295102433

 .000162295102433

 2.49684772974E-05

 2.49684772974E-05

 0

 0

 0

 0

 0

 0

 0

 0

 0

 0

 0

 0

 0

 .000124842386487

 .000124842386487

 3.74527159461E-05

 3.74527159461E-05

 0

 3.74527159461E-05

 0

 0

 0

 5.13102208461179E-02

 0

 0

 0

 1.24842386487E-05

 1.24842386487E-05

 1.24842386487E-05

 0

 0

 0

 0

 0

 2.46189186152119E-02

 0

 0

 2.42194229784538E-02

 .00225964719542

 0

 0

 1.24842386487E-05

 7.49054318922E-05

 0

 .00756544862112

 0

 0

 2.49684772974E-05

 0

 0

 0

 0

 1.24842386487E-05

 0

 0

 .0135703674111

 .000124842386487

 .000149810863784

 0

 0

 0

 7.49054318922E-05

 2.49684772974E-05

 0

 8.73896705409E-05

 8.73896705409E-05

 0

 0

 2.49684772974E-05

 0

 0

 0

 0

 .000124842386487

 .000362042920812

 .000362042920812

 0

 0

 0

 0

 0

 0

 0

 0

 0

 2.49684772974E-05

 2.49684772974E-05

 0

 0

 0

 0

 1.24842386487E-05

 0

 1.24842386487E-05

 0

 0

 0

 0

 0

 0

 0

 0

 0

 0

 0

 0

 0

 0

 0

 0

 0

 1.84267362454588E-02

 1.84267362454588E-02

 .0180272406087

 0

 0

 0

 .000224716295677

 1.24842386487E-05

 0

 0

 2.49684772974E-05

 0

 9.98739091896E-05

 0

 0

 0

 1.24842386487E-05

 2.49684772974E-05

 0

 0

 0

 0

 0

 0

 0

 0

 0

 0

 0

 0

 0

 0

 0

 0

 0

 3.74527159461E-05

 3.74527159461E-05

 3.74527159461E-05

 0

 0

 0

 0

 0

 0

 0

 0

 .003395712912449

 .000973770614599

 .000973770614599

 0

 0

 0

 0

 0

 .00242194229785

 .00242194229785

 0

 0

 0

 .000149810863784

 .000149810863784

 .000149810863784

 0

 0

 0

 0

 0

 0

 0

 0

 0

 0

 0

 0

 0

 4.6691052546194E-03

 4.4194204816449E-03

 .000124842386487

 0

 9.98739091896E-05

 1.24842386487E-05

 .00408234603813

 0

 9.98739091896E-05

 2.496847729745E-04

 0

 0

 0

 .000187263579731

 0

 2.49684772974E-05

 0

 3.74527159461E-05

 0

 0

 0

 0

 0

 0

 0

 0

 0

 0

 0

 0

 0

 0

 0

 2.372005343252E-04

 2.372005343252E-04

 2.247162956765E-04

 4.99369545948E-05

 2.49684772974E-05

 2.49684772974E-05

 .000162295102433

 .000162295102433

 1.24842386487E-05

 1.24842386487E-05

 0

 0

 0

 0

 0

 0

 0

 0

 0

 0

 0

 0

 0

 0

 0

 0

 0

 0

 0

 1.24842386487E-05

 0

 0

 0

 0

 1.24842386487E-05

 1.24842386487E-05

 0

 0

 0

 0

 0

 0

 0

 0

 0

 0

 0

 0

 0

 0
