# Supplementary material for: First Insight into Microbiome Profiles of Myrmecophilous Beetles and Their Host, Red Wood Ant Formica polyctena (Hymenoptera: Formicidae)—A Case Study
Source: Insects. 2020 Feb 19;11(2):134. doi: 10.3390/insects11020134 (PMC7073670; doi:10.3390/insects11020134)
Supplement: Supplementary file 1 [file insects-11-00134-s001.zip › Supplementary Files/S2_Figure.zip!/Supplemental_Figure_S2/Supplemental_Figure_S2_FPL3.html]

Javascript must be enabled to view this page.

magnitude

 1.00000000000032

 1.00000000000032

 0

 0

 0

 0

 0

 0

 7.335300919932E-04

 7.335300919932E-04

 .000612713370959

 .000612713370959

 .000189854847339

 0

 .000310671568374

 0

 0

 7.76678920934E-05

 0

 3.45190631526E-05

 0

 0

 0

 0

 0

 0

 0

 1.208167210342E-04

 1.208167210342E-04

 7.76678920934E-05

 4.31488289408E-05

 0

 0

 0

 0

 0

 0

 0

 0

 0

 0

 0

 0

 0

 0

 0

 0

 0

 0

 0

 0

 0

 0

 0

 0

 0

 0

 0

 0

 0

 0

 0

 0

 0

 0

 0

 0

 0

 0

 0

 0

 0

 0

 0

 0

 0

 0

 0

 0

 0

 0

 0

 0

 0

 0

 0

 0

 0

 0

 0

 0

 0

 0

 6.29972902535732E-03

 0

 0

 0

 0

 1.3807625261052E-04

 6.04083605171E-05

 6.04083605171E-05

 1.72595315763E-05

 4.31488289408E-05

 0

 0

 0

 0

 0

 0

 0

 0

 0

 0

 0

 0

 0

 0

 0

 0

 0

 0

 0

 0

 7.766789209342E-05

 8.62976578816E-06

 8.62976578816E-06

 0

 0

 6.04083605171E-05

 6.04083605171E-05

 8.62976578816E-06

 8.62976578816E-06

 6.02357652013634E-03

 5.17785947289E-05

 5.17785947289E-05

 5.17785947289E-05

 0

 0

 0

 0

 6.04083605171E-05

 6.04083605171E-05

 6.04083605171E-05

 0

 0

 0

 3.05493708901016E-03

 .000345190631527

 0

 0

 .000138076252611

 .000207114378916

 0

 0

 0

 0

 0

 .000621343136747

 .000621343136747

 8.62976578816E-06

 0

 8.62976578816E-06

 0

 0

 0

 0

 0

 .000897495641968

 .000897495641968

 0

 0

 .00118227791298

 .00118227791298

 .000362450163103

 0

 0

 0

 0

 0

 0

 0

 .000362450163103

 0

 .000250263207857

 .000112186955246

 0

 0

 0

 0

 0

 0

 0

 0

 0

 0

 0

 0

 0

 4.31488289408E-05

 4.31488289408E-05

 0

 0

 4.31488289408E-05

 0

 0

 9.4064447090942E-04

 0

 0

 0

 0

 0

 0

 0

 0

 .000172595315763

 0

 0

 0

 0

 .000172595315763

 0

 0

 0

 0

 0

 0

 0

 0

 0

 0

 0

 0

 0

 0

 0

 3.8833946046732E-04

 0

 0

 8.62976578816E-06

 0

 0

 0

 0

 0

 0

 0

 0

 .000138076252611

 .00023300367628

 0

 0

 0

 0

 0

 0

 0

 8.62976578816E-06

 0

 0

 3.106715683738E-04

 0

 4.31488289408E-05

 0

 0

 0

 0

 .000267522739433

 0

 0

 0

 6.90381263053E-05

 6.90381263053E-05

 0

 0

 0

 0

 0

 0

 0

 0

 8.6297657881596E-04

 5.6093477623016E-04

 8.62976578816E-06

 0

 0

 .000155335784187

 .000396969226255

 3.020418025858E-04

 .000258892973645

 4.31488289408E-05

 0

 0

 0

 .000189854847339

 .000189854847339

 0

 .000189854847339

 0

 0

 0

 0

 0

 .000457377586772

 .000457377586772

 0

 0

 .000457377586772

 0

 0

 0

 0

 0

 0

 0

 0

 0

 0

 0

 0

 0

 0

 0

 1.3807625261046E-04

 9.49274236697E-05

 0

 0

 9.49274236697E-05

 7.76678920934E-05

 0

 0

 1.72595315763E-05

 4.314882894076E-05

 0

 0

 8.62976578816E-06

 8.62976578816E-06

 0

 3.45190631526E-05

 0

 0

 3.45190631526E-05

 0

 0

 2.847822710096E-04

 0

 0

 0

 0

 0

 0

 0

 0

 0

 0

 2.847822710096E-04

 2.847822710096E-04

 2.847822710096E-04

 0

 .000250263207857

 3.45190631526E-05

 7.939384525096E-04

 7.939384525096E-04

 2.330036762799E-04

 0

 0

 0

 0

 2.330036762799E-04

 0

 5.17785947289E-05

 .000181225081551

 0

 0

 1.467060183986E-04

 0

 0

 1.467060183986E-04

 0

 7.76678920934E-05

 3.45190631526E-05

 0

 0

 0

 0

 0

 3.45190631526E-05

 0

 0

 0

 0

 0

 0

 0

 0

 0

 0

 0

 0

 0

 0

 0

 0

 0

 0

 3.797096946785E-04

 0

 0

 0

 0

 0

 0

 0

 3.797096946785E-04

 0

 .000353820397314

 2.58892973645E-05

 0

 3.45190631526E-05

 0

 0

 0

 0

 3.45190631526E-05

 0

 3.45190631526E-05

 0

 0

 0

 0

 0

 0

 0

 0

 0

 0

 0

 0

 0

 0

 0

 0

 0

 0

 0

 0

 0

 0

 0

 0

 0

 0

 0

 0

 0

 0

 0

 0

 0

 0

 0

 0

 0

 0

 0

 0

 0

 0

 0

 0

 0

 0

 0

 0

 0

 0

 0

 0

 0

 0

 0

 0

 0

 0

 0

 0

 0

 0

 0

 0

 0

 0

 0

 0

 0

 0

 0

 0

 0

 0

 0

 0

 0

 0

 0

 0

 0

 0

 0

 0

 0

 0

 0

 0

 0

 0

 0

 0

 0

 0

 0

 0

 0

 0

 0

 0

 0

 0

 0

 0

 0

 0

 0

 0

 0

 0

 0

 0

 0

 0

 0

 0

 0

 0

 0

 0

 0

 0

 0

 0

 0

 0

 .029263535787619

 2.88665665613645E-02

 1.0873504893083E-03

 0

 0

 0

 0

 .000535045478866

 0

 .000535045478866

 0

 0

 0

 0

 0

 0

 0

 9.49274236697E-05

 9.49274236697E-05

 0

 8.62976578816E-05

 0

 0

 0

 0

 8.62976578816E-05

 0

 0

 0

 0

 0

 0

 0

 0

 0

 .000371079928891

 0

 0

 .000371079928891

 0

 0

 2.77792160720562E-02

 0

 0

 0

 0

 0

 0

 0

 0

 8.62976578816E-05

 0

 8.62976578816E-05

 .0275289528642

 .0275289528642

 0

 0

 1.639655499746E-04

 3.45190631526E-05

 .000129446486822

 .00037107992889

 .00037107992889

 .000120816721034

 .000120816721034

 0

 0

 0

 0

 0

 0

 0

 0

 0

 0

 0

 0

 0

 0

 0

 .000120816721034

 0

 0

 0

 0

 .000120816721034

 0

 0

 0

 0

 .000129446486822

 0

 0

 0

 .000129446486822

 0

 0

 2.58892973645E-05

 2.58892973645E-05

 2.58892973645E-05

 2.58892973645E-05

 0

 0

 0

 0

 0

 0

 0

 0

 0

 0

 0

 0

 0

 0

 0

 0

 0

 7.76678920934E-05

 0

 0

 0

 0

 0

 7.76678920934E-05

 7.76678920934E-05

 7.76678920934E-05

 7.76678920934E-05

 9.49274236697E-05

 9.49274236697E-05

 9.49274236697E-05

 9.49274236697E-05

 9.49274236697E-05

 0

 0

 0

 0

 0

 1.035571894579E-04

 0

 0

 0

 0

 0

 0

 0

 0

 0

 0

 0

 0

 1.035571894579E-04

 1.035571894579E-04

 6.04083605171E-05

 6.04083605171E-05

 0

 0

 0

 0

 0

 4.31488289408E-05

 4.31488289408E-05

 0

 0

 0

 0

 0

 0

 0

 0

 0

 0

 1.5533578418666E-04

 0

 0

 0

 0

 0

 0

 0

 0

 0

 0

 0

 0

 0

 0

 0

 0

 0

 0

 1.5533578418666E-04

 .000120816721034

 .000120816721034

 .000120816721034

 0

 3.451906315266E-05

 3.451906315266E-05

 8.62976578816E-06

 0

 0

 0

 2.58892973645E-05

 0

 0

 0

 0

 0

 0

 0

 0

 0

 0

 0

 .962192996082421

 .952018502218183

 6.62679714872763E-02

 6.62679714872763E-02

 0

 0

 0

 0

 0

 0

 0

 0

 0

 0

 0

 1.72595315763E-05

 0

 0

 0

 0

 0

 .0662507119557

 0

 0

 0

 0

 0

 0

 0

 .000267522739433

 .000267522739433

 0

 0

 0

 0

 .000155335784187

 .000112186955246

 0

 0

 0

 0

 0

 0

 0

 0

 0

 0

 0

 0

 0

 0

 0

 0

 0

 0

 0

 0

 0

 0

 0

 0

 0

 0

 0

 0

 0

 0

 0

 .000172595315763

 .000172595315763

 .000172595315763

 0

 1.20816721034232E-03

 0

 0

 0

 0

 .000362450163103

 0

 .000138076252611

 0

 0

 .000224373910492

 0

 0

 0

 0

 0

 0

 0

 0

 5.17785947289E-05

 3.45190631526E-05

 0

 1.72595315763E-05

 0

 0

 0

 0

 0

 0

 0

 0

 3.45190631526E-05

 3.45190631526E-05

 .000120816721034

 .000120816721034

 0

 0

 .000431488289408

 .000172595315763

 .000258892973645

 0

 0

 0

 0

 0

 0

 0

 0

 0

 0

 0

 0

 0

 0

 0

 0

 0

 0

 2.0711437891582E-04

 8.62976578816E-06

 2.58892973645E-05

 0

 8.62976578816E-06

 0

 0

 6.04083605171E-05

 0

 8.62976578816E-05

 1.72595315763E-05

 0

 0

 0

 0

 .000224373910492

 .000224373910492

 0

 0

 0

 .000172595315763

 2.58892973645E-05

 0

 0

 2.58892973645E-05

 0

 0

 0

 0

 0

 0

 0

 0

 0

 0

 .881021419079

 0

 0

 .881021419079

 .881021419079

 0

 0

 0

 0

 2.8564524758768E-03

 2.8564524758768E-03

 7.76678920934E-05

 0

 5.17785947289E-05

 0

 0

 0

 0

 2.58892973645E-05

 0

 0

 .00270111669169

 0

 0

 0

 0

 0

 0

 0

 0

 0

 0

 0

 0

 0

 0

 0

 0

 0

 0

 0

 0

 0

 0

 0

 0

 0

 0

 0

 0

 0

 0

 0

 0

 0

 0

 0

 0

 0

 0

 0

 0

 0

 0

 0

 0

 0

 0

 0

 0

 0

 0

 0

 0

 0

 0

 0

 0

 0

 0

 0

 0

 0

 0

 1.01744938642378E-02

 0

 0

 0

 2.58892973645E-05

 2.58892973645E-05

 2.58892973645E-05

 .000103557189458

 0

 0

 .000103557189458

 .000103557189458

 3.6590206941764E-03

 0

 0

 3.6590206941764E-03

 .000241633442068

 0

 0

 0

 0

 0

 .0023300367628

 0

 2.58892973645E-05

 0

 0

 0

 0

 0

 0

 0

 0

 .000871606344604

 0

 0

 0

 0

 0

 0

 0

 0

 0

 .000138076252611

 0

 0

 0

 0

 0

 0

 5.17785947289E-05

 0

 0

 0

 0

 0

 0

 0

 0

 0

 0

 0

 0

 0

 0

 0

 0

 0

 0

 0

 0

 0

 0

 0

 0

 0

 0

 0

 0

 0

 0

 0

 0

 0

 0

 0

 0

 0

 0

 4.0991387493774E-03

 4.0991387493774E-03

 .00380572671258

 0

 0

 0

 5.17785947289E-05

 0

 .000215744144704

 0

 0

 0

 0

 0

 0

 0

 2.58892973645E-05

 0

 0

 0

 0

 0

 0

 0

 0

 0

 0

 0

 0

 0

 0

 0

 0

 0

 0

 .000362450163103

 .000362450163103

 .000362450163103

 0

 0

 0

 0

 0

 0

 0

 0

 .000578194307806

 .000457377586772

 .000457377586772

 0

 0

 0

 0

 0

 .000120816721034

 .000120816721034

 0

 0

 0

 0

 0

 0

 0

 0

 0

 0

 0

 0

 0

 .000526415713078

 .000526415713078

 .000526415713078

 0

 0

 0

 8.1982774987446E-04

 8.111979840863E-04

 0

 0

 0

 0

 .000742159857781

 0

 6.90381263053E-05

 8.62976578816E-06

 0

 0

 0

 0

 0

 8.62976578816E-06

 0

 0

 0

 0

 0

 0

 0

 0

 0

 0

 0

 0

 0

 0

 0

 0

 0

 0

 0

 0

 0

 0

 0

 0

 0

 0

 0

 0

 0

 0

 0

 0

 0

 0

 0

 0

 0

 0

 0

 0

 0

 0

 0

 0

 0

 0

 0

 0

 0

 0

 0

 0

 0

 0

 0

 0

 0

 0

 0

 0

 0

 0

 0

 0

 0

 0

 0
