# Supplementary material for: First Insight into Microbiome Profiles of Myrmecophilous Beetles and Their Host, Red Wood Ant Formica polyctena (Hymenoptera: Formicidae)—A Case Study
Source: Insects. 2020 Feb 19;11(2):134. doi: 10.3390/insects11020134 (PMC7073670; doi:10.3390/insects11020134)
Supplement: Supplementary file 1 [file insects-11-00134-s001.zip › Supplementary Files/S2_Figure.zip!/Supplemental_Figure_S2/Supplemental_Figure_S2_LF1.html]

Javascript must be enabled to view this page.

magnitude

 .999999999999929

 .999999999999929

 0

 0

 0

 0

 0

 0

 2.0148278738834E-03

 2.0148278738834E-03

 1.5583434337064E-03

 1.5583434337064E-03

 .000330557698059

 0

 .000550929496765

 0

 .000220371798706

 3.14816855294E-05

 0

 0

 .000425002754647

 0

 0

 0

 0

 0

 0

 .000456484440177

 .000456484440177

 .000456484440177

 0

 0

 0

 0

 0

 0

 0

 0

 0

 0

 0

 0

 0

 0

 0

 0

 0

 0

 0

 0

 0

 0

 0

 0

 0

 0

 0

 0

 0

 0

 0

 0

 0

 0

 0

 0

 0

 0

 0

 0

 0

 0

 0

 0

 0

 0

 0

 0

 0

 0

 0

 0

 0

 0

 0

 0

 0

 0

 0

 0

 0

 0

 0

 2.08723575060241E-02

 0

 0

 0

 0

 3.148168552944E-04

 0

 0

 0

 0

 4.72225282942E-05

 0

 0

 0

 0

 4.72225282942E-05

 4.72225282942E-05

 0

 0

 0

 0

 0

 0

 0

 0

 0

 0

 0

 0

 0

 2.675943270002E-04

 0

 0

 4.72225282942E-05

 4.72225282942E-05

 .000220371798706

 .000220371798706

 0

 0

 1.99751294684346E-02

 0

 0

 0

 0

 0

 0

 0

 1.57408427647E-05

 1.57408427647E-05

 1.57408427647E-05

 .000141667584882

 .000141667584882

 .000141667584882

 5.5250358104151E-03

 .000346298540824

 0

 0

 .000346298540824

 0

 0

 0

 0

 0

 0

 .00237686725747

 .00237686725747

 2.5342756851211E-03

 0

 .000393521069118

 6.29633710589E-05

 .00203056871665

 0

 0

 4.72225282942E-05

 0

 0

 .000267594327

 .000267594327

 0

 0

 5.194478112359E-04

 0

 0

 6.29633710589E-05

 6.29633710589E-05

 0

 0

 0

 .000220371798706

 0

 .000220371798706

 0

 .000110185899353

 0

 .000110185899353

 0

 0

 0

 0

 0

 0

 .000125926742118

 .000125926742118

 0

 0

 0

 0

 0

 0

 0

 0

 0

 1.1333406790598E-03

 0

 0

 0

 0

 0

 0

 0

 0

 1.101858993531E-04

 0

 0

 0

 0

 6.29633710589E-05

 0

 4.72225282942E-05

 0

 0

 0

 0

 0

 0

 0

 0

 0

 0

 0

 0

 0

 .000362039383588

 0

 0

 0

 0

 0

 0

 0

 0

 0

 .000204630955941

 0

 0

 .000157408427647

 0

 0

 0

 0

 0

 0

 0

 0

 0

 0

 6.611153961187E-04

 0

 0

 1.57408427647E-05

 .000645374553354

 0

 0

 0

 0

 0

 0

 0

 0

 2.990760125287E-04

 2.990760125287E-04

 0

 1.57408427647E-05

 .000141667584882

 0

 0

 .000141667584882

 4.9583654708894E-03

 0

 0

 0

 0

 0

 0

 4.9583654708894E-03

 .00492688378536

 3.14816855294E-05

 0

 0

 0

 .000330557698059

 .000330557698059

 0

 .000141667584882

 0

 0

 0

 .000188890113177

 0

 .00648522721907

 .00648522721907

 0

 0

 .00648522721907

 .00056667033953

 .00056667033953

 0

 0

 .00056667033953

 0

 0

 0

 0

 0

 0

 0

 0

 0

 0

 5.824111822951E-04

 3.14816855294E-05

 0

 0

 3.14816855294E-05

 0

 0

 0

 3.14816855294E-05

 5.509294967657E-04

 0

 0

 4.250027546477E-04

 .000409261911883

 1.57408427647E-05

 .000125926742118

 0

 0

 0

 .000125926742118

 0

 3.14816855294E-05

 3.14816855294E-05

 3.14816855294E-05

 3.14816855294E-05

 3.14816855294E-05

 0

 0

 0

 0

 0

 0

 0

 0

 0

 0

 0

 0

 3.462985408239E-04

 3.462985408239E-04

 .000110185899353

 .000110185899353

 .000110185899353

 0

 0

 0

 0

 0

 0

 0

 0

 7.87042138236E-05

 0

 0

 7.87042138236E-05

 0

 0

 0

 0

 0

 0

 0

 0

 0

 0

 0

 0

 0

 0

 0

 7.87042138236E-05

 9.44450565884E-05

 0

 0

 0

 0

 9.44450565884E-05

 4.72225282942E-05

 4.72225282942E-05

 0

 0

 0

 0

 0

 0

 0

 0

 0

 0

 0

 0

 0

 0

 0

 0

 6.29633710589E-05

 0

 0

 0

 0

 6.29633710589E-05

 0

 6.29633710589E-05

 0

 0

 0

 0

 0

 0

 0

 0

 0

 0

 0

 0

 0

 0

 0

 0

 0

 1.57408427647E-05

 0

 0

 0

 0

 0

 0

 0

 0

 0

 0

 0

 0

 0

 0

 0

 0

 0

 0

 0

 0

 0

 0

 0

 0

 0

 0

 0

 0

 0

 0

 0

 0

 0

 0

 0

 0

 0

 0

 0

 0

 0

 0

 0

 0

 0

 1.57408427647E-05

 1.57408427647E-05

 1.57408427647E-05

 1.57408427647E-05

 0

 0

 0

 0

 0

 0

 0

 0

 0

 0

 0

 0

 0

 0

 0

 0

 0

 0

 0

 0

 0

 0

 0

 0

 0

 0

 0

 0

 0

 0

 0

 0

 0

 0

 0

 0

 0

 0

 0

 0

 0

 0

 0

 0

 0

 0

 0

 0

 0

 0

 0

 0

 0

 0

 0

 0

 0

 0

 0

 2.5185348423551E-03

 2.3453855719432E-03

 9.759322514125E-04

 0

 0

 0

 0

 0

 0

 0

 0

 0

 0

 .000125926742118

 .000125926742118

 0

 0

 0

 0

 0

 9.44450565883E-05

 0

 0

 0

 9.44450565883E-05

 0

 0

 0

 0

 0

 0

 0

 0

 0

 0

 7.555604527062E-04

 4.72225282942E-05

 0

 .000708337924412

 0

 0

 1.3694533205307E-03

 0

 0

 0

 0

 0

 0

 0

 0

 8.500055092952E-04

 4.72225282942E-05

 .000802782981001

 7.87042138236E-05

 7.87042138236E-05

 6.29633710589E-05

 6.29633710589E-05

 .000377780226353

 0

 .000377780226353

 1.574084276472E-04

 1.574084276472E-04

 0

 0

 0

 0

 0

 0

 0

 0

 1.259267421178E-04

 6.29633710589E-05

 0

 0

 0

 6.29633710589E-05

 0

 0

 0

 3.14816855294E-05

 0

 3.14816855294E-05

 0

 0

 0

 0

 0

 0

 0

 0

 0

 0

 0

 0

 0

 0

 0

 0

 0

 0

 0

 0

 1.57408427647E-05

 1.57408427647E-05

 1.57408427647E-05

 0

 0

 0

 0

 1.57408427647E-05

 0

 0

 0

 0

 0

 0

 0

 .001196304050119

 .001196304050119

 .001196304050119

 .001196304050119

 .000912968880354

 .000283335169765

 0

 0

 0

 0

 0

 0

 0

 0

 0

 0

 0

 0

 0

 0

 0

 0

 0

 0

 0

 0

 0

 0

 0

 0

 0

 0

 0

 0

 0

 0

 0

 0

 0

 0

 0

 0

 0

 0

 0

 0

 0

 0

 0

 0

 0

 0

 0

 0

 6.925970816469E-04

 0

 0

 0

 0

 0

 0

 0

 0

 0

 0

 0

 0

 0

 0

 0

 0

 0

 0

 6.925970816469E-04

 0

 0

 0

 0

 6.925970816469E-04

 6.925970816469E-04

 .000204630955941

 0

 6.29633710589E-05

 0

 .000157408427647

 .000267594327

 0

 0

 0

 0

 0

 0

 0

 0

 0

 0

 .972122967463606

 .967133120307179

 8.657463520601E-04

 8.657463520601E-04

 0

 0

 .000125926742118

 .000220371798706

 6.29633710589E-05

 0

 0

 0

 0

 0

 0

 0

 .000409261911883

 0

 0

 0

 0

 4.72225282942E-05

 0

 0

 0

 0

 0

 0

 0

 1.259267421178E-04

 1.259267421178E-04

 0

 0

 6.29633710589E-05

 0

 6.29633710589E-05

 0

 0

 0

 0

 0

 0

 0

 0

 .000110185899353

 .000110185899353

 0

 0

 0

 3.14816855294E-05

 0

 7.87042138236E-05

 0

 0

 0

 0

 4.407435974123E-04

 4.407435974123E-04

 9.44450565883E-05

 .000346298540824

 0

 0

 0

 0

 0

 0

 0

 0

 0

 0

 0

 0

 1.3694533205298E-03

 0

 0

 0

 0

 2.833351697646E-04

 0

 0

 0

 7.87042138236E-05

 .000204630955941

 0

 0

 0

 0

 0

 0

 0

 0

 0

 0

 0

 0

 0

 0

 0

 0

 0

 0

 0

 0

 0

 0

 0

 0

 0

 0

 2.361126414703E-04

 0

 3.14816855294E-05

 0

 0

 0

 .000141667584882

 0

 0

 0

 0

 0

 0

 6.29633710589E-05

 0

 0

 0

 0

 0

 0

 0

 8.500055092949E-04

 .000173149270412

 .000456484440177

 0

 1.57408427647E-05

 .000157408427647

 0

 0

 0

 4.72225282942E-05

 0

 0

 0

 0

 0

 .000535188654

 .000535188654

 0

 0

 0

 .000220371798706

 .000314816855294

 0

 0

 0

 0

 0

 0

 0

 0

 0

 0

 0

 0

 0

 .963260872987059

 0

 0

 .963197909616

 .963197909616

 6.29633710589E-05

 0

 6.29633710589E-05

 0

 .000425002754647

 .000425002754647

 0

 0

 0

 0

 0

 0

 0

 0

 0

 0

 .000425002754647

 0

 0

 0

 0

 0

 0

 0

 0

 0

 0

 0

 0

 0

 0

 0

 0

 0

 0

 0

 0

 0

 0

 0

 0

 0

 0

 0

 0

 0

 0

 0

 0

 0

 0

 0

 0

 0

 0

 0

 0

 0

 0

 0

 0

 0

 0

 0

 0

 0

 0

 0

 0

 0

 0

 0

 0

 0

 0

 0

 0

 0

 0

 4.9898471564276E-03

 0

 0

 0

 0

 0

 0

 0

 0

 0

 0

 0

 1.5111209054164E-03

 0

 0

 1.2435265784167E-03

 4.72225282942E-05

 0

 0

 0

 0

 0

 .00105463646524

 0

 0

 6.29633710589E-05

 0

 0

 0

 0

 0

 0

 0

 0

 0

 0

 0

 0

 0

 0

 0

 7.87042138236E-05

 0

 0

 0

 0

 0

 0

 0

 0

 0

 0

 0

 0

 1.57408427647E-05

 1.57408427647E-05

 0

 9.44450565883E-05

 9.44450565883E-05

 0

 .000141667584882

 0

 .000141667584882

 1.57408427647E-05

 1.57408427647E-05

 0

 0

 0

 0

 0

 0

 0

 0

 0

 0

 0

 0

 0

 0

 0

 0

 0

 0

 0

 0

 0

 0

 0

 0

 .000236112641471

 .000236112641471

 0

 0

 0

 0

 .000236112641471

 0

 0

 0

 0

 0

 0

 0

 0

 0

 0

 0

 0

 0

 0

 0

 .000204630955941

 .000204630955941

 .000204630955941

 0

 0

 0

 0

 0

 0

 0

 0

 0

 0

 0

 0

 0

 0

 0

 0

 0

 0

 0

 0

 0

 1.4166758488294E-03

 1.4009350060647E-03

 .0013851941633

 0

 1.57408427647E-05

 0

 0

 0

 1.57408427647E-05

 1.57408427647E-05

 0

 0

 0

 0

 0

 0

 0

 0

 0

 0

 0

 0

 0

 0

 0

 0

 0

 0

 0

 1.6213068047698E-03

 1.5426025909462E-03

 .000314816855294

 4.72225282942E-05

 0

 0

 .000125926742118

 0

 .00105463646524

 7.87042138236E-05

 0

 0

 0

 3.14816855294E-05

 0

 4.72225282942E-05

 0

 0

 0

 0

 0

 0

 0

 0

 0

 0

 0

 0

 0

 0

 0

 0

 0

 3.14816855294E-05

 3.14816855294E-05

 3.14816855294E-05

 3.14816855294E-05

 0

 3.14816855294E-05

 0

 0

 0

 0

 0

 0

 0

 0

 0

 0

 0

 0

 0

 0

 0

 0

 0

 0

 0

 0

 0

 0

 0

 0

 0

 0

 0

 0

 0

 0

 0

 0

 0

 0

 0

 .000157408427647

 .000157408427647

 .000157408427647

 .000157408427647

 .000157408427647

 0

 0

 0

 0
