# Supplementary material for: First Insight into Microbiome Profiles of Myrmecophilous Beetles and Their Host, Red Wood Ant Formica polyctena (Hymenoptera: Formicidae)—A Case Study
Source: Insects. 2020 Feb 19;11(2):134. doi: 10.3390/insects11020134 (PMC7073670; doi:10.3390/insects11020134)
Supplement: Supplementary file 1 [file insects-11-00134-s001.zip › Supplementary Files/S2_Figure.zip!/Supplemental_Figure_S2/Supplemental_Figure_S2_LF2.html]

Javascript must be enabled to view this page.

magnitude

 1.00000000000037

 1.00000000000037

 0

 0

 0

 0

 0

 0

 2.990698926342E-04

 2.990698926342E-04

 8.97209677902E-05

 8.97209677902E-05

 0

 0

 8.97209677902E-05

 0

 0

 0

 0

 0

 0

 0

 0

 0

 0

 0

 0

 .000209348924844

 .000209348924844

 .000209348924844

 0

 0

 0

 0

 0

 0

 0

 0

 0

 0

 0

 0

 0

 0

 0

 0

 0

 0

 0

 0

 0

 0

 0

 0

 0

 0

 0

 0

 0

 0

 0

 0

 0

 0

 0

 0

 0

 0

 0

 0

 0

 0

 0

 0

 0

 0

 0

 0

 0

 0

 0

 0

 0

 0

 0

 0

 0

 0

 0

 0

 0

 0

 0

 6.8487005413248E-03

 0

 0

 0

 0

 0

 0

 0

 0

 0

 0

 0

 0

 0

 0

 0

 0

 0

 0

 0

 0

 0

 0

 0

 0

 0

 0

 0

 0

 0

 0

 0

 0

 0

 0

 0

 0

 0

 0

 6.7440260789029E-03

 0

 0

 0

 8.97209677902E-05

 8.97209677902E-05

 8.97209677902E-05

 0

 0

 0

 0

 0

 0

 0

 2.5271405927566E-03

 1.3308610222207E-03

 0

 1.49534946317E-05

 .000299069892634

 .000418697849687

 .000598139785268

 0

 0

 0

 0

 .000583186290636

 .000583186290636

 5.383258067414E-04

 0

 2.99069892634E-05

 0

 .000508418817478

 0

 0

 0

 0

 0

 7.47674731585E-05

 7.47674731585E-05

 0

 0

 7.47674731585E-05

 0

 0

 4.48604838951E-05

 4.48604838951E-05

 0

 0

 0

 2.99069892634E-05

 0

 2.99069892634E-05

 0

 0

 0

 0

 0

 0

 0

 0

 0

 0

 0

 0

 0

 0

 0

 0

 0

 0

 0

 0

 0

 7.626282262162E-04

 0

 0

 0

 0

 0

 0

 1.49534946317E-05

 1.49534946317E-05

 0

 0

 0

 0

 0

 0

 0

 0

 0

 0

 0

 0

 0

 0

 0

 0

 0

 0

 0

 0

 0

 1.345814516854E-04

 .000104674462422

 0

 0

 1.49534946317E-05

 0

 0

 0

 0

 0

 0

 0

 0

 1.49534946317E-05

 0

 0

 0

 0

 0

 0

 0

 0

 0

 0

 6.130932798991E-04

 0

 0

 1.49534946317E-05

 .000568232796004

 0

 0

 2.99069892634E-05

 0

 0

 0

 0

 0

 0

 0

 0

 0

 0

 0

 0

 0

 1.7645123665434E-03

 2.093489248434E-04

 0

 0

 0

 2.99069892634E-05

 .00017944193558

 .0015551634417

 .0015551634417

 0

 0

 0

 0

 .000164488440949

 .000164488440949

 0

 0

 0

 0

 0

 .000164488440949

 0

 .00119627957054

 .00119627957054

 0

 0

 .00119627957054

 .000164488440949

 .000164488440949

 0

 0

 .000164488440949

 0

 0

 0

 0

 0

 0

 0

 0

 0

 0

 1.046744624219E-04

 7.47674731585E-05

 0

 0

 7.47674731585E-05

 0

 0

 0

 7.47674731585E-05

 2.99069892634E-05

 0

 0

 1.49534946317E-05

 1.49534946317E-05

 0

 1.49534946317E-05

 0

 1.49534946317E-05

 0

 0

 0

 0

 0

 0

 0

 0

 0

 0

 0

 0

 0

 0

 0

 0

 0

 0

 0

 0

 1.046744624219E-04

 1.046744624219E-04

 0

 0

 0

 0

 0

 0

 0

 0

 0

 0

 0

 0

 0

 0

 0

 0

 0

 0

 0

 0

 0

 0

 0

 0

 0

 0

 0

 0

 0

 0

 0

 0

 0

 0

 0

 0

 0

 0

 0

 0

 0

 0

 8.97209677902E-05

 0

 0

 4.48604838951E-05

 0

 4.48604838951E-05

 0

 0

 4.48604838951E-05

 0

 4.48604838951E-05

 0

 0

 1.49534946317E-05

 0

 0

 0

 0

 1.49534946317E-05

 0

 0

 0

 1.49534946317E-05

 0

 0

 0

 0

 0

 0

 0

 0

 0

 0

 0

 0

 0

 0

 0

 0

 0

 0

 0

 0

 0

 0

 0

 0

 0

 0

 0

 0

 0

 0

 0

 0

 0

 0

 0

 0

 0

 0

 0

 0

 0

 0

 0

 0

 0

 0

 0

 0

 0

 0

 0

 0

 0

 0

 0

 0

 0

 0

 0

 0

 0

 0

 0

 0

 0

 0

 0

 0

 0

 0

 0

 0

 0

 0

 0

 0

 0

 0

 0

 0

 0

 0

 0

 0

 0

 0

 0

 0

 0

 0

 0

 0

 0

 1.49534946317E-05

 1.49534946317E-05

 1.49534946317E-05

 1.49534946317E-05

 1.49534946317E-05

 0

 0

 0

 0

 0

 0

 0

 0

 0

 0

 0

 0

 0

 0

 0

 0

 0

 0

 0

 0

 0

 0

 0

 0

 0

 0

 1.4504889792744E-03

 1.4504889792744E-03

 9.570236564287E-04

 1.49534946317E-05

 1.49534946317E-05

 0

 0

 0

 0

 0

 0

 0

 0

 0

 0

 0

 0

 0

 0

 0

 0

 0

 0

 0

 0

 0

 0

 0

 0

 0

 0

 0

 0

 0

 0

 .000942070161797

 0

 0

 .000942070161797

 0

 0

 4.934653228457E-04

 0

 0

 0

 0

 0

 0

 0

 0

 0

 0

 0

 0

 0

 .000104674462422

 .000104674462422

 3.887908604237E-04

 1.49534946317E-05

 .000373837365792

 0

 0

 0

 0

 0

 0

 0

 0

 0

 0

 0

 0

 0

 0

 0

 0

 0

 0

 0

 0

 0

 0

 0

 0

 0

 0

 0

 0

 0

 0

 0

 0

 0

 0

 0

 0

 0

 0

 0

 0

 0

 0

 0

 0

 0

 0

 0

 0

 0

 0

 0

 0

 0

 0

 0

 0

 0

 0

 0

 0

 0

 0

 0

 0

 0

 0

 0

 0

 0

 0

 0

 0

 0

 0

 0

 0

 0

 1.49534946317E-05

 0

 0

 0

 0

 0

 0

 0

 0

 0

 0

 0

 0

 1.49534946317E-05

 1.49534946317E-05

 1.49534946317E-05

 1.49534946317E-05

 0

 0

 0

 0

 0

 0

 0

 0

 0

 0

 0

 0

 0

 0

 0

 0

 0

 1.943954302122E-04

 0

 0

 0

 0

 0

 0

 0

 0

 0

 0

 0

 0

 0

 0

 0

 0

 0

 0

 1.943954302122E-04

 0

 0

 0

 0

 1.943954302122E-04

 1.943954302122E-04

 .000104674462422

 0

 0

 0

 8.97209677902E-05

 0

 0

 0

 0

 0

 0

 0

 0

 0

 0

 0

 .99107276370524

 .988919460478275

 0

 0

 0

 0

 0

 0

 0

 0

 0

 0

 0

 0

 0

 0

 0

 0

 0

 0

 0

 0

 0

 0

 0

 0

 0

 0

 0

 .000284116398002

 .000284116398002

 0

 0

 .000284116398002

 0

 0

 0

 0

 0

 0

 0

 0

 0

 0

 0

 0

 0

 0

 0

 0

 0

 0

 0

 0

 0

 0

 1.49534946317E-05

 1.49534946317E-05

 0

 1.49534946317E-05

 0

 0

 0

 0

 0

 0

 0

 0

 0

 0

 0

 0

 4.336513443197E-04

 0

 0

 0

 0

 .000104674462422

 0

 0

 0

 0

 .000104674462422

 0

 0

 0

 0

 0

 0

 0

 0

 0

 0

 0

 0

 0

 0

 0

 0

 0

 0

 0

 0

 0

 0

 0

 0

 0

 0

 .000149534946317

 0

 7.47674731585E-05

 0

 0

 0

 7.47674731585E-05

 0

 0

 0

 0

 0

 0

 0

 0

 0

 0

 0

 0

 0

 0

 1.794419355807E-04

 1.49534946317E-05

 .000164488440949

 0

 0

 0

 0

 0

 0

 0

 0

 0

 0

 0

 0

 2.542094087386E-04

 2.542094087386E-04

 0

 0

 0

 5.98139785268E-05

 .000134581451685

 0

 0

 0

 1.49534946317E-05

 4.48604838951E-05

 0

 0

 0

 0

 0

 0

 0

 0

 .987827855370161

 0

 0

 .987468971499

 .987468971499

 .000358883871161

 0

 .000358883871161

 0

 .000104674462422

 .000104674462422

 0

 0

 0

 0

 0

 0

 0

 0

 0

 0

 .000104674462422

 0

 0

 0

 0

 0

 0

 0

 0

 0

 0

 0

 0

 0

 1.345814516853E-04

 0

 0

 0

 8.97209677902E-05

 0

 0

 0

 8.97209677902E-05

 0

 8.97209677902E-05

 0

 0

 0

 0

 0

 0

 0

 0

 0

 0

 0

 0

 0

 0

 0

 0

 0

 0

 0

 0

 0

 0

 0

 0

 0

 0

 0

 0

 0

 0

 0

 4.48604838951E-05

 4.48604838951E-05

 4.48604838951E-05

 0

 0

 0

 0

 2.0187217752796E-03

 0

 0

 0

 0

 0

 0

 0

 0

 0

 0

 0

 .000299069892634

 0

 0

 1.943954302121E-04

 2.99069892634E-05

 0

 0

 0

 0

 0

 0

 0

 0

 8.97209677902E-05

 0

 0

 0

 0

 0

 0

 0

 0

 0

 0

 0

 0

 0

 0

 0

 5.98139785268E-05

 0

 0

 0

 0

 0

 1.49534946317E-05

 0

 0

 0

 0

 0

 0

 0

 0

 0

 4.48604838951E-05

 0

 4.48604838951E-05

 0

 0

 0

 0

 0

 0

 0

 0

 0

 5.98139785268E-05

 0

 0

 0

 5.98139785268E-05

 0

 0

 0

 0

 0

 0

 0

 0

 0

 0

 0

 0

 0

 0

 0

 5.682327960043E-04

 5.682327960043E-04

 .000433651344319

 0

 0

 0

 0

 0

 2.99069892634E-05

 0

 5.98139785268E-05

 0

 0

 0

 0

 0

 2.99069892634E-05

 0

 0

 0

 1.49534946317E-05

 0

 0

 0

 0

 0

 0

 0

 0

 0

 0

 0

 0

 0

 0

 0

 0

 0

 0

 0

 0

 0

 0

 5.98139785268E-05

 5.98139785268E-05

 5.98139785268E-05

 7.626282262168E-04

 7.626282262168E-04

 .00070281424769

 5.98139785268E-05

 0

 0

 0

 0

 0

 0

 0

 0

 0

 0

 0

 0

 0

 0

 0

 0

 0

 0

 0

 0

 0

 0

 0

 0

 0

 3.289768818977E-04

 1.644884409487E-04

 0

 4.48604838951E-05

 2.99069892634E-05

 0

 1.49534946317E-05

 0

 7.47674731585E-05

 .000164488440949

 0

 0

 0

 0

 0

 .000164488440949

 0

 0

 0

 0

 0

 0

 0

 0

 0

 0

 0

 0

 0

 0

 0

 0

 0

 0

 0

 0

 0

 0

 0

 0

 0

 0

 0

 0

 0

 0

 0

 0

 0

 0

 0

 0

 0

 0

 0

 0

 0

 0

 0

 0

 0

 0

 0

 0

 0

 0

 0

 0

 0

 0

 0

 0

 0

 0

 0

 0

 0

 0

 0

 0

 0

 0

 0
