# Supplementary material for: First Insight into Microbiome Profiles of Myrmecophilous Beetles and Their Host, Red Wood Ant Formica polyctena (Hymenoptera: Formicidae)—A Case Study
Source: Insects. 2020 Feb 19;11(2):134. doi: 10.3390/insects11020134 (PMC7073670; doi:10.3390/insects11020134)
Supplement: Supplementary file 1 [file insects-11-00134-s001.zip › Supplementary Files/S2_Figure.zip!/Supplemental_Figure_S2/Supplemental_Figure_S2_LF3.html]

Javascript must be enabled to view this page.

magnitude

 .999999999999483

 .999999999999483

 1.0138199669172E-03

 1.0138199669172E-03

 1.0138199669172E-03

 1.0138199669172E-03

 3.55726304182E-05

 .000978247336499

 1.21480532878012E-02

 1.14721733098562E-02

 1.08140796471202E-02

 1.08140796471202E-02

 .000124504206464

 0

 .00983583231062

 3.55726304182E-05

 0

 .000693666293154

 0

 0

 .000124504206464

 0

 0

 0

 0

 0

 0

 .000658093662736

 .000658093662736

 .000658093662736

 0

 0

 0

 0

 0

 0

 0

 0

 0

 0

 0

 0

 0

 0

 0

 0

 0

 0

 0

 0

 0

 0

 0

 0

 0

 0

 0

 .000675879977945

 0

 0

 0

 0

 .000675879977945

 0

 0

 0

 0

 .000675879977945

 .000675879977945

 0

 0

 0

 0

 0

 0

 0

 0

 0

 0

 0

 0

 0

 0

 0

 0

 0

 0

 0

 0

 0

 0

 0

 0

 .658396030094

 0

 0

 0

 0

 0

 0

 0

 0

 0

 0

 0

 0

 0

 0

 0

 0

 0

 0

 0

 0

 0

 0

 0

 0

 0

 0

 0

 0

 0

 0

 0

 0

 0

 0

 0

 0

 0

 0

 .658093662735446

 0

 0

 0

 .002899169379084

 .002899169379084

 .000693666293154

 .00220550308593

 0

 0

 0

 3.55726304182E-05

 3.55726304182E-05

 3.55726304182E-05

 .150561158244785

 .08215498995074

 0

 .00154740942319

 .00421535670455

 .0300055137577

 .0463867100653

 0

 0

 0

 0

 .0278711559326

 .0278711559326

 4.05350123614454E-02

 8.89315760454E-05

 0

 0

 .0404460807854

 0

 0

 0

 0

 0

 0

 0

 0

 0

 .002774665172616

 0

 0

 .0005869484019

 .0005869484019

 0

 0

 0

 .00152962310798

 0

 .00152962310798

 0

 0

 0

 0

 0

 .000658093662736

 .000658093662736

 0

 0

 0

 0

 0

 0

 0

 0

 0

 0

 0

 0

 0

 0

 7.06828166408289E-02

 .000373512619391

 .000373512619391

 .000818170499618

 .000818170499618

 0

 0

 8.89315760454E-05

 8.89315760454E-05

 .00981804599541

 0

 0

 0

 0

 .00981804599541

 0

 0

 0

 0

 0

 0

 0

 8.8753712893291E-03

 .00885758497412

 0

 1.77863152091E-05

 0

 0

 0

 0

 2.00273909253791E-02

 .000160076836882

 1.77863152091E-05

 1.77863152091E-05

 .00225886203155

 1.77863152091E-05

 0

 3.55726304182E-05

 0

 1.77863152091E-05

 .0104583533429

 1.77863152091E-05

 .000231222097718

 .00670544083382

 0

 0

 1.77863152091E-05

 0

 0

 0

 0

 5.33589456272E-05

 0

 1.77863152091E-05

 3.06458211052381E-02

 0

 .000213435782509

 .00433986091102

 .0260747380965

 0

 0

 0

 1.77863152091E-05

 3.55726304182E-05

 3.55726304182E-05

 0

 0

 0

 0

 0

 0

 0

 0

 0

 0

 .220247941233998

 3.4861177809782E-03

 .00167191362965

 1.77863152091E-05

 0

 1.77863152091E-05

 .00177863152091

 .21676182345302

 .214378457215

 .00238336623802

 0

 0

 0

 2.86359674865691E-02

 2.86359674865691E-02

 1.77863152091E-05

 .0147804279387

 .00419757038934

 0

 0

 .00964018284332

 0

 .180815680415209

 .180815680415209

 0

 1.77863152091E-05

 .1807978941

 1.4406915319372E-03

 1.4406915319372E-03

 5.33589456272E-05

 0

 .00138733258631

 0

 0

 0

 0

 0

 0

 0

 0

 0

 0

 .000302367358554

 0

 0

 0

 0

 0

 0

 0

 0

 .000302367358554

 0

 0

 .000302367358554

 .000302367358554

 0

 0

 0

 0

 0

 0

 0

 0

 0

 0

 0

 0

 0

 0

 0

 0

 0

 0

 0

 0

 0

 0

 0

 0

 .012468206961559

 .012468206961559

 0

 0

 0

 0

 0

 0

 0

 0

 0

 0

 0

 .009835832310619

 0

 0

 .009835832310619

 0

 .00400192092204

 0

 0

 0

 0

 0

 0

 0

 0

 0

 .000978247336499

 0

 0

 0

 .00485566405208

 0

 0

 0

 0

 0

 0

 0

 0

 0

 0

 0

 .00263237465094

 0

 0

 0

 0

 0

 0

 0

 .00263237465094

 0

 .00263237465094

 0

 0

 0

 0

 0

 0

 0

 0

 0

 0

 0

 0

 0

 0

 0

 0

 0

 0

 0

 0

 0

 0

 0

 0

 0

 0

 0

 .000622521032318

 0

 0

 0

 0

 0

 0

 0

 0

 0

 0

 0

 0

 0

 0

 0

 0

 0

 0

 .000622521032318

 0

 0

 0

 0

 0

 .000622521032318

 .000622521032318

 .000622521032318

 0

 0

 0

 0

 0

 0

 0

 0

 0

 0

 0

 0

 0

 0

 0

 0

 0

 0

 0

 0

 0

 0

 0

 0

 0

 0

 0

 0

 0

 0

 0

 0

 0

 0

 0

 0

 0

 0

 0

 0

 0

 0

 0

 0

 0

 0

 0

 0

 0

 0

 0

 0

 0

 0

 0

 0

 0

 0

 0

 0

 0

 0

 0

 0

 0

 0

 0

 0

 0

 0

 0

 0

 0

 0

 0

 0

 0

 0

 0

 0

 0

 .116678227771556

 .111911495295522

 .036853245113214

 .000160076836882

 .000160076836882

 0

 0

 0

 0

 0

 0

 0

 0

 0

 0

 .00695444924675

 .00695444924675

 0

 0

 0

 .000924888390872

 0

 0

 0

 .000924888390872

 0

 0

 0

 .00177863152091

 0

 0

 .00177863152091

 0

 0

 0

 .0270351991178

 0

 0

 .0270351991178

 0

 0

 7.50582501823082E-02

 0

 0

 .00096046102129

 0

 .00096046102129

 0

 0

 0

 1.1561104885882E-03

 3.55726304182E-05

 .00112053785817

 .00917773864788

 .00917773864788

 0

 0

 .06376394002455

 .00499795457375

 .0587659854508

 1.1205378581682E-03

 1.1205378581682E-03

 0

 0

 0

 0

 0

 0

 0

 0

 0

 0

 0

 0

 0

 0

 0

 0

 0

 .00108496522775

 0

 0

 0

 .00108496522775

 0

 0

 0

 0

 0

 3.55726304182E-05

 0

 3.55726304182E-05

 0

 0

 0

 0

 0

 0

 0

 0

 0

 0

 3.6461946178654E-03

 3.6461946178654E-03

 3.6461946178654E-03

 5.33589456272E-05

 .00163634099924

 0

 3.55726304182E-05

 .00192092204258

 0

 0

 0

 0

 0

 0

 0

 0

 0

 0

 0

 0

 0

 0

 0

 0

 0

 0

 0

 0

 0

 0

 0

 0

 0

 0

 0

 0

 0

 0

 0

 0

 0

 0

 0

 0

 0

 0

 0

 0

 0

 0

 0

 0

 0

 0

 0

 0

 0

 0

 0

 0

 0

 0

 0

 0

 0

 0

 0

 0

 0

 0

 0

 0

 0

 0

 0

 0

 0

 0

 0

 0

 0

 0

 0

 0

 0

 0

 0

 0

 0

 0

 0

 0

 0

 0

 0

 0

 0

 0

 0

 0

 0

 0

 0

 0

 0

 0

 0

 0

 0

 0

 0

 .106095370222091

 3.96279102858311E-02

 .000266794728136

 .000266794728136

 0

 0

 0

 0

 0

 0

 0

 0

 0

 0

 0

 0

 0

 0

 0

 0

 0

 .000266794728136

 0

 0

 0

 0

 0

 0

 0

 .00498016825854

 .00096046102129

 0

 0

 .00096046102129

 0

 0

 0

 .00401970723725

 0

 0

 .00401970723725

 0

 0

 0

 4.624441954362E-04

 4.624441954362E-04

 0

 0

 3.55726304182E-05

 0

 .000426871565018

 0

 0

 0

 0

 0

 .00771926080074

 .00771926080074

 0

 .00771926080074

 0

 0

 0

 0

 0

 0

 0

 0

 0

 0

 0

 0

 7.6125429094852E-03

 0

 0

 0

 0

 0

 0

 0

 0

 0

 0

 0

 0

 0

 0

 0

 0

 0

 0

 0

 0

 0

 0

 0

 0

 0

 0

 0

 0

 0

 0

 0

 0

 0

 0

 0

 0

 0

 0

 0

 0

 0

 0

 0

 0

 0

 0

 0

 0

 0

 0

 0

 0

 0

 0

 0

 0

 0

 7.6125429094852E-03

 .0037173398787

 .00245451149885

 0

 .000711452608363

 0

 0

 .000675879977945

 5.33589456272E-05

 0

 0

 0

 0

 0

 0

 1.13476691033964E-02

 1.13476691033964E-02

 0

 0

 3.55726304182E-05

 .00389520303079

 .00738132081177

 0

 0

 3.55726304182E-05

 0

 0

 0

 0

 0

 0

 0

 0

 0

 0

 .000942674706081

 0

 0

 .000835956814827

 .000835956814827

 .000106717891254

 0

 .000106717891254

 0

 6.2963555840163E-03

 6.2963555840163E-03

 .0015651957384

 0

 0

 0

 0

 0

 0

 0

 0

 .00158298205361

 .00307703253117

 0

 1.77863152091E-05

 5.33589456272E-05

 0

 0

 0

 0

 0

 0

 0

 0

 0

 0

 0

 0

 0

 0

 0

 0

 0

 0

 0

 0

 0

 0

 0

 0

 0

 0

 0

 0

 0

 0

 0

 0

 0

 0

 0

 0

 0

 0

 0

 0

 0

 0

 0

 0

 0

 0

 0

 0

 0

 0

 0

 0

 0

 0

 0

 0

 0

 0

 0

 6.64674599362601E-02

 0

 0

 0

 .000533589456272

 .000533589456272

 .000533589456272

 0

 0

 0

 0

 0

 2.82980274976202E-02

 0

 0

 2.56834391618902E-02

 0

 0

 0

 0

 .0210056382619

 0

 0

 0

 0

 .00389520303079

 0

 0

 0

 0

 0

 0

 3.55726304182E-05

 .0001956494673

 0

 0

 0

 0

 0

 .000177863152091

 0

 0

 0

 0

 0

 0

 0

 0

 .000373512619391

 0

 0

 0

 0

 0

 0

 0

 0

 0

 0

 0

 .00261458833573

 0

 .00261458833573

 0

 0

 0

 0

 0

 0

 0

 0

 0

 0

 0

 0

 0

 0

 0

 0

 0

 0

 0

 0

 0

 .0003912989346

 .0003912989346

 0

 .0003912989346

 0

 8.9998754957909E-03

 8.9998754957909E-03

 .000320153673763

 3.55726304182E-05

 1.77863152091E-05

 0

 .00592284296462

 3.55726304182E-05

 .000711452608363

 0

 .000177863152091

 0

 0

 0

 0

 .00154740942319

 8.89315760454E-05

 0

 0

 0

 .000142290521673

 0

 0

 0

 0

 0

 0

 0

 0

 0

 0

 0

 0

 0

 0

 0

 0

 0

 0

 0

 0

 0

 0

 0

 0

 0

 .01880013517595

 .014976077406

 .0147804279387

 0

 .0001956494673

 0

 0

 0

 .00382405776995

 .00382405776995

 0

 0

 0

 0

 0

 0

 0

 0

 0

 0

 0

 0

 0

 0

 0

 0

 0

 0

 0

 .009444533376027

 .007914910268044

 0

 .00346833146577

 .00375291250912

 0

 0

 0

 .000693666293154

 .001529623107983

 0

 .00138733258631

 0

 .000142290521673

 0

 0

 0

 0

 0

 0

 0

 0

 0

 0

 0

 0

 0

 0

 .0913327285986

 .0913327285986

 .0913327285986

 .0913327285986

 .0913327285986

 .00124504206464

 .00124504206464

 .00124504206464

 .00124504206464

 0

 .00124504206464

 0

 0

 0

 0

 0

 0

 0

 0

 0

 0

 0

 0

 0

 0

 0

 0

 0

 0

 0

 0

 0

 0

 0

 0

 0

 0

 0

 0

 0

 0

 0

 0

 0

 0

 0

 0

 0

 0

 0

 0

 0

 0

 0

 0
