# Supplementary material for: First Insight into Microbiome Profiles of Myrmecophilous Beetles and Their Host, Red Wood Ant Formica polyctena (Hymenoptera: Formicidae)—A Case Study
Source: Insects. 2020 Feb 19;11(2):134. doi: 10.3390/insects11020134 (PMC7073670; doi:10.3390/insects11020134)
Supplement: Supplementary file 1 [file insects-11-00134-s001.zip › Supplementary Files/S2_Figure.zip!/Supplemental_Figure_S2/Supplemental_Figure_S2_MA1.html]

Javascript must be enabled to view this page.

magnitude

 .999999999999794

 .999999999999794

 0

 0

 0

 0

 0

 0

 3.881721657721E-04

 3.881721657721E-04

 1.826692544811E-04

 1.826692544811E-04

 6.85009704304E-05

 0

 4.56673136203E-05

 0

 2.28336568101E-05

 0

 0

 4.56673136203E-05

 0

 0

 0

 0

 0

 0

 0

 .000205502911291

 .000205502911291

 .000205502911291

 0

 0

 0

 0

 0

 0

 0

 0

 0

 0

 0

 0

 0

 0

 0

 0

 0

 0

 0

 0

 0

 0

 0

 0

 0

 0

 0

 0

 0

 0

 0

 0

 0

 0

 0

 0

 0

 0

 0

 0

 0

 0

 0

 0

 0

 0

 0

 0

 0

 0

 0

 0

 0

 0

 0

 0

 0

 0

 0

 0

 0

 0

 0

 .109989724854516

 0

 0

 0

 0

 6.85009704304E-05

 0

 0

 0

 0

 2.28336568101E-05

 2.28336568101E-05

 2.28336568101E-05

 0

 0

 0

 0

 0

 0

 0

 0

 0

 0

 0

 0

 0

 0

 0

 0

 0

 4.56673136203E-05

 0

 0

 0

 0

 4.56673136203E-05

 4.56673136203E-05

 0

 0

 .109624386345554

 0

 0

 0

 0

 0

 0

 0

 0

 0

 0

 2.28336568101E-05

 2.28336568101E-05

 2.28336568101E-05

 8.70418997603182E-02

 1.826692544814E-04

 0

 0

 .000114168284051

 0

 6.85009704304E-05

 0

 0

 0

 0

 .00230619933782

 .00230619933782

 3.13277771435468E-02

 0

 .0282909007878

 .000936179929216

 .00196369448567

 2.28336568101E-05

 2.28336568101E-05

 9.13346272406E-05

 .00196369448567

 .00196369448567

 .0512615595388

 .0512615595388

 0

 0

 2.2148647105841E-03

 0

 0

 2.28336568101E-05

 2.28336568101E-05

 0

 0

 0

 .000274003881722

 0

 .000274003881722

 0

 .000274003881722

 .000274003881722

 0

 0

 .00164402329033

 .00164402329033

 0

 0

 0

 0

 0

 0

 0

 0

 0

 0

 0

 0

 0

 0

 8.3799520493209E-03

 0

 0

 0

 0

 0

 0

 .000251170224912

 .000251170224912

 7.7406096586363E-03

 0

 0

 0

 .000182669254481

 .00666742778856

 .000844845301975

 0

 4.56673136203E-05

 0

 0

 0

 0

 2.28336568101E-05

 0

 0

 0

 0

 0

 2.28336568101E-05

 0

 1.141682840506E-04

 4.56673136203E-05

 0

 0

 0

 0

 0

 0

 0

 0

 2.28336568101E-05

 0

 2.28336568101E-05

 2.28336568101E-05

 0

 0

 0

 0

 0

 0

 0

 0

 0

 0

 2.511702249119E-04

 0

 .000114168284051

 4.56673136203E-05

 4.56673136203E-05

 0

 0

 4.56673136203E-05

 0

 0

 0

 0

 0

 .000114168284051

 .000114168284051

 0

 .000114168284051

 0

 0

 0

 0

 3.1053773261806E-03

 2.1235300833446E-03

 .000730677017924

 0

 0

 .00130151843818

 9.13346272406E-05

 .000981847242836

 .000296837538532

 0

 .000228336568101

 .000456673136203

 0

 1.6896906039543E-03

 1.6896906039543E-03

 0

 .000502340449823

 .000114168284051

 4.56673136203E-05

 0

 .00102751455646

 0

 6.2335883091701E-03

 6.2335883091701E-03

 2.28336568101E-05

 0

 .00621075465236

 8.220116451645E-04

 8.220116451645E-04

 2.28336568101E-05

 6.85009704304E-05

 .000730677017924

 0

 0

 0

 0

 0

 0

 0

 0

 0

 0

 2.968375385315E-04

 0

 0

 0

 0

 0

 0

 0

 0

 2.968375385315E-04

 0

 0

 2.283365681011E-04

 .000205502911291

 2.28336568101E-05

 6.85009704304E-05

 0

 6.85009704304E-05

 0

 0

 0

 0

 0

 0

 0

 0

 0

 0

 0

 0

 0

 0

 0

 0

 0

 0

 0

 0

 5.12843931955228E-02

 5.12843931955228E-02

 2.28336568101E-05

 0

 0

 0

 0

 2.28336568101E-05

 0

 0

 2.28336568101E-05

 0

 0

 5.11702249114721E-02

 0

 0

 5.11702249114721E-02

 0

 .000365338508962

 0

 0

 0

 0

 0

 0

 .0507820527457

 0

 0

 0

 0

 2.28336568101E-05

 0

 0

 0

 0

 0

 0

 0

 0

 0

 0

 0

 0

 0

 9.13346272406E-05

 0

 0

 0

 0

 0

 0

 0

 9.13346272406E-05

 0

 9.13346272406E-05

 0

 0

 0

 0

 0

 0

 0

 0

 0

 0

 0

 0

 0

 0

 0

 0

 0

 0

 0

 0

 0

 0

 0

 0

 0

 0

 0

 .000182669254481

 0

 0

 0

 0

 0

 0

 0

 0

 0

 0

 0

 0

 0

 0

 0

 0

 0

 0

 .000182669254481

 0

 0

 0

 0

 0

 .000182669254481

 .000182669254481

 0

 .000182669254481

 0

 0

 0

 0

 0

 0

 0

 0

 0

 0

 0

 0

 0

 0

 0

 0

 0

 0

 0

 0

 0

 0

 0

 0

 0

 0

 0

 0

 0

 0

 0

 0

 0

 0

 0

 0

 0

 0

 0

 0

 0

 0

 0

 0

 0

 0

 0

 0

 0

 0

 0

 0

 0

 0

 0

 0

 0

 0

 0

 0

 0

 0

 0

 0

 0

 0

 0

 0

 0

 0

 0

 0

 0

 0

 0

 0

 0

 0

 0

 0

 2.8770407580762E-03

 2.8542071012661E-03

 1.598355976709E-04

 0

 0

 0

 0

 0

 0

 0

 0

 0

 0

 2.28336568101E-05

 2.28336568101E-05

 0

 0

 0

 0

 0

 6.85009704304E-05

 0

 0

 0

 6.85009704304E-05

 0

 0

 0

 0

 0

 0

 0

 0

 0

 0

 6.85009704304E-05

 0

 0

 6.85009704304E-05

 0

 0

 2.6943715035952E-03

 0

 0

 0

 0

 0

 0

 0

 0

 2.28336568101E-05

 0

 2.28336568101E-05

 .000776344331545

 .000776344331545

 2.28336568101E-05

 2.28336568101E-05

 .00187235985843

 .00187235985843

 0

 2.28336568101E-05

 2.28336568101E-05

 0

 0

 0

 0

 0

 0

 0

 0

 2.28336568101E-05

 2.28336568101E-05

 0

 0

 0

 0

 0

 0

 0

 0

 0

 0

 0

 0

 0

 0

 0

 0

 0

 0

 0

 0

 0

 0

 0

 0

 0

 0

 0

 0

 0

 0

 0

 0

 0

 0

 0

 0

 0

 0

 0

 0

 0

 0

 0

 0

 0

 0

 0

 0

 0

 0

 0

 0

 0

 0

 0

 0

 0

 0

 0

 0

 0

 0

 0

 0

 0

 4.110058225826E-04

 0

 0

 0

 0

 0

 0

 0

 0

 0

 0

 0

 0

 4.110058225826E-04

 4.110058225826E-04

 0

 0

 0

 0

 0

 0

 0

 0

 0

 0

 0

 .000319671195342

 .000319671195342

 0

 0

 0

 0

 9.13346272406E-05

 9.13346272406E-05

 4.566731362025E-04

 0

 0

 0

 0

 0

 0

 0

 0

 0

 0

 0

 0

 0

 0

 0

 0

 0

 0

 4.566731362025E-04

 2.28336568101E-05

 2.28336568101E-05

 0

 2.28336568101E-05

 4.338394793924E-04

 4.338394793924E-04

 2.28336568101E-05

 .000205502911291

 0

 4.56673136203E-05

 .000159835597671

 0

 0

 0

 0

 0

 0

 0

 0

 0

 0

 0

 .834410320812641

 .378947368420996

 2.28336568101E-05

 2.28336568101E-05

 0

 0

 0

 0

 0

 0

 0

 0

 0

 0

 0

 0

 0

 0

 0

 0

 0

 0

 2.28336568101E-05

 0

 0

 0

 0

 0

 0

 2.28336568101E-05

 2.28336568101E-05

 0

 0

 0

 0

 2.28336568101E-05

 0

 0

 0

 0

 0

 0

 0

 0

 0

 0

 0

 0

 0

 0

 0

 0

 0

 0

 0

 0

 2.28336568101E-05

 2.28336568101E-05

 0

 2.28336568101E-05

 0

 0

 0

 0

 0

 0

 0

 0

 0

 0

 0

 0

 3.57803402215059E-02

 0

 0

 0

 0

 6.85009704304E-05

 0

 4.56673136203E-05

 0

 0

 2.28336568101E-05

 0

 0

 0

 0

 0

 0

 0

 0

 0

 0

 0

 0

 0

 0

 0

 0

 0

 0

 0

 0

 4.56673136203E-05

 4.56673136203E-05

 0

 0

 .0337481447654

 .0337481447654

 6.393423906841E-04

 9.13346272406E-05

 .000137001940861

 0

 0

 0

 .000319671195342

 0

 0

 0

 0

 0

 0

 6.85009704304E-05

 2.28336568101E-05

 0

 0

 0

 0

 0

 0

 1.2786847813711E-03

 .000137001940861

 .0011188491837

 0

 0

 0

 2.28336568101E-05

 0

 0

 0

 0

 0

 0

 0

 0

 2.28336568101E-05

 2.28336568101E-05

 0

 0

 0

 0

 2.28336568101E-05

 0

 0

 0

 0

 0

 0

 0

 0

 0

 0

 0

 0

 0

 .3413860029683

 0

 0

 .314236785021

 .314236785021

 .0271492179473

 0

 .0271492179473

 0

 1.6896906039501E-03

 1.6896906039501E-03

 0

 0

 0

 0

 0

 0

 2.28336568101E-05

 0

 0

 0

 .00166685694714

 0

 0

 0

 0

 0

 0

 0

 0

 0

 0

 0

 0

 0

 6.85009704304E-05

 0

 0

 0

 0

 0

 0

 0

 0

 0

 0

 0

 0

 0

 6.85009704304E-05

 0

 0

 0

 0

 0

 0

 0

 0

 0

 0

 0

 0

 0

 0

 0

 0

 0

 4.56673136203E-05

 4.56673136203E-05

 0

 0

 2.28336568101E-05

 2.28336568101E-05

 0

 0

 0

 0

 0

 0

 0

 0

 0

 0

 0

 .455394451421214

 0

 0

 0

 0

 0

 0

 0

 0

 0

 0

 0

 6.5760931613212E-03

 0

 0

 6.5760931613212E-03

 .000114168284051

 0

 0

 .00641625756365

 0

 0

 2.28336568101E-05

 0

 0

 0

 0

 0

 0

 0

 0

 0

 0

 2.28336568101E-05

 0

 0

 0

 0

 0

 0

 0

 0

 0

 0

 0

 0

 0

 0

 0

 0

 0

 0

 0

 0

 0

 0

 0

 0

 0

 0

 0

 0

 0

 0

 0

 0

 0

 0

 0

 0

 0

 0

 0

 0

 0

 0

 0

 0

 0

 0

 0

 0

 0

 0

 2.28336568101E-05

 2.28336568101E-05

 0

 2.28336568101E-05

 0

 .35440118735

 .35440118735

 0

 0

 0

 0

 0

 0

 0

 0

 0

 0

 0

 0

 0

 0

 0

 0

 0

 0

 .35440118735

 0

 0

 0

 0

 0

 0

 0

 0

 0

 0

 0

 0

 0

 0

 0

 0

 0

 0

 0

 0

 0

 0

 0

 0

 0

 0

 0

 0

 0

 0

 0

 0

 0

 0

 0

 0

 0

 0

 0

 0

 0

 0

 0

 0

 0

 0

 0

 0

 0

 0

 0

 0

 0

 0

 9.43943372530824E-02

 3.881721657723E-04

 0

 9.13346272406E-05

 2.28336568101E-05

 0

 .000182669254481

 0

 9.13346272406E-05

 9.40061650873101E-02

 0

 0

 .0939833314305

 2.28336568101E-05

 0

 0

 0

 0

 0

 0

 0

 0

 0

 0

 0

 0

 0

 0

 0

 0

 0

 0

 0

 0

 0

 0

 0

 0

 0

 0

 0

 0

 0

 0

 0

 0

 0

 0

 0

 0

 0

 0

 0

 0

 0

 0

 0

 0

 0

 0

 0

 0

 0

 0

 0

 0

 0

 0

 0

 0

 0

 0

 0

 0

 0

 0

 0

 0

 0

 0

 0

 0

 0
