# Supplementary material for: First Insight into Microbiome Profiles of Myrmecophilous Beetles and Their Host, Red Wood Ant Formica polyctena (Hymenoptera: Formicidae)—A Case Study
Source: Insects. 2020 Feb 19;11(2):134. doi: 10.3390/insects11020134 (PMC7073670; doi:10.3390/insects11020134)
Supplement: Supplementary file 1 [file insects-11-00134-s001.zip › Supplementary Files/S2_Figure.zip!/Supplemental_Figure_S2/Supplemental_Figure_S2_MA2.html]

Javascript must be enabled to view this page.

magnitude

 1.00000000000001

 1.00000000000001

 3.58840943752E-05

 3.58840943752E-05

 3.58840943752E-05

 3.58840943752E-05

 0

 3.58840943752E-05

 7.535659818788E-04

 7.535659818788E-04

 5.741455100028E-04

 5.741455100028E-04

 .000143536377501

 0

 .000251188660626

 0

 5.38261415628E-05

 0

 0

 0

 .000125594330313

 0

 0

 0

 0

 0

 0

 .000179420471876

 .000179420471876

 .000179420471876

 0

 0

 0

 0

 0

 0

 0

 0

 0

 0

 0

 0

 0

 0

 0

 0

 0

 0

 0

 0

 0

 0

 0

 0

 0

 0

 0

 0

 0

 0

 0

 0

 0

 0

 0

 0

 0

 0

 0

 0

 0

 0

 0

 0

 0

 0

 0

 0

 0

 0

 0

 0

 0

 0

 0

 0

 0

 0

 0

 0

 0

 0

 0

 .05942406028527

 0

 0

 0

 0

 1.794204718763E-04

 0

 0

 0

 0

 7.17681887503E-05

 0

 0

 0

 0

 0

 0

 7.17681887503E-05

 7.17681887503E-05

 0

 0

 0

 0

 0

 0

 0

 0

 0

 0

 0

 .000107652283126

 0

 0

 0

 0

 .000107652283126

 .000107652283126

 0

 0

 5.91549295774557E-02

 0

 0

 0

 0

 0

 0

 0

 1.79420471876E-05

 1.79420471876E-05

 1.79420471876E-05

 5.38261415628E-05

 5.38261415628E-05

 5.38261415628E-05

 3.92930833407924E-02

 3.050148021894E-04

 3.58840943752E-05

 0

 .000107652283126

 3.58840943752E-05

 .000125594330313

 0

 0

 0

 0

 .00261953888939

 .00261953888939

 .008737776980348

 0

 .00662061541222

 .0010047546425

 .00102269668969

 3.58840943752E-05

 0

 5.38261415628E-05

 .000627971651565

 .000627971651565

 .0270027810173

 .0270027810173

 0

 0

 5.023773212524E-04

 0

 0

 1.79420471876E-05

 1.79420471876E-05

 0

 0

 0

 .000269130707814

 0

 .000269130707814

 0

 5.38261415628E-05

 5.38261415628E-05

 0

 0

 .000161478424688

 .000161478424688

 0

 0

 0

 0

 0

 0

 0

 1.79420471876E-05

 1.79420471876E-05

 1.79420471876E-05

 0

 0

 0

 0

 9.8142998116143E-03

 0

 0

 0

 0

 0

 0

 .000717681887503

 .000717681887503

 7.8586166681683E-03

 0

 5.38261415628E-05

 0

 3.58840943752E-05

 .00656678927066

 .00113034897282

 0

 7.17681887503E-05

 0

 0

 0

 0

 5.023773212526E-04

 1.79420471876E-05

 0

 0

 0

 0

 .000484435274065

 0

 3.229568493766E-04

 0

 0

 0

 0

 0

 3.58840943752E-05

 0

 0

 0

 .000215304566251

 0

 3.58840943752E-05

 3.58840943752E-05

 0

 0

 0

 0

 0

 0

 0

 0

 0

 0

 4.126670853138E-04

 0

 5.38261415628E-05

 .000197362519063

 .000161478424688

 0

 0

 0

 0

 0

 0

 0

 0

 0

 0

 0

 0

 0

 0

 0

 0

 .003373104871265

 .001937741096259

 .000699739840316

 0

 0

 .00111240692563

 .000125594330313

 .001435363775006

 .000466493226877

 0

 .000412667085314

 .000556203462815

 0

 1.5430160581336E-03

 1.5430160581336E-03

 0

 .000179420471876

 1.79420471876E-05

 0

 0

 .00134565353907

 0

 .00394725038127

 .00394725038127

 0

 0

 .00394725038127

 .00059208755719

 .00059208755719

 0

 .000125594330313

 .000466493226877

 0

 0

 0

 0

 0

 0

 0

 0

 0

 0

 .000089710235938

 0

 0

 0

 0

 0

 0

 0

 0

 .000089710235938

 0

 0

 3.58840943752E-05

 3.58840943752E-05

 0

 5.38261415628E-05

 0

 1.79420471876E-05

 3.58840943752E-05

 0

 0

 0

 0

 0

 0

 0

 0

 0

 0

 0

 0

 0

 0

 0

 0

 0

 0

 0

 3.1039741634559E-03

 3.1039741634559E-03

 .000753565981879

 .000753565981879

 .000753565981879

 0

 0

 0

 0

 0

 0

 0

 0

 1.9197990490739E-03

 0

 0

 1.9197990490739E-03

 0

 .000179420471876

 0

 0

 0

 0

 0

 7.17681887503E-05

 .00165066834126

 0

 0

 0

 0

 1.79420471876E-05

 0

 0

 0

 0

 0

 0

 0

 0

 0

 0

 0

 0

 0

 .000322956849377

 0

 0

 0

 0

 0

 0

 0

 .000322956849377

 0

 .000322956849377

 0

 0

 .000107652283126

 0

 0

 0

 0

 .000107652283126

 0

 .000107652283126

 0

 0

 0

 0

 0

 0

 0

 0

 0

 0

 0

 0

 0

 0

 0

 0

 0

 5.38261415628E-05

 0

 0

 0

 0

 0

 0

 0

 0

 0

 0

 0

 0

 0

 0

 0

 0

 0

 0

 5.38261415628E-05

 5.38261415628E-05

 0

 0

 5.38261415628E-05

 5.38261415628E-05

 0

 0

 0

 0

 0

 0

 0

 0

 0

 0

 0

 0

 0

 0

 0

 0

 0

 0

 0

 0

 0

 0

 0

 0

 0

 0

 0

 0

 0

 0

 0

 0

 0

 0

 0

 0

 0

 0

 0

 0

 0

 0

 0

 0

 0

 0

 0

 0

 0

 0

 0

 0

 0

 0

 0

 0

 0

 0

 0

 0

 0

 0

 0

 0

 0

 0

 0

 0

 0

 0

 0

 0

 0

 0

 0

 0

 0

 0

 0

 0

 0

 0

 0

 0

 2.1889297568872E-03

 2.0992195209492E-03

 1.435363775007E-04

 0

 0

 0

 0

 3.58840943752E-05

 0

 3.58840943752E-05

 0

 0

 0

 7.17681887503E-05

 7.17681887503E-05

 0

 0

 0

 0

 0

 0

 0

 0

 0

 0

 0

 0

 0

 0

 0

 0

 0

 0

 0

 0

 3.58840943752E-05

 0

 0

 3.58840943752E-05

 0

 0

 1.9556831434485E-03

 0

 0

 0

 0

 0

 0

 0

 0

 1.076522831255E-04

 1.79420471876E-05

 8.97102359379E-05

 0

 0

 0

 0

 .001848030860323

 .00172243653001

 .000125594330313

 .000089710235938

 .000089710235938

 0

 0

 0

 0

 0

 0

 0

 0

 3.58840943752E-05

 0

 0

 0

 0

 3.58840943752E-05

 0

 0

 0

 5.38261415628E-05

 0

 5.38261415628E-05

 0

 0

 0

 0

 0

 0

 0

 0

 0

 0

 0

 0

 0

 0

 0

 0

 0

 0

 0

 0

 0

 0

 0

 0

 0

 0

 0

 0

 0

 0

 0

 0

 0

 0

 0

 1.79420471876E-05

 1.79420471876E-05

 1.79420471876E-05

 1.79420471876E-05

 0

 1.79420471876E-05

 0

 0

 0

 0

 0

 0

 0

 0

 0

 0

 0

 0

 0

 0

 .000466493226877

 0

 0

 0

 0

 0

 0

 0

 0

 0

 0

 0

 0

 .000466493226877

 .000466493226877

 0

 0

 0

 0

 0

 0

 0

 0

 0

 0

 0

 .000125594330313

 .000125594330313

 0

 0

 0

 0

 .000340898896564

 .000340898896564

 7.356239346912E-04

 3.58840943752E-05

 0

 0

 0

 0

 3.58840943752E-05

 0

 0

 0

 0

 3.58840943752E-05

 0

 3.58840943752E-05

 0

 0

 0

 0

 0

 .000699739840316

 0

 0

 0

 0

 .000699739840316

 .000699739840316

 0

 .000305014802189

 0

 .000251188660626

 .000143536377501

 0

 0

 0

 0

 0

 0

 0

 0

 0

 0

 0

 .933165874226261

 .538494662240914

 1.076522831255E-04

 1.076522831255E-04

 0

 0

 1.79420471876E-05

 1.79420471876E-05

 0

 0

 0

 0

 0

 0

 0

 0

 0

 0

 0

 0

 0

 7.17681887503E-05

 0

 0

 0

 0

 0

 0

 0

 1.79420471876E-05

 1.79420471876E-05

 0

 0

 0

 0

 0

 1.79420471876E-05

 0

 0

 0

 0

 0

 0

 0

 0

 0

 0

 0

 0

 0

 0

 0

 0

 0

 0

 0

 5.38261415628E-05

 5.38261415628E-05

 0

 5.38261415628E-05

 0

 0

 0

 0

 0

 0

 0

 0

 0

 0

 0

 0

 6.3873687987768E-03

 0

 0

 0

 0

 3.408988965638E-04

 0

 0

 0

 .000125594330313

 5.38261415628E-05

 0

 0

 0

 0

 0

 .000161478424688

 0

 0

 0

 0

 0

 0

 0

 7.17681887503E-05

 7.17681887503E-05

 0

 0

 0

 0

 0

 .00044855117969

 .00044855117969

 0

 0

 .00199156723782

 .00199156723782

 .000843276217816

 3.58840943752E-05

 .000627971651565

 0

 0

 0

 8.97102359379E-05

 0

 1.79420471876E-05

 0

 0

 0

 0

 7.17681887503E-05

 0

 0

 0

 0

 0

 0

 0

 2.6913070781367E-03

 .000197362519063

 .00222481385126

 0

 3.58840943752E-05

 0

 0

 3.58840943752E-05

 0

 .000125594330313

 7.17681887503E-05

 0

 0

 0

 0

 1.435363775006E-04

 1.435363775006E-04

 0

 0

 0

 7.17681887503E-05

 7.17681887503E-05

 0

 0

 0

 0

 0

 0

 0

 0

 0

 0

 0

 0

 0

 .528859782901188

 0

 0

 .477096976765

 .477096976765

 5.17628061361876E-02

 1.79420471876E-05

 .051744864089

 0

 2.9245536915728E-03

 2.9245536915728E-03

 0

 0

 0

 0

 0

 0

 1.79420471876E-05

 0

 0

 3.58840943752E-05

 .00287072755001

 0

 0

 0

 0

 0

 0

 0

 0

 0

 0

 0

 0

 0

 1.79420471876E-05

 0

 0

 0

 0

 0

 0

 0

 0

 0

 0

 0

 0

 0

 1.79420471876E-05

 0

 0

 0

 0

 0

 0

 0

 0

 0

 0

 0

 0

 0

 0

 0

 0

 0

 0

 0

 0

 0

 1.79420471876E-05

 1.79420471876E-05

 0

 0

 0

 0

 0

 0

 0

 0

 0

 0

 0

 .394653269938159

 0

 0

 0

 .0109267067372

 .0109267067372

 .0109267067372

 0

 0

 0

 0

 0

 8.5762985556625E-03

 0

 0

 8.5583565084749E-03

 8.97102359379E-05

 0

 0

 .00814568942316

 .000107652283126

 0

 .000215304566251

 0

 0

 0

 0

 0

 0

 0

 0

 0

 0

 0

 0

 0

 0

 0

 0

 0

 0

 0

 0

 0

 0

 0

 0

 0

 0

 0

 0

 0

 0

 0

 0

 0

 0

 0

 0

 0

 0

 0

 0

 0

 0

 0

 0

 0

 0

 1.79420471876E-05

 0

 0

 0

 1.79420471876E-05

 0

 0

 0

 0

 0

 0

 0

 0

 0

 0

 1.79420471876E-05

 1.79420471876E-05

 0

 1.79420471876E-05

 0

 .360922221225494

 .360922221225494

 .0425226518346

 .000556203462815

 0

 3.58840943752E-05

 .0211177895398

 .00156095810532

 7.17681887503E-05

 3.58840943752E-05

 .00269130707814

 7.17681887503E-05

 .0467210908765

 0

 7.17681887503E-05

 .000430609132502

 7.17681887503E-05

 .00159684219969

 1.79420471876E-05

 0

 .243330043958

 1.79420471876E-05

 0

 0

 0

 0

 0

 0

 0

 0

 0

 0

 1.79420471876E-05

 1.79420471876E-05

 1.79420471876E-05

 0

 0

 0

 0

 0

 0

 0

 0

 1.79420471876E-05

 1.79420471876E-05

 1.79420471876E-05

 1.255943303136E-04

 1.79420471876E-05

 1.79420471876E-05

 0

 0

 0

 0

 0

 .000107652283126

 .000107652283126

 0

 0

 0

 0

 0

 0

 0

 0

 0

 0

 0

 0

 0

 3.58840943752E-05

 3.58840943752E-05

 3.58840943752E-05

 0

 0

 0

 .014012738853551

 .003139858257825

 3.58840943752E-05

 .000143536377501

 0

 3.58840943752E-05

 .00206333542657

 1.79420471876E-05

 .000843276217816

 .010872880595726

 0

 0

 .0107652283126

 0

 0

 .000107652283126

 0

 0

 0

 0

 0

 0

 0

 0

 0

 0

 0

 0

 0

 0

 0

 0

 0

 5.38261415628E-05

 5.38261415628E-05

 0

 0

 0

 0

 0

 0

 0

 0

 0

 0

 0

 3.58840943752E-05

 3.58840943752E-05

 0

 0

 3.58840943752E-05

 0

 0

 0

 0

 0

 0

 0

 0

 0

 0

 0

 1.79420471876E-05

 0

 0

 0

 0

 1.79420471876E-05

 1.79420471876E-05

 0

 0

 0

 0

 0

 0

 0

 0

 0

 0

 0

 0

 0

 0
