# Supplementary material for: First Insight into Microbiome Profiles of Myrmecophilous Beetles and Their Host, Red Wood Ant Formica polyctena (Hymenoptera: Formicidae)—A Case Study
Source: Insects. 2020 Feb 19;11(2):134. doi: 10.3390/insects11020134 (PMC7073670; doi:10.3390/insects11020134)
Supplement: Supplementary file 1 [file insects-11-00134-s001.zip › Supplementary Files/S2_Figure.zip!/Supplemental_Figure_S2/Supplemental_Figure_S2_MA3.html]

Javascript must be enabled to view this page.

magnitude

 .999999999999369

 .999999999999369

 0

 0

 0

 0

 0

 0

 .000207047131456

 .000207047131456

 .00018822466496

 .00018822466496

 .000056467399488

 0

 .000037644932992

 0

 .00009411233248

 0

 0

 0

 0

 0

 0

 0

 0

 0

 0

 .000018822466496

 .000018822466496

 .000018822466496

 0

 0

 0

 0

 0

 0

 0

 0

 0

 0

 0

 0

 0

 0

 0

 0

 0

 0

 0

 0

 0

 0

 0

 0

 0

 0

 0

 0

 0

 0

 0

 0

 0

 0

 0

 0

 0

 0

 0

 0

 0

 0

 0

 0

 0

 0

 0

 0

 0

 0

 0

 0

 0

 0

 0

 0

 0

 0

 0

 0

 0

 0

 0

 .092832404758344

 0

 0

 0

 0

 .000037644932992

 0

 0

 0

 0

 .000018822466496

 0

 0

 0

 0

 .000018822466496

 .000018822466496

 0

 0

 0

 0

 0

 0

 0

 0

 0

 0

 0

 0

 0

 .000018822466496

 0

 0

 0

 0

 .000018822466496

 .000018822466496

 0

 0

 .092512422827912

 0

 0

 0

 .000112934798976

 .000112934798976

 .000112934798976

 0

 0

 0

 0

 .000056467399488

 .000056467399488

 .000056467399488

 .061587110374938

 .001788134317122

 0

 0

 .00137404005421

 .000244692064448

 .000169402198464

 0

 0

 .000037644932992

 .000037644932992

 .0019010691161

 .0019010691161

 .007736033729864

 0

 .00434798976058

 .00208929378106

 .00103523565728

 .000056467399488

 .000131757265472

 .000075289865984

 .00018822466496

 .00018822466496

 .0499360036139

 .0499360036139

 0

 0

 .0004705616624

 0

 0

 0

 0

 0

 0

 0

 .000056467399488

 0

 .000056467399488

 0

 .00028233699744

 .00028233699744

 0

 0

 .000131757265472

 .000131757265472

 0

 0

 0

 0

 0

 0

 0

 0

 0

 0

 0

 0

 0

 0

 .021457611805488

 0

 0

 0

 0

 0

 0

 .000527029061888

 .000527029061888

 .019198915825964

 0

 0

 0

 0

 .0190295136275

 .000169402198464

 0

 0

 0

 0

 0

 0

 .000056467399488

 .000018822466496

 0

 0

 0

 0

 .000037644932992

 0

 .000432916729408

 0

 0

 0

 .000037644932992

 0

 0

 0

 0

 .000018822466496

 .000225869597952

 0

 0

 .000112934798976

 0

 0

 0

 0

 0

 0

 0

 0

 0

 .000037644932992

 .00124228278874

 0

 .00105405812378

 0

 .00018822466496

 0

 0

 0

 0

 0

 0

 0

 0

 .000112934798976

 .000112934798976

 0

 .000112934798976

 0

 0

 0

 0

 .002428098177984

 .000489384128896

 .000263514530944

 .000056467399488

 0

 .000075289865984

 .00009411233248

 .001938714049088

 .000734076193344

 0

 .000263514530944

 .0009411233248

 0

 .001750489384126

 .001750489384126

 0

 .000357626863424

 .000037644932992

 0

 0

 .00135521758771

 0

 .00385860563168

 .00385860563168

 0

 0

 .00385860563168

 .000677608793856

 .000677608793856

 0

 .00009411233248

 .000583496461376

 0

 0

 0

 0

 0

 0

 0

 0

 0

 0

 .00028233699744

 0

 0

 0

 0

 0

 0

 0

 0

 .00028233699744

 0

 0

 .00009411233248

 .00009411233248

 0

 .00018822466496

 0

 .000056467399488

 .00009411233248

 .000037644932992

 0

 0

 0

 0

 0

 0

 0

 0

 0

 0

 0

 0

 0

 0

 0

 0

 0

 0

 .001656377051648

 .001656377051648

 .000037644932992

 0

 0

 0

 0

 .000037644932992

 0

 .000037644932992

 0

 0

 0

 .001524619786176

 0

 0

 .001524619786176

 0

 .000959945791296

 0

 0

 0

 .000018822466496

 0

 0

 .000545851528384

 0

 0

 0

 0

 0

 0

 0

 0

 0

 0

 0

 0

 0

 0

 0

 0

 0

 0

 .00009411233248

 0

 0

 0

 0

 0

 0

 0

 .00009411233248

 0

 .00009411233248

 0

 0

 0

 0

 0

 0

 0

 0

 0

 0

 0

 0

 0

 0

 0

 0

 0

 0

 0

 0

 0

 0

 0

 0

 0

 0

 0

 0

 0

 0

 0

 0

 0

 0

 0

 0

 0

 0

 0

 0

 0

 0

 0

 0

 0

 0

 0

 0

 0

 0

 0

 0

 0

 0

 0

 0

 0

 0

 0

 0

 0

 0

 0

 0

 0

 0

 0

 0

 0

 0

 0

 0

 0

 0

 0

 0

 0

 0

 0

 0

 0

 0

 0

 0

 0

 0

 0

 0

 0

 0

 0

 0

 0

 0

 0

 0

 0

 0

 0

 0

 0

 0

 0

 0

 0

 0

 0

 0

 0

 0

 0

 0

 0

 0

 0

 0

 0

 0

 0

 0

 0

 0

 0

 0

 0

 0

 0

 0

 0

 0

 0

 0

 0

 0

 0

 0

 .004028007830146

 .00400918536365

 .001072880590272

 0

 0

 0

 0

 0

 0

 0

 0

 0

 0

 0

 0

 0

 0

 0

 0

 0

 0

 0

 0

 0

 0

 0

 0

 0

 0

 0

 0

 0

 0

 0

 0

 .001072880590272

 0

 .00018822466496

 .000884655925312

 0

 0

 .002936304773378

 0

 0

 0

 0

 0

 0

 0

 0

 .000978768257792

 .000075289865984

 .000903478391808

 .000037644932992

 .000037644932992

 0

 0

 .001919891582594

 .00184460171661

 .000075289865984

 .000018822466496

 .000018822466496

 0

 0

 0

 0

 0

 0

 0

 0

 .000018822466496

 .000018822466496

 0

 0

 0

 0

 0

 0

 0

 0

 0

 0

 0

 0

 0

 0

 0

 0

 0

 0

 0

 0

 0

 0

 0

 0

 0

 0

 0

 0

 0

 0

 0

 0

 0

 0

 0

 0

 0

 0

 0

 0

 0

 0

 0

 0

 0

 .000018822466496

 .000018822466496

 .000018822466496

 .000018822466496

 .000018822466496

 0

 0

 0

 0

 0

 0

 0

 0

 0

 0

 0

 0

 0

 0

 0

 .000207047131456

 .000018822466496

 .000018822466496

 .000018822466496

 .000018822466496

 0

 0

 0

 0

 0

 0

 0

 0

 .00018822466496

 .00018822466496

 .000056467399488

 .000056467399488

 0

 0

 0

 0

 0

 0

 0

 0

 0

 .000112934798976

 .000112934798976

 0

 0

 0

 0

 .000018822466496

 .000018822466496

 .000207047131456

 0

 0

 0

 0

 0

 0

 0

 0

 0

 0

 0

 0

 0

 0

 0

 0

 0

 0

 .000207047131456

 0

 0

 0

 0

 .000207047131456

 .000207047131456

 .000018822466496

 .000037644932992

 0

 .000018822466496

 .00009411233248

 .000037644932992

 0

 0

 0

 0

 0

 0

 0

 0

 0

 0

 .900843246498367

 .679641620237649

 .000075289865984

 .000075289865984

 0

 0

 0

 .000018822466496

 0

 0

 0

 0

 0

 0

 0

 0

 0

 0

 0

 0

 0

 .000056467399488

 0

 0

 0

 0

 0

 0

 0

 0

 0

 0

 0

 0

 0

 0

 0

 0

 0

 0

 0

 0

 0

 0

 0

 0

 0

 0

 0

 0

 0

 0

 0

 0

 0

 0

 0

 0

 0

 0

 0

 0

 0

 0

 0

 0

 0

 0

 0

 0

 0

 0

 .004686794157511

 0

 0

 0

 0

 .000150579731968

 0

 0

 0

 .000056467399488

 .00009411233248

 0

 0

 0

 0

 0

 0

 0

 0

 0

 0

 0

 0

 0

 0

 0

 0

 0

 0

 0

 0

 .000056467399488

 .000056467399488

 0

 0

 .00271043517543

 .00271043517543

 .000508206595392

 .000056467399488

 .00018822466496

 0

 0

 0

 .00018822466496

 0

 0

 0

 0

 0

 0

 .000018822466496

 .000056467399488

 0

 0

 0

 0

 0

 0

 .001261105255233

 .000131757265472

 .000978768257793

 0

 0

 0

 0

 .000018822466496

 0

 .000131757265472

 0

 0

 0

 0

 0

 .000075289865984

 .000075289865984

 0

 0

 0

 .000018822466496

 .000056467399488

 0

 0

 0

 0

 0

 0

 0

 0

 0

 0

 0

 0

 0

 .6694586658633

 0

 0

 .647248155398

 .647248155398

 .0222105104653

 0

 .0222105104653

 0

 .00534558048487

 .00534558048487

 0

 0

 0

 0

 0

 0

 0

 0

 0

 0

 .00534558048487

 0

 0

 0

 0

 0

 0

 0

 0

 0

 0

 0

 0

 0

 0

 0

 0

 0

 0

 0

 0

 0

 0

 0

 0

 0

 0

 0

 0

 0

 0

 0

 0

 0

 0

 0

 0

 0

 0

 0

 0

 0

 0

 0

 0

 0

 0

 0

 0

 0

 0

 0

 0

 0

 0

 0

 0

 0

 0

 0

 0

 0

 0

 .221201626260718

 0

 0

 0

 .0028233699744

 .0028233699744

 .0028233699744

 0

 0

 0

 0

 0

 .003613913567238

 0

 0

 .00355744616775

 .000018822466496

 0

 0

 .00336922150279

 0

 0

 .000150579731968

 0

 0

 0

 0

 0

 0

 0

 0

 0

 0

 .000018822466496

 0

 0

 0

 0

 0

 0

 0

 0

 0

 0

 0

 0

 0

 0

 0

 0

 0

 0

 0

 0

 0

 0

 0

 .000056467399488

 0

 .000056467399488

 0

 0

 0

 0

 0

 0

 0

 0

 0

 0

 0

 0

 0

 0

 0

 0

 0

 0

 0

 0

 0

 0

 0

 0

 .000131757265472

 .000131757265472

 0

 .000131757265472

 0

 .210585755156972

 .210585755156972

 .00303041710586

 .000131757265472

 0

 0

 .00131757265472

 .000018822466496

 .000037644932992

 0

 .000263514530944

 .000018822466496

 0

 0

 0

 0

 0

 .000037644932992

 0

 0

 .205729558801

 0

 0

 0

 0

 0

 0

 0

 0

 0

 0

 0

 0

 0

 0

 0

 0

 0

 0

 0

 .000018822466496

 .000018822466496

 .000018822466496

 0

 0

 0

 .00009411233248

 .00009411233248

 .000056467399488

 0

 0

 0

 .000037644932992

 0

 0

 0

 0

 0

 0

 0

 0

 0

 0

 0

 0

 0

 0

 0

 0

 0

 0

 0

 0

 0

 0

 .00393389549766

 .00254103297696

 .000056467399488

 .000018822466496

 .000018822466496

 0

 .00101641319078

 0

 .0014305074537

 .0013928625207

 0

 0

 .0013928625207

 0

 0

 0

 0

 0

 0

 0

 0

 0

 0

 0

 0

 0

 0

 0

 0

 0

 0

 0

 0

 0

 0

 0

 0

 0

 0

 0

 0

 0

 0

 0

 0

 0

 0

 0

 0

 0

 0

 0

 0

 0

 0

 0

 0

 0

 0

 0

 0

 0

 0

 0

 0

 0

 0

 0

 0

 0

 0

 0

 0

 0

 0

 0

 0

 0

 0

 0

 0

 0

 0
