# Supplementary material for: First Insight into Microbiome Profiles of Myrmecophilous Beetles and Their Host, Red Wood Ant Formica polyctena (Hymenoptera: Formicidae)—A Case Study
Source: Insects. 2020 Feb 19;11(2):134. doi: 10.3390/insects11020134 (PMC7073670; doi:10.3390/insects11020134)
Supplement: Supplementary file 1 [file insects-11-00134-s001.zip › Supplementary Files/S2_Figure.zip!/Supplemental_Figure_S2/Supplemental_Figure_S2_MS1.html]

Javascript must be enabled to view this page.

magnitude

 1.00000000000034

 1.00000000000034

 9.78266186229E-05

 9.78266186229E-05

 9.78266186229E-05

 9.78266186229E-05

 0

 9.78266186229E-05

 2.282621101201E-04

 1.304354914972E-04

 1.304354914972E-04

 6.52177457486E-05

 0

 0

 0

 0

 0

 0

 0

 0

 6.52177457486E-05

 6.52177457486E-05

 6.52177457486E-05

 0

 0

 0

 0

 0

 0

 0

 0

 0

 0

 0

 0

 0

 0

 0

 0

 0

 0

 0

 0

 0

 0

 0

 0

 0

 0

 0

 0

 0

 0

 0

 0

 0

 0

 9.78266186229E-05

 0

 0

 0

 0

 9.78266186229E-05

 9.78266186229E-05

 9.78266186229E-05

 0

 0

 0

 0

 0

 0

 0

 0

 0

 0

 0

 0

 0

 0

 0

 0

 0

 0

 0

 0

 0

 0

 0

 0

 0

 0

 0

 0

 3.66034598013696E-02

 0

 0

 0

 0

 0

 0

 0

 0

 0

 0

 0

 0

 0

 0

 0

 0

 0

 0

 0

 0

 0

 0

 0

 0

 0

 0

 0

 0

 0

 0

 0

 0

 0

 0

 0

 0

 0

 0

 3.59349779074458E-02

 0

 0

 0

 0

 0

 0

 0

 0

 0

 0

 .000277175419432

 .000277175419432

 .000277175419432

 1.01250550274752E-02

 .007010907667976

 0

 0

 .00344023608824

 .00280436306719

 .000766308512546

 0

 0

 0

 0

 .00153261702509

 .00153261702509

 1.5815303344092E-03

 0

 0

 1.63044364372E-05

 .0014021815336

 0

 0

 .000163044364372

 0

 0

 0

 0

 0

 0

 1.630443643715E-04

 0

 0

 6.52177457486E-05

 6.52177457486E-05

 0

 0

 0

 0

 0

 0

 0

 0

 0

 0

 0

 0

 0

 9.78266186229E-05

 0

 9.78266186229E-05

 0

 0

 0

 0

 1.630443643712E-04

 1.630443643712E-04

 0

 .000146739927934

 0

 1.63044364372E-05

 0

 3.2934961603031E-03

 8.15221821858E-05

 8.15221821858E-05

 0

 0

 0

 0

 0

 0

 0

 0

 0

 0

 0

 0

 0

 0

 0

 0

 .000472828656677

 .000472828656677

 0

 0

 0

 0

 0

 0

 0

 0

 0

 0

 0

 0

 0

 0

 0

 0

 0

 0

 0

 0

 0

 0

 0

 0

 0

 0

 0

 0

 0

 0

 0

 0

 0

 2.7391453214403E-03

 .000130435491497

 .000309784292306

 3.26088728743E-05

 .00159783477084

 0

 0

 .000668481893923

 0

 0

 0

 0

 0

 0

 0

 0

 0

 0

 0

 0

 0

 .015945738835497

 .002543492084197

 .00143479040647

 0

 0

 .000163044364372

 .000945657313355

 .0134022467513

 .0134022467513

 0

 0

 0

 0

 8.15221821858E-05

 8.15221821858E-05

 0

 1.63044364372E-05

 0

 0

 0

 0

 6.52177457486E-05

 .00577177049875

 .00577177049875

 0

 0

 .00577177049875

 .00011413105506

 .00011413105506

 0

 0

 .00011413105506

 0

 0

 0

 0

 0

 0

 0

 0

 0

 0

 6.684818939238E-04

 0

 0

 0

 0

 0

 0

 0

 0

 6.684818939238E-04

 0

 0

 8.15221821858E-05

 8.15221821858E-05

 0

 .000586959711738

 0

 .000586959711738

 0

 0

 0

 0

 0

 0

 0

 0

 0

 0

 0

 0

 0

 0

 0

 0

 0

 0

 0

 0

 1.9565323724582E-03

 1.9565323724582E-03

 .000260870982994

 0

 0

 0

 0

 .000260870982994

 0

 .000260870982994

 0

 0

 0

 .000603264148175

 0

 0

 .000603264148175

 0

 0

 .000603264148175

 0

 0

 0

 0

 0

 0

 0

 0

 0

 0

 0

 0

 0

 .000195653237246

 0

 0

 .000195653237246

 .000195653237246

 0

 0

 0

 0

 0

 0

 1.63044364372E-05

 0

 0

 0

 0

 0

 0

 0

 1.63044364372E-05

 0

 1.63044364372E-05

 0

 0

 .000880439567606

 0

 0

 0

 0

 .000880439567606

 0

 0

 .000766308512546

 .00011413105506

 0

 0

 0

 0

 0

 0

 0

 0

 0

 0

 0

 0

 0

 0

 0

 .000260870982994

 0

 0

 0

 0

 0

 0

 0

 0

 0

 0

 0

 0

 0

 0

 0

 0

 0

 0

 .000260870982994

 .000260870982994

 .000260870982994

 .000260870982994

 0

 0

 0

 0

 0

 0

 0

 0

 0

 0

 0

 0

 0

 0

 0

 0

 0

 0

 0

 0

 0

 0

 0

 0

 0

 0

 0

 0

 0

 0

 0

 0

 0

 0

 0

 0

 0

 0

 0

 0

 0

 0

 0

 0

 0

 0

 0

 0

 0

 0

 0

 0

 0

 0

 0

 0

 0

 0

 0

 0

 0

 0

 0

 0

 0

 0

 0

 0

 0

 0

 0

 0

 0

 0

 0

 0

 0

 0

 0

 0

 0

 0

 0

 0

 0

 0

 4.1250224186002E-03

 3.5380627068622E-03

 .000847830694733

 0

 0

 0

 0

 0

 0

 0

 0

 0

 0

 0

 0

 0

 0

 4.89133093115E-05

 0

 4.89133093115E-05

 .000163044364372

 0

 0

 .000163044364372

 0

 0

 0

 0

 4.89133093115E-05

 0

 0

 4.89133093115E-05

 0

 0

 0

 .000586959711738

 0

 0

 .000586959711738

 0

 0

 2.6902320121292E-03

 0

 0

 0

 0

 0

 0

 0

 0

 1.956532372462E-04

 1.63044364372E-05

 .000179348800809

 .000326088728743

 .000326088728743

 0

 0

 .00216849004614

 .00115761498704

 .0010108750591

 .000586959711738

 .000586959711738

 .000586959711738

 0

 .000586959711738

 0

 0

 0

 0

 0

 0

 0

 0

 0

 0

 0

 0

 0

 0

 0

 0

 0

 0

 0

 0

 0

 0

 0

 0

 0

 0

 0

 0

 0

 0

 0

 0

 0

 0

 0

 0

 0

 0

 0

 0

 0

 0

 0

 0

 0

 0

 0

 0

 0

 0

 0

 0

 0

 0

 0

 0

 0

 0

 0

 0

 0

 0

 0

 0

 0

 0

 0

 0

 0

 0

 0

 0

 .000945657313355

 0

 0

 0

 0

 0

 0

 0

 0

 0

 0

 0

 0

 .000945657313355

 .000945657313355

 0

 0

 0

 0

 0

 0

 0

 0

 0

 0

 0

 .000945657313355

 .000945657313355

 0

 0

 0

 0

 0

 0

 .000244566546557

 .00011413105506

 0

 0

 0

 0

 .00011413105506

 0

 0

 0

 0

 .00011413105506

 0

 .00011413105506

 0

 0

 0

 0

 0

 .000130435491497

 0

 0

 0

 0

 0

 0

 0

 0

 0

 0

 0

 0

 0

 0

 0

 0

 .000130435491497

 0

 0

 .000130435491497

 .000130435491497

 0

 .95530953972614

 .947972543329422

 0

 0

 0

 0

 0

 0

 0

 0

 0

 0

 0

 0

 0

 0

 0

 0

 0

 0

 0

 0

 0

 0

 0

 0

 0

 0

 0

 .000391306474492

 .000391306474492

 0

 0

 0

 0

 .000391306474492

 0

 0

 0

 0

 0

 0

 0

 0

 0

 0

 0

 0

 0

 0

 0

 0

 0

 0

 0

 0

 0

 0

 0

 0

 0

 0

 0

 0

 0

 6.52177457486E-05

 6.52177457486E-05

 6.52177457486E-05

 3.26088728743E-05

 3.26088728743E-05

 3.26088728743E-05

 0

 2.3967521562612E-03

 0

 0

 0

 0

 .000619568584612

 0

 0

 0

 0

 .000619568584612

 0

 0

 0

 0

 0

 0

 0

 0

 0

 0

 0

 0

 0

 0

 0

 0

 0

 0

 0

 0

 0

 0

 0

 0

 0

 0

 6.847863303602E-04

 .000668481893923

 1.63044364372E-05

 0

 0

 0

 0

 0

 0

 0

 0

 0

 0

 0

 0

 0

 0

 0

 0

 0

 0

 .001092397241289

 .000244566546557

 .000277175419432

 0

 .0005706552753

 0

 0

 0

 0

 0

 0

 0

 0

 0

 0

 .001027179495541

 .001027179495541

 0

 0

 0

 .000505437529552

 .000521741965989

 0

 0

 0

 0

 0

 1.63044364372E-05

 0

 0

 0

 1.63044364372E-05

 1.63044364372E-05

 0

 0

 .941760553046863

 0

 0

 .941206202208

 .941206202208

 .000554350838863

 0

 .000554350838863

 0

 2.2826211012042E-03

 2.2826211012042E-03

 1.63044364372E-05

 0

 0

 0

 0

 0

 0

 0

 0

 .000244566546557

 .00202175011821

 0

 0

 0

 0

 0

 0

 0

 0

 0

 0

 0

 0

 0

 .00011413105506

 0

 0

 0

 0

 0

 0

 0

 0

 0

 0

 0

 0

 0

 .00011413105506

 0

 0

 0

 0

 0

 0

 0

 0

 0

 0

 0

 0

 0

 0

 0

 0

 0

 0

 0

 0

 0

 0

 0

 .00011413105506

 .00011413105506

 0

 0

 0

 0

 0

 0

 0

 0

 0

 7.2228653416582E-03

 0

 0

 0

 0

 0

 0

 0

 0

 0

 0

 0

 3.6358893254825E-03

 0

 0

 2.2010989190135E-03

 .000260870982994

 .000146739927934

 8.15221821858E-05

 0

 1.63044364372E-05

 0

 .000130435491497

 0

 0

 .0011413105506

 0

 .00022826211012

 0

 .000146739927934

 0

 0

 0

 4.89133093115E-05

 0

 0

 0

 0

 0

 0

 0

 0

 0

 0

 0

 0

 0

 0

 0

 0

 0

 0

 0

 0

 0

 0

 0

 .000130435491497

 0

 .000130435491497

 .000668481893923

 0

 .000668481893923

 0

 0

 0

 0

 0

 0

 .000635873021049

 0

 0

 0

 .000635873021049

 0

 0

 0

 0

 0

 .000489133093115

 0

 0

 .000489133093115

 .000489133093115

 0

 0

 0

 0

 0

 8.15221821858E-05

 8.15221821858E-05

 0

 0

 0

 0

 3.26088728743E-05

 0

 0

 0

 0

 0

 0

 0

 0

 0

 3.26088728743E-05

 0

 0

 0

 1.63044364372E-05

 0

 0

 0

 0

 0

 0

 0

 0

 0

 0

 0

 0

 0

 0

 .000260870982994

 .000260870982994

 .000260870982994

 0

 0

 0

 0

 0

 4.89133093115E-05

 4.89133093115E-05

 4.89133093115E-05

 1.5326170250944E-03

 4.891330931144E-04

 .00034239316518

 0

 0

 0

 9.78266186229E-05

 4.89133093115E-05

 .00104348393198

 .00104348393198

 0

 0

 0

 0

 0

 0

 0

 0

 0

 0

 0

 0

 0

 0

 0

 0

 0

 0

 0

 .001173919423475

 .000423915347366

 0

 1.63044364372E-05

 0

 0

 8.15221821858E-05

 0

 .000326088728743

 .000750004076109

 0

 0

 .000130435491497

 0

 0

 .000619568584612

 0

 0

 0

 0

 0

 0

 0

 0

 0

 0

 0

 0

 4.89133093115E-05

 4.89133093115E-05

 4.89133093115E-05

 4.89133093115E-05

 4.89133093115E-05

 .000179348800809

 .000179348800809

 0

 0

 0

 0

 0

 0

 0

 0

 0

 0

 0

 .000179348800809

 .000179348800809

 0

 0

 .000179348800809

 0

 0

 0

 0

 0

 0

 0

 0

 0

 0

 0

 0

 0

 0

 0

 0

 0

 0

 0

 0

 0

 0

 0

 0

 0

 0

 0

 0

 0

 0

 0

 0
