# Supplementary material for: First Insight into Microbiome Profiles of Myrmecophilous Beetles and Their Host, Red Wood Ant Formica polyctena (Hymenoptera: Formicidae)—A Case Study
Source: Insects. 2020 Feb 19;11(2):134. doi: 10.3390/insects11020134 (PMC7073670; doi:10.3390/insects11020134)
Supplement: Supplementary file 1 [file insects-11-00134-s001.zip › Supplementary Files/S2_Figure.zip!/Supplemental_Figure_S2/Supplemental_Figure_S2_MS2.html]

Javascript must be enabled to view this page.

magnitude

 1.00000000000039

 1.00000000000039

 2.00819342919E-05

 2.00819342919E-05

 2.00819342919E-05

 2.00819342919E-05

 0

 2.00819342919E-05

 6.225399630488E-04

 6.024580287569E-04

 2.209012772109E-04

 2.209012772109E-04

 6.02458028757E-05

 0

 6.02458028757E-05

 0

 2.00819342919E-05

 0

 0

 0

 8.03277371676E-05

 0

 0

 0

 0

 0

 0

 .000381556751546

 .000381556751546

 .000381556751546

 0

 0

 0

 0

 0

 0

 0

 0

 0

 0

 0

 0

 0

 0

 0

 0

 0

 0

 0

 0

 0

 0

 0

 0

 0

 0

 0

 2.00819342919E-05

 0

 0

 0

 0

 2.00819342919E-05

 2.00819342919E-05

 2.00819342919E-05

 0

 0

 0

 0

 0

 0

 0

 0

 0

 0

 0

 0

 0

 0

 0

 0

 0

 0

 0

 0

 0

 0

 0

 0

 0

 0

 0

 0

 4.29552574504056E-02

 0

 0

 0

 0

 3.614748172539E-04

 0

 0

 0

 0

 0

 0

 0

 0

 0

 0

 0

 0

 0

 .000160655474335

 0

 0

 0

 0

 0

 0

 .000160655474335

 .000160655474335

 0

 0

 2.008193429189E-04

 0

 0

 .000180737408627

 .000180737408627

 2.00819342919E-05

 2.00819342919E-05

 0

 0

 4.20515704072698E-02

 0

 0

 0

 8.03277371676E-05

 8.03277371676E-05

 8.03277371676E-05

 0

 0

 0

 0

 .000481966423006

 .000481966423006

 .000481966423006

 8.8360510884395E-03

 .002891798538033

 0

 0

 .00052213029159

 .00160655474335

 .000763113503093

 0

 0

 0

 0

 .00295204434091

 .00295204434091

 2.9721262752046E-03

 0

 0

 .000281147080087

 .00261065145795

 8.03277371676E-05

 0

 0

 0

 0

 2.00819342919E-05

 2.00819342919E-05

 0

 0

 1.6065547433576E-03

 0

 0

 .000200819342919

 .000200819342919

 0

 0

 0

 .000220901277211

 0

 .000220901277211

 0

 8.03277371676E-05

 8.03277371676E-05

 0

 0

 .00110450638606

 .00110450638606

 0

 0

 0

 0

 0

 0

 0

 0

 0

 0

 0

 0

 0

 0

 7.4102337537096E-03

 4.01638685838E-05

 4.01638685838E-05

 0

 0

 0

 0

 0

 0

 8.03277371676E-05

 0

 0

 0

 0

 2.00819342919E-05

 2.00819342919E-05

 4.01638685838E-05

 0

 0

 2.00819342919E-05

 0

 2.00819342919E-05

 0

 0

 0

 0

 0

 0

 0

 0

 5.422122258815E-04

 4.01638685838E-05

 0

 0

 0

 0

 0

 0

 0

 0

 0

 0

 6.02458028757E-05

 .000441802554422

 0

 0

 0

 0

 0

 0

 0

 0

 0

 0

 6.7274479877848E-03

 0

 4.01638685838E-05

 .000120491605751

 .00656679251345

 0

 0

 0

 0

 0

 0

 0

 0

 0

 0

 0

 0

 0

 0

 0

 0

 1.22499799180676E-02

 8.03277371676E-05

 0

 4.01638685838E-05

 0

 0

 4.01638685838E-05

 .0121696521809

 .0121696521809

 0

 0

 0

 0

 .00144589926902

 .00144589926902

 0

 .00042172062013

 0

 0

 0

 .00102417864889

 0

 .0086954775484

 .0086954775484

 0

 0

 .0086954775484

 1.2450799261019E-03

 1.2450799261019E-03

 2.00819342919E-05

 0

 .00122499799181

 0

 0

 0

 0

 0

 0

 0

 0

 0

 0

 5.422122258819E-04

 .00052213029159

 0

 0

 .00052213029159

 .00052213029159

 0

 0

 0

 2.00819342919E-05

 0

 0

 2.00819342919E-05

 2.00819342919E-05

 0

 0

 0

 0

 0

 0

 0

 0

 0

 0

 0

 0

 0

 0

 0

 0

 0

 0

 0

 0

 0

 0

 0

 0

 8.836051088434E-04

 8.836051088434E-04

 4.418025544218E-04

 0

 0

 0

 0

 4.01638685838E-05

 0

 4.01638685838E-05

 0

 .000401638685838

 .000401638685838

 2.00819342919E-05

 0

 0

 2.00819342919E-05

 0

 2.00819342919E-05

 0

 0

 0

 0

 0

 0

 0

 0

 0

 0

 0

 0

 0

 0

 .000361474817254

 .000361474817254

 .000361474817254

 0

 0

 0

 0

 0

 0

 0

 0

 4.01638685838E-05

 0

 0

 2.00819342919E-05

 0

 2.00819342919E-05

 0

 0

 2.00819342919E-05

 0

 2.00819342919E-05

 0

 0

 2.00819342919E-05

 0

 0

 0

 0

 2.00819342919E-05

 0

 0

 2.00819342919E-05

 0

 0

 0

 0

 0

 0

 0

 0

 0

 0

 0

 0

 0

 0

 0

 0

 0

 0

 0

 0

 0

 0

 0

 0

 0

 0

 0

 0

 0

 0

 0

 0

 0

 0

 0

 0

 0

 0

 0

 0

 0

 0

 0

 0

 0

 0

 0

 0

 0

 0

 0

 0

 0

 0

 0

 0

 0

 0

 0

 0

 0

 0

 0

 0

 0

 0

 0

 0

 0

 0

 0

 0

 0

 0

 0

 0

 0

 0

 0

 0

 0

 0

 0

 0

 0

 0

 0

 0

 0

 0

 0

 0

 0

 0

 2.00819342919E-05

 2.00819342919E-05

 2.00819342919E-05

 2.00819342919E-05

 2.00819342919E-05

 0

 0

 0

 0

 0

 0

 0

 0

 0

 0

 0

 0

 0

 0

 0

 0

 0

 0

 0

 0

 0

 0

 0

 0

 0

 0

 6.3659731705289E-03

 6.0245802875669E-03

 1.6266366776419E-03

 2.00819342919E-05

 2.00819342919E-05

 0

 0

 0

 0

 0

 0

 0

 0

 0

 0

 0

 0

 0

 0

 0

 0

 0

 0

 0

 0

 0

 0

 0

 0

 0

 0

 0

 0

 0

 0

 .00160655474335

 0

 0

 .00160655474335

 0

 0

 .004397943609925

 .000783195437385

 .000783195437385

 0

 0

 0

 0

 0

 0

 .000120491605751

 0

 .000120491605751

 0

 0

 .000301229014379

 .000301229014379

 .00319302755241

 .00212868503494

 .00106434251747

 0

 0

 0

 0

 0

 0

 0

 0

 0

 0

 0

 0

 0

 0

 0

 0

 0

 0

 0

 0

 0

 0

 0

 0

 0

 0

 0

 0

 0

 0

 0

 0

 0

 0

 0

 0

 0

 0

 0

 0

 0

 0

 .000341392882962

 .000341392882962

 .000341392882962

 0

 0

 0

 0

 .000341392882962

 0

 0

 0

 0

 0

 0

 0

 0

 0

 0

 0

 0

 0

 0

 0

 0

 0

 0

 0

 0

 0

 0

 0

 0

 0

 0

 0

 .000622539963049

 0

 0

 0

 0

 0

 0

 0

 0

 0

 0

 0

 0

 .000622539963049

 .000622539963049

 0

 0

 0

 0

 0

 0

 0

 0

 0

 0

 0

 .000622539963049

 .000622539963049

 0

 0

 0

 0

 0

 0

 1.5061450718929E-03

 0

 0

 0

 0

 0

 0

 0

 0

 0

 0

 0

 0

 0

 0

 0

 0

 0

 0

 1.5061450718929E-03

 2.00819342919E-05

 2.00819342919E-05

 2.00819342919E-05

 0

 .001004096714595

 .001004096714595

 0

 .000381556751546

 0

 .000401638685838

 .000220901277211

 0

 .000481966423006

 .000481966423006

 .000481966423006

 0

 0

 0

 0

 0

 0

 0

 .944413205880386

 .937806249498344

 .000361474817254

 .000361474817254

 .000361474817254

 0

 0

 0

 0

 0

 0

 0

 0

 0

 0

 0

 0

 0

 0

 0

 0

 0

 0

 0

 0

 0

 0

 0

 0

 6.426218973406E-04

 4.418025544216E-04

 .000361474817254

 0

 0

 0

 0

 8.03277371676E-05

 .000200819342919

 0

 .000200819342919

 0

 0

 0

 0

 0

 0

 0

 0

 0

 0

 0

 0

 .000180737408627

 .000180737408627

 .000180737408627

 0

 .00094385091172

 .00094385091172

 0

 .00094385091172

 0

 0

 0

 0

 0

 2.00819342919E-05

 2.00819342919E-05

 2.00819342919E-05

 0

 0

 0

 0

 8.0930195196362E-03

 0

 0

 0

 0

 .000281147080087

 0

 0

 0

 0

 .000281147080087

 0

 0

 0

 0

 0

 0

 0

 0

 2.00819342919E-05

 2.00819342919E-05

 0

 0

 0

 0

 0

 0

 0

 0

 0

 0

 0

 0

 0

 0

 0

 0

 9.237689774281E-04

 2.00819342919E-05

 6.02458028757E-05

 .000240983211503

 0

 0

 8.03277371676E-05

 0

 2.00819342919E-05

 0

 0

 0

 0

 .000321310948671

 .000180737408627

 0

 0

 0

 0

 0

 0

 6.8680215278292E-03

 .000883605108844

 .00359466623825

 0

 6.02458028757E-05

 0

 0

 2.00819342919E-05

 0

 8.03277371676E-05

 .0022290947064

 0

 0

 0

 0

 .001144670254639

 .001144670254639

 0

 0

 0

 .000361474817254

 .000301229014379

 0

 .000481966423006

 0

 0

 0

 0

 0

 0

 0

 0

 0

 0

 0

 .9262591372805

 0

 0

 .913225961925

 .913225961925

 .0130331753555

 0

 .0130331753555

 0

 .000160655474335

 .000160655474335

 0

 0

 0

 0

 0

 0

 0

 0

 0

 0

 .000160655474335

 0

 0

 0

 0

 0

 0

 0

 0

 0

 0

 0

 0

 0

 0

 0

 0

 0

 0

 0

 0

 0

 0

 0

 0

 0

 0

 0

 0

 0

 0

 0

 0

 0

 0

 0

 0

 0

 0

 0

 0

 0

 0

 0

 0

 0

 0

 0

 0

 0

 0

 0

 0

 0

 0

 0

 0

 0

 0

 0

 0

 0

 0

 6.6069563820424E-03

 0

 0

 0

 0

 0

 0

 0

 0

 0

 0

 0

 3.4540926982142E-03

 0

 0

 2.9118804723324E-03

 .00010040967146

 0

 0

 0

 4.01638685838E-05

 0

 2.00819342919E-05

 0

 0

 .00247007791791

 0

 0

 0

 0

 0

 0

 0

 4.01638685838E-05

 0

 0

 .00010040967146

 2.00819342919E-05

 .000120491605751

 0

 0

 0

 0

 0

 0

 0

 0

 0

 0

 0

 0

 0

 0

 0

 .000502048357298

 0

 .000502048357298

 0

 0

 0

 0

 0

 0

 0

 0

 0

 0

 0

 0

 4.01638685838E-05

 0

 4.01638685838E-05

 0

 0

 0

 0

 0

 0

 0

 0

 0

 0

 0

 0

 0

 0

 0

 0

 0

 7.229496345086E-04

 7.229496345086E-04

 4.01638685838E-05

 0

 2.00819342919E-05

 0

 .000381556751546

 0

 .000261065145795

 0

 0

 0

 0

 0

 0

 0

 0

 2.00819342919E-05

 0

 0

 0

 0

 0

 0

 0

 0

 0

 0

 0

 0

 0

 0

 0

 0

 0

 0

 0

 0

 0

 0

 0

 0

 0

 8.03277371676E-05

 8.03277371676E-05

 8.03277371676E-05

 3.614748172545E-04

 2.811470800869E-04

 .000261065145795

 0

 2.00819342919E-05

 0

 0

 0

 8.03277371676E-05

 8.03277371676E-05

 0

 0

 0

 0

 0

 0

 0

 0

 0

 0

 .000140573540043

 .000140573540043

 .000140573540043

 0

 0

 0

 0

 0

 0

 1.8475379548545E-03

 1.7872921519788E-03

 .000220901277211

 .00116475218893

 .000361474817254

 0

 2.00819342919E-05

 0

 2.00819342919E-05

 6.02458028757E-05

 0

 0

 0

 0

 6.02458028757E-05

 0

 0

 0

 0

 0

 0

 0

 0

 0

 0

 0

 0

 0

 .00190778375773

 .00190778375773

 .00190778375773

 .00190778375773

 .00190778375773

 .000682785765925

 .000682785765925

 0

 0

 0

 0

 0

 0

 0

 0

 0

 0

 0

 0

 0

 0

 0

 0

 0

 0

 0

 0

 0

 0

 0

 0

 0

 0

 0

 .000682785765925

 0

 0

 .000682785765925

 .000682785765925

 0

 0

 0

 0

 0

 0

 0

 0

 0

 0

 0

 0

 0

 0

 0

 0
