# Supplementary material for: First Insight into Microbiome Profiles of Myrmecophilous Beetles and Their Host, Red Wood Ant Formica polyctena (Hymenoptera: Formicidae)—A Case Study
Source: Insects. 2020 Feb 19;11(2):134. doi: 10.3390/insects11020134 (PMC7073670; doi:10.3390/insects11020134)
Supplement: Supplementary file 1 [file insects-11-00134-s001.zip › Supplementary Files/S2_Figure.zip!/Supplemental_Figure_S2/Supplemental_Figure_S2_MS4.html]

Javascript must be enabled to view this page.

magnitude

 .999999999999972

 .999999999999972

 0

 0

 0

 0

 0

 0

 .087213663881112

 .08557371901117

 .08447460702387

 .08447460702387

 .0561942811284

 0

 .0027041644132

 .000366370662433

 .0179870549033

 .00572236082276

 .000191908442227

 0

 .00130846665155

 0

 0

 0

 0

 0

 0

 .0010991119873

 .0010991119873

 .0010991119873

 0

 0

 0

 0

 0

 0

 0

 0

 0

 0

 0

 0

 0

 0

 0

 0

 0

 0

 0

 0

 0

 0

 0

 0

 0

 0

 0

 .001639944869942

 .001639944869942

 .001639944869942

 .00129102042953

 .000348924440412

 0

 0

 0

 0

 0

 0

 0

 0

 0

 0

 0

 0

 0

 0

 0

 0

 0

 0

 0

 0

 0

 0

 0

 0

 0

 0

 0

 0

 0

 0

 0

 .367644236640458

 0

 0

 0

 0

 .006594671923799

 0

 0

 0

 0

 .001936530644289

 .000942095989114

 .000942095989114

 0

 0

 0

 0

 .000994434655175

 .000994434655175

 .003035642631593

 .000366370662433

 .000366370662433

 .00266927196916

 0

 0

 .00266927196916

 0

 0

 0

 0

 .001622498647917

 .000209354664247

 .000209354664247

 0

 0

 .00141314398367

 .00141314398367

 0

 0

 .348540623527874

 0

 0

 0

 .00413475461889

 .00413475461889

 .00413475461889

 0

 .000244247108289

 .000244247108289

 .000244247108289

 .00568746837872

 .00568746837872

 .00568746837872

 6.08349761859431E-02

 .01516076693592

 0

 0

 .00490238838779

 .00725762836058

 .00300075018755

 6.97848880825E-05

 6.97848880825E-05

 0

 0

 .0242502486087

 .0242502486087

 2.13541757532406E-02

 1.74462220206E-05

 .00472792616759

 0

 .00851375634606

 .00809504701757

 0

 0

 0

 0

 0

 0

 0

 0

 .007501875468863

 0

 0

 .000209354664247

 .000209354664247

 0

 0

 0

 .00544322127043

 0

 .00544322127043

 0

 .000732741324866

 0

 0

 .000732741324866

 .000819972434969

 .000819972434969

 .000296585774351

 0

 .000296585774351

 0

 0

 0

 0

 0

 0

 0

 0

 0

 0

 0

 6.75343254418463E-02

 0

 0

 0

 0

 0

 0

 0

 0

 .00334967462796

 0

 0

 0

 0

 .00334967462796

 0

 0

 0

 0

 .00209354664247

 .00209354664247

 0

 .003995184842726

 .00186674575621

 0

 0

 0

 0

 .00197142308833

 .000157015998186

 2.16856539716793E-02

 3.48924440412E-05

 1.74462220206E-05

 .000296585774351

 .000296585774351

 5.23386660619E-05

 0

 0

 8.72311101031E-05

 1.74462220206E-05

 .0103979483243

 3.48924440412E-05

 .000139569776165

 .0102060398821

 0

 0

 1.74462220206E-05

 0

 0

 0

 0

 8.72311101031E-05

 0

 0

 .034613304488891

 .000715295102845

 .00636787103753

 .00586193059893

 .0215111917514

 .000157015998186

 0

 0

 0

 .00179696086812

 .00179696086812

 0

 0

 3.5764755142234E-03

 3.5764755142234E-03

 .00261693330309

 .000767633768907

 0

 3.48924440412E-05

 .000122123554144

 3.48924440412E-05

 .125909384322731

 5.22863273957502E-02

 .0260995481428

 .00055827910466

 .000802526212949

 3.48924440412E-05

 .0247910814913

 .073623056926981

 .0730473316003

 0

 0

 .000575725326681

 0

 .021999685967985

 .021999685967985

 .00027913955233

 .0028786266334

 .000715295102845

 0

 .00804270835151

 .0100839163279

 0

 .0453776234756

 .0453776234756

 0

 0

 .0453776234756

 5.7398070447831E-03

 5.7398070447831E-03

 8.72311101031E-05

 0

 .00565257593468

 .000750187546887

 .000750187546887

 .000750187546887

 .000750187546887

 0

 0

 0

 0

 0

 0

 .011758753641898

 0

 0

 0

 0

 0

 0

 0

 0

 .011758753641898

 0

 0

 .00172717598004

 .00172717598004

 0

 .010031577661858

 .00392539995464

 .000505940438598

 .00560023726862

 0

 0

 0

 0

 0

 0

 0

 0

 0

 0

 0

 0

 0

 0

 0

 0

 0

 0

 0

 4.75060625621825E-02

 4.75060625621825E-02

 .00226800886268

 0

 0

 .00226800886268

 .00226800886268

 0

 0

 0

 0

 0

 0

 .02390132416825

 .000314031996371

 .000314031996371

 .023587292171879

 0

 .00915926656083

 0

 0

 0

 0

 .00254714841501

 .000540832882639

 0

 0

 0

 0

 0

 0

 0

 .0113400443134

 0

 0

 0

 0

 0

 0

 0

 0

 0

 0

 0

 6.629564367835E-04

 0

 0

 .000593171548701

 0

 .000593171548701

 0

 0

 6.97848880825E-05

 0

 6.97848880825E-05

 0

 0

 .020673773094469

 0

 0

 0

 0

 .020673773094469

 0

 .00235523997278

 .0180742860134

 0

 .000244247108289

 0

 0

 0

 0

 0

 0

 0

 0

 0

 0

 0

 0

 0

 0

 0

 0

 0

 0

 0

 0

 0

 0

 0

 0

 0

 0

 0

 0

 0

 0

 0

 0

 0

 0

 0

 0

 0

 0

 0

 0

 0

 0

 0

 0

 0

 0

 0

 0

 0

 0

 0

 0

 0

 0

 0

 0

 0

 0

 0

 0

 0

 0

 0

 0

 0

 0

 0

 0

 0

 0

 0

 0

 0

 0

 0

 0

 0

 0

 0

 0

 0

 0

 0

 0

 0

 0

 0

 0

 0

 0

 0

 0

 0

 0

 0

 0

 0

 0

 0

 0

 0

 0

 0

 0

 0

 0

 0

 0

 0

 0

 0

 0

 0

 0

 0

 0

 0

 0

 0

 0

 0

 0

 0

 9.55878504510449E-02

 6.11315619602957E-02

 3.42469338264957E-02

 8.72311101031E-05

 8.72311101031E-05

 0

 0

 .00525131282821

 0

 .00525131282821

 0

 0

 0

 0

 0

 0

 0

 0

 0

 0

 8.72311101031E-05

 0

 0

 8.72311101031E-05

 0

 0

 0

 0

 .002616933303097

 .00214588530854

 .00027913955233

 0

 .000191908442227

 6.97848880825E-05

 6.97848880825E-05

 .0261344405869

 0

 0

 .0261344405869

 0

 0

 .0268846281338

 0

 0

 0

 0

 0

 .000331478218392

 0

 .000331478218392

 0

 0

 0

 .000226800886268

 .000226800886268

 0

 0

 .02632634902914

 .00214588530854

 .0241804637206

 2.31860290654212E-02

 2.31860290654212E-02

 1.58237233727112E-02

 .0139569776165

 0

 .00183185331217

 3.48924440412E-05

 0

 0

 0

 .00736230569271

 .00186674575621

 .0054955599365

 0

 0

 0

 0

 0

 0

 0

 0

 0

 0

 0

 0

 0

 0

 0

 0

 0

 0

 0

 0

 0

 0

 0

 0

 0

 0

 0

 0

 0

 .011270259425328

 .011270259425328

 .011270259425328

 0

 0

 .000785079990928

 0

 .0104851794344

 0

 0

 0

 0

 0

 0

 0

 0

 0

 0

 0

 0

 0

 0

 0

 0

 0

 0

 0

 0

 0

 0

 .00188419197823

 .00188419197823

 .00188419197823

 .00188419197823

 .00188419197823

 .01095622742895

 0

 0

 0

 0

 0

 0

 0

 0

 0

 0

 0

 0

 .01095622742895

 .01095622742895

 0

 0

 .00251225597097

 0

 .00251225597097

 0

 0

 0

 0

 .00232034752874

 .00232034752874

 .00612362392924

 .00612362392924

 0

 0

 0

 0

 0

 0

 .046877998569409

 .003157766185733

 0

 0

 0

 0

 .003157766185733

 0

 0

 0

 0

 .003157766185733

 0

 .000645510214763

 .00251225597097

 0

 0

 0

 0

 .043720232383676

 .00462324883546

 .00462324883546

 .00125612798548

 .00336712084998

 .039096983548216

 .039096983548216

 .0138697465064

 .000436155550516

 .0016050524259

 0

 .012683403409

 .0105026256564

 0

 0

 0

 0

 0

 0

 0

 0

 0

 0

 .339922189849743

 .164186395436055

 .015963293148864

 .015963293148864

 0

 0

 .00797292346342

 .00202376175439

 0

 0

 .000383816884454

 0

 0

 0

 0

 0

 .00232034752874

 0

 0

 0

 0

 .00111655820932

 .00214588530854

 0

 0

 0

 0

 0

 0

 .00901969678466

 .00901969678466

 .00472792616759

 0

 0

 0

 .00429177061707

 0

 0

 0

 0

 0

 0

 0

 0

 0

 0

 0

 0

 0

 0

 0

 0

 0

 0

 0

 0

 .01489907360557

 .01489907360557

 0

 .0106073029885

 .00429177061707

 .00170972975802

 .00170972975802

 .00170972975802

 0

 .0387829515518

 .0387829515518

 .0387829515518

 .00258204085905

 .00258204085905

 .00258204085905

 0

 2.98679320993012E-02

 0

 0

 0

 0

 .00317521240775

 0

 0

 0

 0

 .00317521240775

 0

 0

 0

 0

 0

 0

 0

 0

 .00565257593468

 .00190163820025

 0

 .00375093773443

 0

 .00717039725048

 .00717039725048

 0

 0

 0

 0

 0

 0

 0

 0

 0

 0

 0

 1.8492995341847E-03

 6.97848880825E-05

 0

 0

 0

 1.74462220206E-05

 .00115145065336

 0

 0

 1.74462220206E-05

 0

 0

 0

 0

 .000593171548701

 0

 0

 0

 0

 0

 0

 1.20204469722065E-02

 .000209354664247

 .00696104258623

 0

 .000157015998186

 0

 6.97848880825E-05

 0

 .000296585774351

 0

 .00432666306111

 0

 0

 0

 0

 .0095605296673

 .0095605296673

 0

 0

 0

 .00610617770722

 .00209354664247

 0

 0

 0

 0

 .00136080531761

 .0108864425409

 0

 0

 0

 .0108864425409

 .0108864425409

 0

 0

 .001482928871757

 0

 0

 .000209354664247

 .000209354664247

 .00127357420751

 0

 .00127357420751

 0

 2.94317765488331E-02

 2.94317765488331E-02

 3.48924440412E-05

 0

 0

 0

 5.23386660619E-05

 0

 0

 0

 0

 .00982222299761

 .0138348540624

 .00225056264066

 .00343690573806

 0

 0

 0

 0

 0

 0

 0

 0

 0

 0

 0

 .00193653064429

 0

 0

 0

 0

 0

 0

 0

 0

 0

 0

 0

 0

 0

 .00193653064429

 0

 0

 0

 0

 0

 0

 .00193653064429

 .00193653064429

 0

 0

 0

 0

 0

 0

 0

 0

 0

 0

 0

 0

 0

 0

 0

 0

 0

 0

 0

 0

 0

 0

 0

 0

 0

 0

 .173799263769397

 .00331478218392

 .00331478218392

 .00331478218392

 0

 0

 0

 0

 0

 0

 0

 0

 .086864739440661

 0

 0

 .066871369005025

 .0213541757532

 .000976988433155

 0

 0

 .00436155550516

 .000819972434969

 .0163122175893

 0

 0

 .00812993946161

 0

 0

 0

 0

 0

 0

 0

 .00975243810953

 .00485004972173

 5.23386660619E-05

 0

 0

 0

 0

 1.74462220206E-05

 .000174462220206

 0

 0

 0

 1.74462220206E-05

 0

 0

 0

 0

 0

 5.23386660619E-05

 0

 0

 0

 0

 0

 0

 0

 0

 .00730996702664

 0

 .00730996702664

 0

 0

 0

 0

 0

 0

 .012683403408996

 .00579214571085

 .000436155550516

 .00186674575621

 .00458835639142

 0

 0

 0

 0

 0

 .00765889146705

 0

 0

 .00765889146705

 .00765889146705

 .000767633768907

 .000767633768907

 .000505940438598

 0

 .000261693330309

 9.944346551755E-04

 9.944346551755E-04

 .000523386660619

 0

 0

 0

 .000401263106474

 0

 1.74462220206E-05

 0

 0

 0

 0

 0

 0

 0

 5.23386660619E-05

 0

 0

 0

 0

 0

 .002651825747135

 .002651825747135

 .000139569776165

 .00251225597097

 .00209354664247

 .00209354664247

 .00209354664247

 .00163994486994

 .00163994486994

 .00163994486994

 0

 0

 0

 1.74462220206E-05

 1.74462220206E-05

 1.74462220206E-05

 0

 0

 0

 0

 0

 .00471047994557

 .00471047994557

 .00471047994557

 2.7565030792581E-03

 1.3433590955881E-03

 .000575725326681

 0

 8.72311101031E-05

 0

 0

 .000680402658804

 .00141314398367

 .00141314398367

 8.72311101031E-05

 8.72311101031E-05

 8.72311101031E-05

 0

 0

 0

 0

 0

 0

 0

 0

 0

 0

 0

 0

 0

 0

 0

 0

 .060241804637187

 .055723233133843

 3.48924440412E-05

 .00163994486994

 0

 3.48924440412E-05

 1.74462220206E-05

 0

 .0539960571538

 .004518571503344

 0

 0

 0

 .00357647551423

 .000942095989114

 0

 0

 0

 0

 0

 0

 0

 0

 0

 0

 0

 0

 0

 6.97848880825E-05

 6.97848880825E-05

 6.97848880825E-05

 6.97848880825E-05

 6.97848880825E-05

 2.3377937507606E-03

 2.3377937507606E-03

 1.74462220206E-05

 0

 0

 0

 0

 0

 1.74462220206E-05

 1.74462220206E-05

 0

 0

 0

 .00232034752874

 .00232034752874

 0

 0

 .00232034752874

 0

 0

 0

 0

 0

 0

 0

 0

 0

 0

 0

 0

 0

 0

 0

 0

 0

 0

 0

 0

 0

 0

 0

 0

 0

 0

 0

 0

 0

 0

 0

 0
