# Supplementary material for: First Insight into Microbiome Profiles of Myrmecophilous Beetles and Their Host, Red Wood Ant Formica polyctena (Hymenoptera: Formicidae)—A Case Study
Source: Insects. 2020 Feb 19;11(2):134. doi: 10.3390/insects11020134 (PMC7073670; doi:10.3390/insects11020134)
Supplement: Supplementary file 1 [file insects-11-00134-s001.zip › Supplementary Files/S2_Figure.zip!/Supplemental_Figure_S2/Supplemental_Figure_S2_PF1.html]

Javascript must be enabled to view this page.

magnitude

 .999999999999597

 .999999999999597

 5.99538355466E-05

 5.99538355466E-05

 5.99538355466E-05

 5.99538355466E-05

 0

 5.99538355466E-05

 2.99769177733E-05

 2.99769177733E-05

 0

 0

 0

 0

 0

 0

 0

 0

 0

 0

 0

 0

 0

 0

 0

 0

 0

 0

 0

 0

 0

 0

 0

 0

 0

 2.99769177733E-05

 2.99769177733E-05

 2.99769177733E-05

 0

 0

 0

 0

 0

 0

 0

 0

 0

 0

 0

 0

 0

 0

 0

 0

 0

 0

 0

 0

 0

 0

 0

 0

 0

 0

 0

 0

 0

 0

 0

 0

 0

 0

 0

 0

 0

 0

 0

 0

 0

 0

 0

 0

 0

 0

 0

 0

 0

 0

 0

 0

 0

 0

 0

 1.21556401570823E-02

 0

 0

 0

 0

 0

 0

 0

 0

 0

 0

 0

 0

 0

 0

 0

 0

 0

 0

 0

 0

 0

 0

 0

 0

 0

 0

 0

 0

 0

 0

 0

 0

 0

 0

 0

 0

 0

 0

 1.17959171438023E-02

 0

 0

 0

 0

 0

 0

 0

 0

 0

 0

 0

 0

 0

 .007329356395575

 .001199076710932

 0

 0

 .000554572978806

 .000254803801073

 .000389699931053

 0

 0

 0

 0

 .000794388320993

 .000794388320993

 .0006744806499

 0

 .00040468838994

 0

 .00026979225996

 0

 0

 0

 0

 0

 .00466141071375

 .00466141071375

 0

 0

 5.99538355466E-05

 0

 0

 5.99538355466E-05

 5.99538355466E-05

 0

 0

 0

 0

 0

 0

 0

 0

 0

 0

 0

 0

 0

 0

 0

 0

 0

 0

 0

 0

 0

 0

 0

 0

 0

 0

 0

 1.5887766419853E-03

 0

 0

 0

 0

 0

 0

 0

 0

 .000119907671093

 0

 0

 0

 0

 .000119907671093

 0

 0

 0

 0

 0

 0

 0

 0

 0

 0

 0

 0

 0

 0

 0

 2.99769177733E-05

 0

 0

 0

 0

 0

 0

 0

 0

 0

 0

 0

 0

 2.99769177733E-05

 0

 0

 0

 0

 0

 0

 0

 0

 0

 0

 .001438892053119

 0

 0

 .00049461914326

 .000944272909859

 0

 0

 0

 0

 0

 0

 0

 0

 1.49884588867E-05

 1.49884588867E-05

 0

 1.49884588867E-05

 0

 0

 0

 0

 2.4281303396427E-03

 .000374711472166

 0

 0

 0

 0

 .000374711472166

 2.0534188674767E-03

 .00203843040859

 0

 0

 1.49884588867E-05

 0

 0

 0

 0

 0

 0

 0

 0

 0

 0

 .000374711472166

 .000374711472166

 0

 0

 .000374711472166

 0

 0

 0

 0

 0

 0

 0

 0

 0

 0

 0

 0

 0

 0

 0

 .00035972301328

 .000164873047753

 0

 0

 .000164873047753

 .000164873047753

 0

 0

 0

 .000194849965527

 0

 0

 .000194849965527

 .000194849965527

 0

 0

 0

 0

 0

 0

 0

 0

 0

 0

 0

 0

 0

 0

 0

 0

 0

 0

 0

 0

 0

 0

 0

 0

 .000509607602147

 .000509607602147

 1.49884588867E-05

 0

 0

 0

 0

 1.49884588867E-05

 0

 1.49884588867E-05

 0

 0

 0

 2.99769177733E-05

 0

 0

 2.99769177733E-05

 0

 2.99769177733E-05

 0

 0

 0

 0

 0

 0

 0

 0

 0

 0

 0

 0

 0

 0

 .00004496537666

 0

 0

 0

 0

 .00004496537666

 0

 .00004496537666

 0

 0

 0

 .000239815342187

 0

 0

 0

 0

 0

 0

 0

 .000239815342187

 0

 .000239815342187

 0

 0

 .00017986150664

 0

 0

 0

 0

 .00017986150664

 0

 0

 0

 .00017986150664

 0

 0

 0

 0

 0

 0

 0

 0

 0

 0

 0

 0

 0

 0

 0

 0

 0

 0

 0

 0

 0

 0

 0

 0

 0

 0

 0

 0

 0

 0

 0

 0

 0

 0

 0

 0

 0

 0

 0

 0

 0

 0

 0

 0

 0

 0

 0

 0

 0

 0

 0

 0

 0

 0

 0

 0

 0

 0

 0

 0

 0

 0

 0

 0

 0

 0

 0

 0

 0

 0

 0

 0

 8.99307533199E-05

 0

 0

 0

 0

 8.99307533199E-05

 8.99307533199E-05

 8.99307533199E-05

 8.99307533199E-05

 0

 0

 0

 0

 0

 0

 0

 0

 0

 0

 0

 0

 0

 0

 0

 0

 0

 0

 0

 0

 0

 0

 0

 0

 0

 0

 0

 0

 0

 0

 0

 0

 0

 0

 0

 0

 0

 0

 0

 0

 0

 0

 0

 4.1817800293788E-03

 3.6721724272319E-03

 .000509607602146

 0

 0

 0

 0

 0

 0

 0

 0

 0

 0

 0

 0

 0

 0

 0

 0

 0

 0

 0

 0

 0

 0

 0

 0

 0

 0

 0

 0

 0

 0

 0

 0

 .000509607602146

 0

 0

 .000509607602146

 0

 0

 3.1625648250859E-03

 0

 0

 0

 0

 0

 0

 0

 0

 0

 0

 0

 0

 0

 8.99307533199E-05

 8.99307533199E-05

 .003072634071766

 .00274288797626

 .000329746095506

 .000419676848827

 .000419676848827

 .00013489612998

 .00013489612998

 0

 0

 0

 0

 0

 0

 .00013489612998

 0

 0

 0

 0

 .00013489612998

 0

 0

 0

 0

 0

 0

 0

 0

 0

 0

 0

 0

 0

 .000149884588867

 0

 0

 .000149884588867

 0

 0

 0

 0

 0

 0

 0

 0

 0

 8.99307533199E-05

 8.99307533199E-05

 8.99307533199E-05

 0

 0

 0

 0

 8.99307533199E-05

 0

 0

 0

 0

 0

 0

 0

 0

 0

 0

 0

 0

 0

 0

 0

 0

 0

 0

 0

 0

 0

 0

 0

 0

 0

 0

 0

 .00004496537666

 0

 0

 0

 0

 0

 0

 0

 0

 0

 0

 0

 0

 .00004496537666

 .00004496537666

 0

 0

 0

 0

 0

 0

 0

 0

 0

 0

 0

 .00004496537666

 .00004496537666

 0

 0

 0

 0

 0

 0

 0

 0

 0

 0

 0

 0

 0

 0

 0

 0

 0

 0

 0

 0

 0

 0

 0

 0

 0

 0

 0

 0

 0

 0

 0

 0

 0

 0

 0

 0

 0

 0

 0

 0

 0

 0

 0

 0

 0

 0

 0

 0

 .982928145327689

 .915330195748867

 0

 0

 0

 0

 0

 0

 0

 0

 0

 0

 0

 0

 0

 0

 0

 0

 0

 0

 0

 0

 0

 0

 0

 0

 0

 0

 0

 1.948499655265E-04

 1.948499655265E-04

 2.99769177733E-05

 0

 7.49422944333E-05

 0

 8.99307533199E-05

 0

 0

 0

 0

 0

 0

 0

 0

 0

 0

 0

 0

 0

 0

 0

 0

 0

 0

 0

 0

 .00004496537666

 .00004496537666

 0

 .00004496537666

 0

 0

 0

 0

 0

 0

 0

 0

 0

 0

 0

 0

 .00121406516982

 0

 0

 0

 0

 .000434665307713

 0

 0

 0

 .00004496537666

 .000389699931053

 0

 0

 0

 0

 0

 0

 0

 0

 0

 0

 0

 0

 0

 0

 0

 0

 0

 0

 0

 0

 .000149884588867

 .000149884588867

 0

 0

 0

 0

 3.447345543933E-04

 1.49884588867E-05

 .00026979225996

 0

 0

 0

 0

 0

 5.99538355466E-05

 0

 0

 0

 0

 0

 0

 0

 0

 0

 0

 0

 0

 2.847807188467E-04

 .00004496537666

 .0002248268833

 0

 0

 0

 0

 0

 0

 1.49884588867E-05

 0

 0

 0

 0

 0

 .000734434485447

 .000734434485447

 .000104919212207

 .00004496537666

 0

 .000194849965527

 .000389699931053

 0

 0

 0

 0

 0

 0

 0

 0

 0

 0

 0

 0

 0

 .912932042327

 0

 0

 .191417608441

 .191417608441

 .721514433886

 0

 .721514433886

 0

 7.49422944333E-05

 7.49422944333E-05

 0

 0

 0

 0

 0

 0

 0

 0

 0

 0

 5.99538355466E-05

 0

 1.49884588867E-05

 0

 .00013489612998

 .00013489612998

 .00013489612998

 0

 0

 0

 0

 0

 0

 0

 0

 0

 0

 0

 0

 0

 0

 0

 0

 0

 0

 0

 0

 0

 0

 0

 0

 0

 0

 0

 0

 0

 0

 0

 0

 0

 0

 0

 0

 0

 0

 0

 0

 0

 0

 0

 0

 0

 0

 0

 0

 0

 0

 0

 0

 0

 0

 0

 0

 6.75979495788225E-02

 0

 0

 0

 0

 0

 0

 0

 0

 0

 0

 0

 2.8328187295759E-03

 0

 0

 2.5180610929562E-03

 .00191852273749

 .00017986150664

 0

 5.99538355466E-05

 2.99769177733E-05

 0

 1.49884588867E-05

 0

 .000164873047753

 5.99538355466E-05

 0

 0

 2.99769177733E-05

 0

 0

 0

 .00004496537666

 0

 0

 0

 0

 0

 0

 0

 0

 0

 0

 0

 0

 1.49884588867E-05

 0

 0

 0

 0

 0

 0

 0

 0

 0

 0

 0

 1.49884588867E-05

 1.49884588867E-05

 0

 0

 0

 0

 0

 0

 0

 0

 0

 0

 .000299769177733

 0

 0

 0

 .000299769177733

 0

 0

 0

 0

 0

 0

 0

 0

 0

 0

 0

 0

 0

 0

 0

 6.32512965016937E-02

 6.32512965016937E-02

 .0409484696783

 0

 0

 0

 1.49884588867E-05

 0

 .000104919212207

 0

 0

 0

 0

 0

 0

 0

 .021958092269

 0

 0

 0

 .0002248268833

 0

 1.49884588867E-05

 1.49884588867E-05

 0

 1.49884588867E-05

 0

 0

 0

 0

 0

 0

 0

 0

 0

 0

 0

 0

 0

 0

 0

 0

 0

 0

 0

 0

 2.847807188462E-04

 2.548038010729E-04

 2.99769177733E-05

 .000164873047753

 0

 5.99538355466E-05

 0

 0

 2.99769177733E-05

 2.99769177733E-05

 0

 0

 0

 0

 0

 0

 0

 0

 0

 0

 0

 0

 0

 0

 0

 0

 0

 0

 0

 .00121406516982

 0

 0

 0

 0

 0

 0

 0

 0

 .00121406516982

 0

 0

 0

 0

 0

 .00121406516982

 0

 0

 0

 0

 0

 0

 0

 0

 0

 0

 0

 0

 0

 0

 0

 0

 0

 0

 0

 0

 0

 0

 0

 0

 0

 0

 0

 0

 0

 0

 0

 0

 0

 0

 0

 0

 0

 0

 0

 0

 0

 0

 0

 0

 0

 0

 0

 0

 0

 0

 0

 0

 0

 0

 0

 0

 0

 0

 0

 0

 0

 0

 0

 0

 0

 0

 0
