# Supplementary material for: First Insight into Microbiome Profiles of Myrmecophilous Beetles and Their Host, Red Wood Ant Formica polyctena (Hymenoptera: Formicidae)—A Case Study
Source: Insects. 2020 Feb 19;11(2):134. doi: 10.3390/insects11020134 (PMC7073670; doi:10.3390/insects11020134)
Supplement: Supplementary file 1 [file insects-11-00134-s001.zip › Supplementary Files/S2_Figure.zip!/Supplemental_Figure_S2/Supplemental_Figure_S2_PF2.html]

Javascript must be enabled to view this page.

magnitude

 1.00000000000028

 1.00000000000028

 .000440255348102

 .000440255348102

 .000440255348102

 .000440255348102

 0

 .000440255348102

 .001280742830843

 3.001741009785E-04

 2.801624942466E-04

 2.201276740509E-04

 0

 0

 0

 0

 0

 2.00116067319E-05

 0

 0

 .000200116067319

 0

 0

 6.00348201957E-05

 6.00348201957E-05

 0

 0

 2.00116067319E-05

 2.00116067319E-05

 2.00116067319E-05

 0

 0

 0

 0

 0

 0

 0

 0

 0

 0

 0

 0

 .000220127674051

 0

 0

 0

 0

 0

 0

 0

 0

 0

 0

 0

 .000220127674051

 .000220127674051

 .000220127674051

 .000400232134639

 .000400232134639

 .000400232134639

 .000300174100979

 .00010005803366

 0

 0

 0

 0

 0

 0

 0

 0

 0

 4.00232134638E-05

 4.00232134638E-05

 4.00232134638E-05

 4.00232134638E-05

 6.00348201957E-05

 6.00348201957E-05

 6.00348201957E-05

 6.00348201957E-05

 0

 0

 0

 0

 .000260150887515

 .000260150887515

 .000260150887515

 .000260150887515

 0

 0

 0

 0

 0

 0

 1.65495987672806E-02

 0

 0

 0

 0

 2.801624942462E-04

 0

 0

 0

 0

 0

 0

 0

 0

 0

 0

 0

 0

 0

 2.801624942462E-04

 8.00464269276E-05

 8.00464269276E-05

 1.600928538548E-04

 .000120069640391

 4.00232134638E-05

 0

 0

 0

 4.00232134638E-05

 4.00232134638E-05

 0

 0

 0

 0

 0

 0

 0

 0

 0

 1.59892737787878E-02

 0

 0

 0

 .000140081247123

 .000140081247123

 .000140081247123

 0

 0

 0

 0

 0

 0

 0

 3.6421124252078E-03

 .002021172279923

 0

 0

 .000360208921174

 .00104060355006

 .000620359808689

 0

 0

 4.00232134638E-05

 4.00232134638E-05

 .00042024374137

 .00042024374137

 .001160673190451

 0

 .000280162494247

 0

 0

 .000880510696204

 0

 0

 0

 0

 0

 0

 0

 0

 5.002901682969E-04

 0

 0

 .000340197314442

 .000340197314442

 0

 0

 0

 0

 0

 0

 0

 .000140081247123

 0

 .000140081247123

 0

 0

 0

 0

 0

 0

 2.00116067319E-05

 2.00116067319E-05

 0

 0

 2.00116067319E-05

 2.00116067319E-05

 0

 0

 0

 2.00116067319E-05

 0

 2.2012767405092E-03

 2.00116067319E-05

 2.00116067319E-05

 0

 0

 .000240139280783

 .000240139280783

 0

 0

 .00010005803366

 0

 0

 0

 0

 .00010005803366

 0

 0

 0

 0

 2.00116067319E-05

 0

 2.00116067319E-05

 .000200116067319

 0

 0

 0

 0

 0

 .000200116067319

 0

 1.400812471229E-04

 0

 0

 0

 0

 0

 0

 0

 0

 2.00116067319E-05

 0

 0

 0

 .000120069640391

 0

 0

 0

 0

 0

 0

 0

 0

 0

 0

 1.3808008645005E-03

 .000160092853855

 .000540313381761

 6.00348201957E-05

 .000580336595225

 4.00232134638E-05

 0

 0

 0

 0

 0

 .00010005803366

 .00010005803366

 0

 0

 0

 0

 0

 0

 0

 0

 .008945188209157

 .000920533909667

 0

 0

 .000180104460587

 0

 .00074042944908

 .00802465429949

 .00802465429949

 0

 0

 0

 0

 .000240139280783

 .000240139280783

 0

 0

 .00010005803366

 0

 0

 .000140081247123

 0

 .000300174100979

 .000300174100979

 0

 0

 .000300174100979

 0

 0

 0

 0

 0

 0

 0

 0

 0

 0

 0

 8.00464269276E-05

 8.00464269276E-05

 8.00464269276E-05

 8.00464269276E-05

 .000200116067319

 .000200116067319

 0

 0

 .000200116067319

 0

 0

 0

 .000200116067319

 0

 0

 0

 0

 0

 0

 0

 0

 0

 0

 0

 0

 0

 0

 0

 0

 0

 0

 0

 0

 0

 0

 0

 0

 0

 0

 0

 0

 0

 1.0606151567905E-03

 1.0606151567905E-03

 0

 0

 0

 0

 0

 0

 0

 0

 0

 0

 0

 3.602089211737E-04

 0

 0

 3.602089211737E-04

 0

 0

 0

 0

 0

 6.00348201957E-05

 0

 0

 0

 0

 0

 0

 0

 .000120069640391

 0

 .000180104460587

 4.00232134638E-05

 0

 0

 0

 0

 4.00232134638E-05

 0

 4.00232134638E-05

 0

 0

 0

 .00042024374137

 0

 0

 .000300174100979

 0

 .000300174100979

 0

 0

 .000120069640391

 0

 .000120069640391

 0

 0

 .000240139280783

 0

 0

 0

 0

 .000240139280783

 0

 0

 .000240139280783

 0

 0

 0

 0

 0

 0

 0

 0

 0

 0

 0

 0

 0

 0

 0

 0

 9.205339096674E-04

 0

 0

 0

 0

 4.002321346384E-04

 0

 0

 0

 3.602089211746E-04

 3.602089211746E-04

 .000280162494247

 8.00464269276E-05

 4.00232134638E-05

 4.00232134638E-05

 4.00232134638E-05

 0

 0

 0

 .000140081247123

 0

 0

 0

 0

 0

 .000140081247123

 .000140081247123

 0

 .000140081247123

 0

 0

 0

 0

 0

 0

 .000240139280783

 .000240139280783

 .000240139280783

 .000240139280783

 .000140081247123

 .000140081247123

 .000140081247123

 .000140081247123

 0

 0

 0

 0

 0

 0

 0

 0

 0

 0

 0

 0

 0

 0

 .00042024374137

 0

 0

 0

 0

 .00032018570771

 .000120069640391

 0

 0

 .000120069640391

 .000120069640391

 .000200116067319

 .000200116067319

 .000200116067319

 .00010005803366

 .00010005803366

 .00010005803366

 .00010005803366

 0

 0

 0

 0

 0

 0

 0

 0

 0

 0

 0

 0

 0

 0

 0

 0

 0

 0

 0

 0

 0

 0

 0

 0

 0

 0

 0

 0

 0

 0

 0

 0

 0

 0

 3.9823097396449E-03

 3.8022052790582E-03

 1.6809749654792E-03

 0

 0

 0

 0

 .000360208921174

 0

 .000360208921174

 0

 0

 0

 .000140081247123

 .000140081247123

 8.00464269276E-05

 8.00464269276E-05

 0

 0

 0

 2.201276740506E-04

 .000140081247123

 8.00464269276E-05

 0

 0

 0

 0

 0

 0

 0

 0

 0

 0

 0

 0

 .000880510696204

 0

 0

 .000880510696204

 0

 0

 .002121230313579

 0

 0

 0

 0

 0

 0

 0

 0

 0

 0

 0

 0

 0

 0

 0

 .002121230313579

 .00192111424626

 .000200116067319

 1.801044605867E-04

 1.801044605867E-04

 0

 0

 0

 0

 0

 0

 0

 0

 .000120069640391

 .000120069640391

 0

 0

 0

 0

 0

 0

 0

 6.00348201957E-05

 0

 0

 0

 0

 0

 6.00348201957E-05

 0

 0

 0

 0

 0

 0

 0

 0

 0

 0

 0

 0

 0

 0

 0

 0

 0

 0

 0

 0

 0

 0

 0

 0

 .00052030177503

 .00052030177503

 .00052030177503

 0

 0

 .00052030177503

 .00052030177503

 .000500290168297

 .000500290168297

 .000500290168297

 .000500290168297

 .000340197314442

 .000160092853855

 0

 0

 0

 0

 0

 0

 0

 0

 0

 .000220127674051

 .000220127674051

 .000220127674051

 .000220127674051

 .000220127674051

 .000580336595225

 0

 0

 0

 0

 0

 0

 0

 0

 0

 0

 0

 0

 .000580336595225

 .000580336595225

 .000260150887515

 .000260150887515

 0

 0

 0

 0

 0

 0

 0

 0

 0

 .00032018570771

 .00032018570771

 0

 0

 0

 0

 0

 0

 3.001741009787E-04

 0

 0

 0

 0

 0

 0

 0

 0

 0

 0

 0

 0

 0

 0

 0

 0

 0

 0

 3.001741009787E-04

 4.00232134638E-05

 4.00232134638E-05

 4.00232134638E-05

 0

 0

 0

 0

 0

 0

 0

 0

 0

 .000240139280783

 .000240139280783

 0

 .000240139280783

 2.00116067319E-05

 0

 0

 2.00116067319E-05

 2.00116067319E-05

 0

 .972083808609285

 .953272898281299

 .000820475876008

 .000820475876008

 0

 0

 0

 0

 .000120069640391

 0

 0

 0

 0

 0

 0

 0

 0

 .000200116067319

 0

 0

 .000220127674051

 .000280162494247

 0

 0

 0

 0

 0

 0

 0

 .001680974965481

 .001680974965481

 .000300174100979

 0

 .000700406235617

 0

 .00052030177503

 .000160092853855

 0

 0

 0

 0

 .000200116067319

 .000200116067319

 .000200116067319

 4.00232134638E-05

 4.00232134638E-05

 0

 0

 0

 0

 0

 4.00232134638E-05

 0

 0

 0

 0

 0

 0

 0

 0

 0

 0

 0

 0

 0

 .00032018570771

 .00032018570771

 .00032018570771

 0

 0

 0

 0

 2.5014508414871E-03

 0

 0

 0

 0

 4.002321346381E-04

 2.00116067319E-05

 0

 0

 0

 .000220127674051

 0

 8.00464269276E-05

 0

 0

 0

 8.00464269276E-05

 0

 0

 .000300174100978

 .000140081247123

 0

 .000160092853855

 0

 2.00116067319E-05

 2.00116067319E-05

 0

 0

 0

 0

 0

 0

 0

 0

 0

 0

 0

 5.002901682977E-04

 0

 .000280162494247

 0

 0

 0

 .000160092853855

 0

 0

 0

 0

 6.00348201957E-05

 0

 0

 0

 0

 0

 0

 0

 0

 0

 9.605571231314E-04

 .000300174100979

 .00032018570771

 0

 0

 0

 4.00232134638E-05

 8.00464269276E-05

 0

 0

 .000220127674051

 .000200116067319

 .000200116067319

 .000120069640391

 .000120069640391

 .001240719617378

 .001240719617378

 0

 0

 0

 .000540313381761

 .000700406235617

 0

 0

 0

 0

 0

 1.200696403914E-04

 1.200696403914E-04

 8.00464269276E-05

 4.00232134638E-05

 0

 0

 0

 0

 .942626723499928

 0

 0

 .942546677073

 .942546677073

 8.00464269276E-05

 0

 8.00464269276E-05

 0

 3.7221588521333E-03

 3.7221588521333E-03

 .000120069640391

 .00010005803366

 0

 0

 0

 .000240139280783

 0

 6.00348201957E-05

 8.00464269276E-05

 .00162094014528

 .00112064997699

 .000220127674051

 .000160092853855

 0

 0

 0

 0

 0

 0

 0

 0

 0

 0

 0

 .001140661583719

 .000120069640391

 .000120069640391

 .000120069640391

 .000580336595226

 .000280162494247

 .00010005803366

 .000180104460587

 .000300174100979

 .000300174100979

 0

 .000180104460587

 .000180104460587

 .000180104460587

 .000260150887515

 0

 0

 0

 0

 0

 0

 0

 0

 0

 0

 0

 0

 .00010005803366

 .00010005803366

 0

 .000160092853855

 .000160092853855

 0

 0

 0

 0

 0

 0

 0

 0

 0

 0

 0

 0

 0

 0

 0

 0

 0

 1.76702487442672E-02

 .000220127674051

 .000220127674051

 .000220127674051

 0

 0

 0

 0

 0

 0

 0

 0

 4.6226811550685E-03

 0

 0

 3.8622400992572E-03

 .000480278561566

 .000180104460587

 0

 0

 .000380220527906

 0

 6.00348201957E-05

 0

 .000140081247123

 .000280162494247

 0

 0

 0

 .000200116067319

 0

 0

 6.00348201957E-05

 .000380220527906

 0

 0

 0

 0

 .000400232134638

 0

 0

 4.00232134638E-05

 .000600348201957

 .000220127674051

 0

 0

 0

 0

 .000200116067319

 0

 0

 .000240139280783

 0

 0

 0

 0

 0

 0

 0

 0

 .000140081247123

 0

 .000140081247123

 3.802205279058E-04

 0

 0

 .000180104460587

 4.00232134638E-05

 .000160092853855

 4.00232134638E-05

 0

 4.00232134638E-05

 0

 0

 .000140081247123

 0

 .000140081247123

 6.00348201957E-05

 6.00348201957E-05

 .0046426927618

 0

 0

 .0046426927618

 .0046426927618

 0

 0

 0

 0

 0

 2.1212303135774E-03

 2.1212303135774E-03

 2.00116067319E-05

 0

 0

 2.00116067319E-05

 .000400232134638

 0

 8.00464269276E-05

 0

 0

 0

 0

 0

 0

 .000400232134638

 .00120069640391

 0

 0

 0

 0

 0

 .00010005803366

 .00010005803366

 0

 .00010005803366

 .00102059194333

 .00102059194333

 .00102059194333

 0

 0

 0

 .000140081247123

 .000140081247123

 .000140081247123

 4.00232134638E-05

 0

 0

 4.00232134638E-05

 4.00232134638E-05

 0

 0

 0

 .000120069640391

 .000120069640391

 .000120069640391

 1.4408356846969E-03

 8.805106962039E-04

 2.00116067319E-05

 0

 0

 0

 0

 .000860499089472

 .000560324988493

 .000560324988493

 0

 0

 0

 0

 0

 0

 0

 0

 0

 0

 0

 0

 0

 0

 0

 0

 0

 0

 0

 3.2018570771056E-03

 4.802785615656E-04

 8.00464269276E-05

 0

 0

 0

 0

 0

 .000400232134638

 .00272157851554

 0

 .00166096335875

 .000380220527906

 .000340197314442

 .000340197314442

 0

 0

 0

 0

 0

 0

 0

 0

 0

 0

 0

 0

 0

 0

 0

 0

 0

 0

 1.1406615837188E-03

 1.1406615837188E-03

 .00042024374137

 .000220127674051

 .000220127674051

 0

 .000200116067319

 .000200116067319

 0

 0

 0

 0

 0

 1.600928538552E-04

 8.00464269276E-05

 0

 0

 8.00464269276E-05

 8.00464269276E-05

 8.00464269276E-05

 0

 0

 0

 0

 0

 0

 0

 0

 0

 5.603249884936E-04

 0

 0

 0

 0

 .000480278561566

 .000480278561566

 8.00464269276E-05

 0

 4.00232134638E-05

 4.00232134638E-05

 0

 0

 0

 0

 0

 0

 0

 0

 0

 0
