# Supplementary material for: First Insight into Microbiome Profiles of Myrmecophilous Beetles and Their Host, Red Wood Ant Formica polyctena (Hymenoptera: Formicidae)—A Case Study
Source: Insects. 2020 Feb 19;11(2):134. doi: 10.3390/insects11020134 (PMC7073670; doi:10.3390/insects11020134)
Supplement: Supplementary file 1 [file insects-11-00134-s001.zip › Supplementary Files/S2_Figure.zip!/Supplemental_Figure_S2/Supplemental_Figure_S2_PF3.html]

Javascript must be enabled to view this page.

magnitude

 .999999999999798

 .999999999999798

 0

 0

 0

 0

 0

 0

 4.841489629526E-04

 9.68297925906E-05

 3.87319170362E-05

 0

 0

 0

 0

 0

 0

 0

 0

 0

 0

 0

 0

 3.87319170362E-05

 3.87319170362E-05

 0

 0

 5.80978755544E-05

 5.80978755544E-05

 5.80978755544E-05

 0

 0

 0

 0

 0

 0

 0

 0

 0

 0

 0

 0

 .000387319170362

 0

 0

 0

 0

 0

 .000387319170362

 .000387319170362

 .000387319170362

 0

 0

 0

 0

 0

 0

 0

 0

 0

 0

 0

 0

 0

 0

 0

 0

 0

 0

 0

 0

 0

 0

 0

 0

 0

 0

 0

 0

 0

 0

 0

 0

 0

 0

 0

 0

 0

 0

 0

 0

 0

 0

 2.09539671165955E-02

 0

 0

 0

 0

 7.74638340725E-05

 0

 0

 0

 0

 0

 0

 0

 0

 0

 0

 0

 0

 0

 0

 0

 0

 0

 0

 0

 0

 0

 0

 0

 0

 7.74638340725E-05

 1.93659585181E-05

 1.93659585181E-05

 0

 0

 5.80978755544E-05

 5.80978755544E-05

 0

 0

 .020876503282523

 0

 0

 0

 .000580978755544

 .000580978755544

 .000580978755544

 0

 0

 0

 0

 0

 0

 0

 3.0985533628998E-03

 2.3626469392117E-03

 7.74638340725E-05

 3.87319170362E-05

 .000658442589616

 .00104576175998

 .000542246838507

 0

 0

 0

 0

 .00071654046517

 .00071654046517

 0

 0

 0

 0

 0

 0

 0

 0

 0

 0

 1.93659585181E-05

 1.93659585181E-05

 0

 0

 9.489319673884E-04

 0

 0

 .000251757460736

 .000251757460736

 0

 0

 0

 .000639076631098

 0

 .000639076631098

 0

 5.80978755544E-05

 5.80978755544E-05

 0

 0

 0

 0

 0

 0

 0

 0

 0

 0

 0

 0

 0

 0

 0

 0

 0

 0

 3.4084086991913E-03

 0

 0

 0

 0

 0

 0

 0

 0

 0

 0

 0

 0

 0

 0

 0

 0

 0

 0

 1.93659585181E-05

 0

 1.93659585181E-05

 0

 0

 0

 0

 0

 0

 0

 0

 0

 0

 0

 0

 0

 0

 0

 0

 0

 0

 0

 0

 0

 0

 0

 0

 0

 0

 0

 0

 0

 0

 0

 0

 3.3890427406732E-03

 0

 3.87319170362E-05

 .000232391502217

 .00311791932142

 0

 0

 0

 0

 0

 0

 0

 0

 0

 0

 0

 0

 0

 0

 0

 0

 1.28202645389814E-02

 .000135561709627

 0

 0

 0

 0

 .000135561709627

 1.26847028293544E-02

 .0126266049538

 0

 0

 5.80978755544E-05

 0

 0

 0

 0

 0

 0

 0

 0

 0

 0

 1.93659585181E-05

 1.93659585181E-05

 0

 0

 1.93659585181E-05

 0

 0

 0

 0

 0

 0

 0

 0

 0

 0

 0

 0

 0

 0

 0

 0

 0

 0

 0

 0

 0

 0

 0

 0

 0

 0

 0

 0

 0

 0

 0

 0

 0

 0

 0

 0

 0

 0

 0

 0

 0

 0

 0

 0

 0

 0

 0

 0

 0

 0

 0

 0

 0

 6.971745066527E-04

 6.971745066527E-04

 7.74638340725E-05

 0

 0

 0

 0

 7.74638340725E-05

 7.74638340725E-05

 0

 0

 0

 0

 0

 0

 0

 0

 0

 0

 0

 0

 0

 0

 0

 0

 0

 0

 0

 0

 0

 0

 0

 0

 0

 0

 0

 0

 0

 0

 0

 0

 0

 0

 0

 6.197106725802E-04

 0

 0

 .000329221294808

 0

 .000329221294808

 0

 0

 2.904893777722E-04

 0

 3.87319170362E-05

 .000251757460736

 0

 0

 0

 0

 0

 0

 0

 0

 0

 0

 0

 0

 0

 0

 0

 0

 0

 0

 0

 0

 0

 0

 0

 0

 0

 0

 0

 0

 0

 0

 0

 0

 0

 0

 0

 0

 0

 0

 0

 0

 0

 0

 0

 0

 0

 0

 0

 0

 0

 0

 0

 0

 0

 0

 0

 0

 0

 0

 0

 0

 0

 0

 0

 0

 0

 0

 0

 0

 0

 0

 0

 0

 0

 0

 0

 0

 0

 0

 0

 0

 0

 0

 0

 0

 0

 0

 0

 0

 0

 0

 0

 0

 0

 0

 0

 0

 0

 0

 0

 0

 0

 0

 0

 0

 .000174293626663

 .000174293626663

 .000174293626663

 .000174293626663

 .000174293626663

 0

 0

 0

 0

 0

 0

 0

 0

 0

 0

 0

 0

 0

 0

 0

 0

 0

 0

 0

 0

 0

 0

 0

 0

 0

 0

 7.5139919050282E-03

 6.1777407672782E-03

 5.6161279702532E-03

 1.93659585181E-05

 1.93659585181E-05

 0

 0

 0

 0

 0

 0

 0

 0

 0

 0

 0

 0

 0

 0

 0

 1.93659585181E-05

 0

 0

 0

 1.93659585181E-05

 0

 0

 0

 0

 0

 0

 0

 0

 0

 0

 .005577396053217

 .000135561709627

 0

 .00544183434359

 0

 0

 .000561612797025

 0

 0

 0

 0

 0

 0

 0

 0

 0

 0

 0

 0

 0

 0

 0

 .000561612797025

 0

 .000561612797025

 .00133625113775

 .00133625113775

 .00040668512888

 0

 .00040668512888

 0

 0

 0

 0

 0

 .00092956600887

 .00092956600887

 0

 0

 0

 0

 0

 0

 0

 0

 0

 0

 0

 0

 0

 0

 0

 0

 0

 0

 0

 0

 0

 0

 0

 0

 0

 0

 0

 0

 0

 0

 0

 0

 0

 0

 0

 0

 0

 0

 0

 0

 0

 0

 0

 0

 0

 0

 0

 0

 0

 0

 0

 0

 0

 0

 0

 0

 0

 0

 0

 0

 0

 0

 0

 0

 0

 .000755272382207

 .000755272382207

 0

 0

 0

 .000755272382207

 .000755272382207

 .000755272382207

 0

 0

 0

 0

 0

 0

 0

 0

 0

 0

 0

 0

 0

 0

 0

 0

 0

 0

 0

 0

 0

 0

 0

 0

 0

 0

 .000174293626663

 0

 0

 0

 0

 0

 0

 0

 0

 0

 0

 0

 0

 0

 0

 0

 0

 0

 0

 .000174293626663

 0

 0

 0

 0

 .000174293626663

 .000174293626663

 .000174293626663

 0

 0

 0

 0

 0

 0

 0

 0

 0

 0

 0

 0

 0

 0

 0

 .968472219532311

 .964812053372389

 2.517574607359E-04

 2.517574607359E-04

 0

 .000116195751109

 5.80978755544E-05

 0

 0

 7.74638340725E-05

 0

 0

 0

 0

 0

 0

 0

 0

 0

 0

 0

 0

 0

 0

 0

 0

 0

 0

 0

 .000116195751109

 .000116195751109

 0

 0

 0

 0

 0

 .000116195751109

 0

 0

 0

 0

 0

 0

 0

 0

 0

 0

 0

 0

 0

 0

 0

 0

 0

 0

 0

 0

 0

 0

 0

 0

 0

 0

 0

 0

 0

 0

 0

 .000154927668145

 .000154927668145

 0

 .000154927668145

 1.7042043495941E-03

 0

 0

 0

 0

 0

 0

 0

 0

 0

 0

 0

 0

 0

 0

 0

 0

 0

 0

 0

 0

 0

 0

 0

 0

 0

 0

 0

 0

 0

 0

 0

 0

 0

 0

 0

 0

 0

 0

 0

 0

 0

 0

 0

 0

 0

 0

 0

 0

 0

 0

 0

 .00092956600887

 0

 .00092956600887

 0

 0

 0

 7.746383407241E-04

 .000367953211844

 .000154927668145

 0

 1.93659585181E-05

 0

 0

 0

 0

 0

 .000232391502217

 0

 0

 0

 0

 .000271123419254

 .000271123419254

 0

 0

 0

 .000116195751109

 .000154927668145

 0

 0

 0

 0

 0

 0

 0

 0

 0

 0

 0

 0

 0

 .960222321203591

 0

 0

 .960125491411

 .960125491411

 9.68297925906E-05

 0

 9.68297925906E-05

 0

 2.0915235199601E-03

 2.0915235199601E-03

 1.93659585181E-05

 0

 0

 0

 0

 0

 .000832736216279

 0

 0

 0

 .0010651277185

 .000174293626663

 0

 0

 0

 0

 0

 0

 0

 0

 0

 0

 0

 0

 .000213025543699

 0

 0

 0

 0

 0

 0

 0

 0

 0

 0

 0

 0

 0

 .000213025543699

 0

 0

 0

 0

 0

 0

 0

 0

 0

 0

 0

 0

 0

 0

 0

 0

 0

 .000213025543699

 0

 0

 .000213025543699

 0

 0

 0

 0

 0

 0

 0

 0

 0

 0

 0

 0

 0

 3.4471406162237E-03

 0

 0

 0

 0

 0

 0

 0

 0

 0

 0

 0

 8.327362162783E-04

 0

 0

 4.066851288802E-04

 .000367953211844

 0

 0

 0

 3.87319170362E-05

 0

 0

 0

 0

 0

 0

 0

 0

 0

 0

 0

 0

 0

 0

 0

 0

 0

 0

 0

 0

 0

 0

 0

 0

 0

 0

 0

 0

 0

 0

 0

 0

 0

 0

 0

 0

 0

 0

 0

 0

 0

 0

 .00040668512888

 .00040668512888

 0

 0

 0

 0

 1.93659585181E-05

 0

 0

 0

 1.93659585181E-05

 0

 0

 0

 0

 0

 0

 0

 0

 0

 0

 0

 0

 0

 0

 0

 1.0070298429414E-03

 1.0070298429414E-03

 .00040668512888

 0

 0

 0

 .000232391502217

 0

 .00030985533629

 0

 0

 0

 0

 0

 0

 0

 0

 0

 0

 0

 5.80978755544E-05

 0

 0

 0

 0

 0

 0

 0

 0

 0

 0

 0

 0

 0

 0

 0

 0

 0

 0

 0

 0

 0

 0

 .000426051087399

 .000426051087399

 .000426051087399

 2.517574607358E-04

 1.549276681452E-04

 .000116195751109

 0

 3.87319170362E-05

 0

 0

 0

 9.68297925906E-05

 9.68297925906E-05

 0

 0

 0

 0

 0

 0

 0

 0

 0

 0

 0

 0

 0

 0

 0

 0

 0

 0

 0

 9.295660088692E-04

 3.87319170362E-05

 0

 0

 0

 0

 0

 0

 3.87319170362E-05

 .000890834091833

 0

 .000658442589616

 0

 .000232391502217

 0

 0

 0

 0

 0

 0

 0

 0

 0

 0

 0

 0

 0

 0

 0

 0

 0

 0

 0

 .000774638340725

 .000774638340725

 0

 0

 0

 0

 0

 0

 0

 0

 0

 0

 0

 .000445417045917

 .000445417045917

 0

 0

 .000445417045917

 0

 0

 .000329221294808

 .000329221294808

 .000329221294808

 0

 0

 0

 0

 0

 0

 0

 0

 0

 0

 0

 0

 0

 0

 0

 0

 0

 0

 0

 0

 0

 0

 0

 0

 0

 0

 0
