# Supplementary material for: First Insight into Microbiome Profiles of Myrmecophilous Beetles and Their Host, Red Wood Ant Formica polyctena (Hymenoptera: Formicidae)—A Case Study
Source: Insects. 2020 Feb 19;11(2):134. doi: 10.3390/insects11020134 (PMC7073670; doi:10.3390/insects11020134)
Supplement: Supplementary file 1 [file insects-11-00134-s001.zip › Supplementary Files/S2_Figure.zip!/Supplemental_Figure_S2/Supplemental_Figure_S2_TA1.html]

Javascript must be enabled to view this page.

magnitude

 1.00000000000007

 1.00000000000007

 0

 0

 0

 0

 0

 0

 .000727008360595

 .000727008360595

 .000727008360595

 .000727008360595

 0

 0

 0

 0

 .000121168060099

 .000121168060099

 0

 0

 .000484672240397

 0

 0

 0

 0

 0

 0

 0

 0

 0

 0

 0

 0

 0

 0

 0

 0

 0

 0

 0

 0

 0

 0

 0

 0

 0

 0

 0

 0

 0

 0

 0

 0

 0

 0

 0

 0

 0

 0

 0

 0

 0

 0

 0

 0

 0

 0

 0

 0

 0

 0

 0

 0

 0

 0

 0

 0

 0

 0

 0

 0

 0

 0

 0

 0

 0

 0

 0

 0

 0

 0

 0

 0

 .027868653822809

 0

 0

 0

 0

 .000121168060099

 0

 0

 0

 0

 .000121168060099

 .000121168060099

 .000121168060099

 0

 0

 0

 0

 0

 0

 0

 0

 0

 0

 0

 0

 0

 0

 0

 0

 0

 0

 0

 0

 0

 0

 0

 0

 0

 0

 .027626317702611

 0

 0

 0

 0

 0

 0

 0

 0

 0

 0

 0

 0

 0

 .016600024233569

 .001575184781288

 0

 0

 .00121168060099

 .000121168060099

 .000242336120199

 0

 0

 0

 0

 .0112686295892

 .0112686295892

 .002908033442385

 0

 .00181752090149

 .000242336120199

 .000848176420696

 0

 0

 0

 0

 0

 .000848176420696

 .000848176420696

 0

 0

 .001575184781292

 0

 0

 .000605840300497

 .000605840300497

 0

 0

 0

 .000969344480795

 0

 .000969344480795

 0

 0

 0

 0

 0

 0

 0

 0

 0

 0

 0

 0

 0

 0

 0

 0

 0

 0

 0

 0

 0

 .000727008360596

 0

 0

 0

 0

 0

 0

 0

 0

 .000121168060099

 0

 0

 0

 0

 .000121168060099

 0

 0

 0

 0

 0

 0

 0

 0

 0

 0

 0

 0

 0

 0

 0

 .000121168060099

 .000121168060099

 0

 0

 0

 0

 0

 0

 0

 0

 0

 0

 0

 0

 0

 0

 0

 0

 0

 0

 0

 0

 0

 0

 .000484672240398

 0

 .000242336120199

 0

 .000242336120199

 0

 0

 0

 0

 0

 0

 0

 0

 0

 0

 0

 0

 0

 0

 0

 0

 .006179571065066

 .002181025081786

 0

 .00145401672119

 0

 .000242336120199

 .000484672240397

 .00399854598328

 .00399854598328

 0

 0

 0

 0

 .000242336120198

 .000242336120198

 0

 0

 0

 0

 .000121168060099

 .000121168060099

 0

 .00230219314189

 .00230219314189

 0

 0

 .00230219314189

 0

 0

 0

 0

 0

 0

 0

 0

 0

 0

 0

 0

 0

 0

 0

 .000121168060099

 0

 0

 0

 0

 0

 0

 0

 0

 .000121168060099

 0

 0

 0

 0

 0

 .000121168060099

 0

 .000121168060099

 0

 0

 0

 0

 0

 0

 0

 0

 0

 0

 0

 0

 0

 0

 0

 0

 0

 0

 0

 0

 .000363504180298

 .000363504180298

 .000242336120199

 0

 0

 0

 0

 .000242336120199

 0

 .000242336120199

 0

 0

 0

 .000121168060099

 0

 0

 .000121168060099

 0

 0

 0

 0

 0

 0

 0

 0

 0

 0

 0

 0

 0

 .000121168060099

 0

 0

 0

 0

 0

 0

 0

 0

 0

 0

 0

 0

 0

 0

 0

 0

 0

 0

 0

 0

 0

 0

 0

 0

 0

 0

 0

 0

 0

 0

 0

 0

 0

 0

 0

 0

 0

 0

 0

 0

 0

 0

 0

 0

 0

 0

 0

 0

 0

 0

 0

 0

 0

 0

 0

 0

 0

 0

 0

 0

 0

 0

 0

 0

 0

 0

 0

 0

 0

 0

 0

 0

 0

 0

 0

 0

 0

 0

 0

 0

 0

 0

 0

 0

 0

 0

 0

 0

 0

 0

 0

 0

 0

 0

 0

 0

 0

 0

 0

 0

 0

 0

 0

 0

 0

 0

 0

 0

 0

 0

 0

 0

 0

 0

 0

 0

 0

 0

 0

 0

 0

 0

 0

 0

 0

 0

 0

 0

 0

 .000121168060099

 .000121168060099

 .000121168060099

 0

 0

 .000121168060099

 .000121168060099

 0

 0

 0

 0

 0

 0

 0

 0

 0

 0

 0

 0

 0

 0

 0

 0

 0

 0

 0

 0

 0

 0

 0

 0

 .001938688961589

 .001938688961589

 .001211680600993

 0

 0

 0

 0

 0

 0

 0

 0

 0

 0

 0

 0

 0

 0

 0

 0

 0

 .000484672240397

 0

 0

 0

 .000484672240397

 0

 0

 0

 0

 0

 0

 0

 0

 0

 0

 .000727008360596

 0

 0

 .000727008360596

 0

 0

 .000727008360596

 0

 0

 0

 0

 0

 0

 0

 0

 0

 0

 0

 .000121168060099

 .000121168060099

 0

 0

 .000605840300497

 0

 .000605840300497

 0

 0

 0

 0

 0

 0

 0

 0

 0

 0

 0

 0

 0

 0

 0

 0

 0

 0

 0

 0

 0

 0

 0

 0

 0

 0

 0

 0

 0

 0

 0

 0

 0

 0

 0

 0

 0

 0

 0

 0

 0

 0

 0

 0

 0

 0

 0

 0

 0

 0

 0

 0

 0

 0

 0

 0

 0

 0

 0

 0

 0

 0

 0

 0

 0

 0

 0

 0

 0

 0

 0

 0

 0

 0

 0

 0

 0

 .000242336120199

 0

 0

 0

 0

 0

 0

 0

 0

 0

 0

 0

 0

 .000242336120199

 .000242336120199

 0

 0

 0

 0

 0

 .000242336120199

 .000242336120199

 0

 0

 0

 0

 0

 0

 0

 0

 0

 0

 0

 0

 .001211680600994

 0

 0

 0

 0

 0

 0

 0

 0

 0

 0

 0

 0

 0

 0

 0

 0

 0

 0

 .001211680600994

 .000363504180298

 .000363504180298

 .000363504180298

 0

 .000848176420696

 .000848176420696

 0

 0

 0

 0

 .000605840300497

 .000242336120199

 0

 0

 0

 0

 0

 0

 0

 0

 0

 0

 .967163455713186

 .924875802738464

 .001090512540894

 .001090512540894

 0

 0

 0

 0

 .000242336120199

 0

 0

 0

 0

 .000727008360596

 .000121168060099

 0

 0

 0

 0

 0

 0

 0

 0

 0

 0

 0

 0

 0

 0

 .000121168060099

 .000121168060099

 0

 0

 0

 0

 0

 .000121168060099

 0

 0

 0

 0

 0

 0

 0

 .000605840300497

 .000605840300497

 0

 .000605840300497

 0

 0

 0

 0

 0

 0

 0

 0

 .000121168060099

 .000121168060099

 0

 .000121168060099

 0

 0

 0

 0

 0

 0

 0

 0

 0

 0

 0

 0

 .001211680600993

 0

 0

 0

 0

 .000484672240397

 .000121168060099

 0

 0

 0

 .000363504180298

 0

 0

 0

 0

 0

 0

 0

 0

 0

 0

 0

 0

 0

 0

 0

 0

 0

 0

 0

 0

 .000242336120199

 .000242336120199

 0

 0

 .000121168060099

 .000121168060099

 .000242336120199

 0

 .000242336120199

 0

 0

 0

 0

 0

 0

 0

 0

 0

 0

 0

 0

 0

 0

 0

 0

 0

 0

 .000121168060099

 0

 .000121168060099

 0

 0

 0

 0

 0

 0

 0

 0

 0

 0

 0

 0

 .000363504180297

 .000363504180297

 0

 0

 0

 .000121168060099

 .000121168060099

 0

 0

 0

 .000121168060099

 0

 .000121168060099

 0

 0

 0

 .000121168060099

 .000121168060099

 0

 0

 .919786744214298

 0

 0

 .000242336120199

 .000242336120199

 .919544408094099

 .000121168060099

 .919423240034

 0

 .001454016721188

 .001454016721188

 0

 0

 0

 0

 0

 .000121168060099

 0

 0

 .000121168060099

 0

 .00121168060099

 0

 0

 0

 0

 0

 0

 0

 0

 0

 0

 0

 0

 0

 0

 0

 0

 0

 0

 0

 0

 0

 0

 0

 0

 0

 0

 0

 0

 0

 0

 0

 0

 0

 0

 0

 0

 0

 0

 0

 0

 0

 0

 0

 0

 0

 0

 0

 0

 0

 0

 0

 0

 0

 0

 0

 0

 0

 0

 0

 0

 0

 0

 .042287652974722

 0

 0

 0

 0

 0

 0

 0

 0

 0

 0

 0

 .004362050163572

 0

 0

 .003877377923175

 .000242336120199

 0

 0

 0

 .000363504180298

 0

 .00290803344238

 0

 0

 0

 0

 0

 0

 0

 0

 0

 0

 .000363504180298

 0

 0

 0

 0

 0

 0

 0

 0

 0

 0

 0

 0

 0

 0

 0

 0

 0

 0

 0

 0

 0

 0

 0

 0

 0

 0

 .000484672240397

 0

 .000484672240397

 0

 0

 0

 0

 0

 0

 0

 0

 0

 0

 0

 0

 0

 0

 0

 0

 0

 0

 0

 0

 0

 0

 0

 0

 0

 0

 .037198594450555

 .037198594450555

 .00157518478129

 0

 0

 0

 .0278686538229

 .000363504180298

 0

 0

 .000484672240397

 0

 0

 0

 0

 0

 0

 .00436205016358

 0

 0

 .00254452926209

 0

 0

 0

 0

 0

 0

 0

 0

 0

 0

 0

 0

 0

 0

 0

 0

 0

 0

 0

 0

 0

 0

 0

 0

 0

 0

 0

 0

 0

 0

 0

 0

 0

 0

 0

 0

 0

 0

 .000121168060099

 .000121168060099

 .000121168060099

 0

 0

 0

 0

 0

 0

 0

 0

 0

 0

 0

 0

 0

 .000605840300496

 .000605840300496

 0

 0

 0

 0

 .000484672240397

 0

 .000121168060099

 0

 0

 0

 0

 0

 0

 0

 0

 0

 0

 0

 0

 0

 0

 0

 0

 0

 0

 0

 .000121168060099

 .000121168060099

 .000121168060099

 .000121168060099

 .000121168060099

 .000242336120199

 .000242336120199

 0

 0

 0

 0

 0

 0

 0

 0

 0

 0

 0

 .000242336120199

 .000242336120199

 0

 0

 .000242336120199

 0

 0

 0

 0

 0

 0

 0

 0

 0

 0

 0

 0

 0

 0

 0

 0

 0

 0

 0

 0

 0

 0

 0

 0

 0

 0

 0

 0

 0

 0

 0

 0
