# Supplementary material for: First Insight into Microbiome Profiles of Myrmecophilous Beetles and Their Host, Red Wood Ant Formica polyctena (Hymenoptera: Formicidae)—A Case Study
Source: Insects. 2020 Feb 19;11(2):134. doi: 10.3390/insects11020134 (PMC7073670; doi:10.3390/insects11020134)
Supplement: Supplementary file 1 [file insects-11-00134-s001.zip › Supplementary Files/S2_Figure.zip!/Supplemental_Figure_S2/Supplemental_Figure_S2_TA2.html]

Javascript must be enabled to view this page.

magnitude

 .999999999999856

 .999999999999856

 0

 0

 0

 0

 0

 0

 9.22608521827E-05

 9.22608521827E-05

 9.22608521827E-05

 9.22608521827E-05

 0

 0

 4.61304260914E-05

 0

 0

 1.53768086971E-05

 0

 3.07536173942E-05

 0

 0

 0

 0

 0

 0

 0

 0

 0

 0

 0

 0

 0

 0

 0

 0

 0

 0

 0

 0

 0

 0

 0

 0

 0

 0

 0

 0

 0

 0

 0

 0

 0

 0

 0

 0

 0

 0

 0

 0

 0

 0

 0

 0

 0

 0

 0

 0

 0

 0

 0

 0

 0

 0

 0

 0

 0

 0

 0

 0

 0

 0

 0

 0

 0

 0

 0

 0

 0

 0

 0

 0

 0

 6.4275060353992E-03

 0

 0

 0

 0

 0

 0

 0

 0

 0

 0

 0

 0

 0

 0

 0

 0

 0

 0

 0

 0

 0

 0

 0

 0

 0

 0

 0

 0

 0

 0

 0

 0

 0

 0

 0

 0

 0

 0

 6.3506219919136E-03

 0

 0

 0

 0

 0

 0

 0

 0

 0

 0

 3.07536173942E-05

 3.07536173942E-05

 3.07536173942E-05

 3.9825934525581E-03

 5.228114957022E-04

 0

 0

 .000353666600034

 .000138391278274

 3.07536173942E-05

 0

 0

 0

 0

 .00258330386112

 .00258330386112

 5.535651130959E-04

 0

 3.07536173942E-05

 1.53768086971E-05

 .000430550643519

 0

 0

 7.68840434856E-05

 0

 0

 .00032291298264

 .00032291298264

 0

 0

 9.22608521827E-05

 0

 0

 0

 0

 0

 0

 0

 9.22608521827E-05

 0

 9.22608521827E-05

 0

 0

 0

 0

 0

 0

 0

 0

 0

 0

 0

 0

 0

 0

 0

 0

 0

 0

 0

 0

 0

 2.306521304572E-04

 0

 0

 0

 0

 0

 0

 0

 0

 1.53768086971E-05

 0

 0

 0

 0

 1.53768086971E-05

 0

 0

 0

 0

 0

 0

 0

 0

 0

 0

 0

 0

 0

 0

 0

 1.53768086971E-05

 1.53768086971E-05

 0

 0

 0

 0

 0

 0

 0

 0

 0

 0

 0

 0

 0

 0

 0

 0

 0

 0

 0

 0

 0

 0

 .000199898513063

 0

 0

 0

 .000199898513063

 0

 0

 0

 0

 0

 0

 0

 0

 0

 0

 0

 0

 0

 0

 0

 0

 1.3992895914369E-03

 1.230144695769E-04

 0

 9.22608521827E-05

 0

 1.53768086971E-05

 1.53768086971E-05

 .00127627512186

 .00127627512186

 0

 0

 0

 0

 2.306521304565E-04

 2.306521304565E-04

 0

 4.61304260914E-05

 0

 0

 1.53768086971E-05

 .000169144895668

 0

 .000384420217428

 .000384420217428

 0

 0

 .000384420217428

 0

 0

 0

 0

 0

 0

 0

 0

 0

 0

 0

 0

 0

 0

 0

 7.68840434856E-05

 0

 0

 0

 0

 0

 0

 0

 0

 7.68840434856E-05

 0

 0

 0

 0

 0

 7.68840434856E-05

 0

 0

 1.53768086971E-05

 0

 6.15072347885E-05

 0

 0

 0

 0

 0

 0

 0

 0

 0

 0

 0

 0

 0

 0

 0

 0

 0

 2.306521304568E-04

 2.306521304568E-04

 0

 0

 0

 0

 0

 0

 0

 0

 0

 0

 0

 4.61304260913E-05

 0

 0

 4.61304260913E-05

 0

 1.53768086971E-05

 0

 0

 0

 0

 0

 0

 0

 0

 0

 0

 0

 3.07536173942E-05

 0

 0

 0

 0

 0

 0

 0

 0

 0

 0

 0

 0

 0

 4.61304260914E-05

 0

 0

 0

 0

 0

 0

 0

 4.61304260914E-05

 0

 4.61304260914E-05

 0

 0

 1.383912782741E-04

 0

 0

 0

 0

 1.383912782741E-04

 0

 9.22608521827E-05

 4.61304260914E-05

 0

 0

 0

 0

 0

 0

 0

 0

 0

 0

 0

 0

 0

 0

 0

 0

 0

 0

 0

 0

 0

 0

 0

 0

 0

 0

 0

 0

 0

 0

 0

 0

 0

 0

 0

 0

 0

 0

 0

 0

 0

 0

 0

 0

 0

 0

 0

 0

 0

 0

 0

 0

 0

 0

 0

 0

 0

 0

 0

 0

 0

 0

 0

 0

 0

 0

 0

 0

 0

 0

 0

 0

 0

 0

 0

 0

 0

 0

 0

 0

 0

 0

 0

 0

 0

 0

 0

 0

 0

 0

 0

 0

 0

 0

 0

 0

 0

 0

 0

 0

 0

 0

 0

 0

 0

 0

 0

 0

 0

 0

 0

 0

 0

 0

 0

 0

 0

 0

 0

 0

 0

 0

 0

 0

 0

 2.306521304568E-04

 1.998985130626E-04

 6.15072347885E-05

 0

 0

 0

 0

 0

 0

 0

 0

 0

 0

 0

 0

 0

 0

 0

 0

 0

 0

 0

 0

 0

 0

 0

 0

 0

 0

 0

 0

 0

 0

 0

 0

 6.15072347885E-05

 0

 0

 6.15072347885E-05

 0

 0

 1.383912782741E-04

 0

 0

 4.61304260914E-05

 4.61304260914E-05

 0

 0

 0

 0

 0

 0

 0

 0

 0

 0

 0

 9.22608521827E-05

 0

 9.22608521827E-05

 3.07536173942E-05

 3.07536173942E-05

 0

 0

 0

 0

 0

 0

 0

 0

 3.07536173942E-05

 0

 0

 0

 0

 3.07536173942E-05

 0

 0

 0

 0

 0

 0

 0

 0

 0

 0

 0

 0

 0

 0

 0

 0

 0

 0

 0

 0

 0

 0

 0

 0

 0

 0

 0

 0

 0

 0

 0

 0

 0

 0

 0

 0

 0

 0

 0

 0

 0

 0

 0

 0

 0

 0

 0

 0

 0

 0

 0

 0

 0

 0

 0

 0

 0

 0

 0

 0

 0

 1.53768086971E-05

 0

 0

 0

 0

 0

 0

 0

 0

 0

 0

 0

 0

 1.53768086971E-05

 1.53768086971E-05

 0

 0

 0

 0

 0

 0

 0

 0

 0

 0

 0

 0

 0

 1.53768086971E-05

 1.53768086971E-05

 0

 0

 0

 0

 0

 0

 0

 0

 0

 0

 0

 0

 0

 0

 0

 0

 0

 0

 0

 0

 0

 0

 0

 0

 0

 0

 0

 0

 0

 0

 0

 0

 0

 0

 0

 0

 0

 0

 0

 0

 0

 0

 0

 0

 0

 0

 .992957421616572

 .990820045207673

 0

 0

 0

 0

 0

 0

 0

 0

 0

 0

 0

 0

 0

 0

 0

 0

 0

 0

 0

 0

 0

 0

 0

 0

 0

 0

 0

 .000123014469577

 .000123014469577

 4.61304260914E-05

 0

 6.15072347885E-05

 0

 0

 1.53768086971E-05

 0

 0

 0

 0

 0

 0

 0

 0

 0

 0

 0

 0

 0

 0

 0

 0

 0

 0

 0

 0

 0

 0

 0

 0

 0

 0

 0

 0

 1.53768086971E-05

 1.53768086971E-05

 1.53768086971E-05

 0

 0

 0

 0

 1.845217043655E-04

 0

 0

 0

 0

 1.076376608799E-04

 4.61304260914E-05

 0

 0

 1.53768086971E-05

 4.61304260914E-05

 0

 0

 0

 0

 0

 0

 0

 0

 4.61304260914E-05

 4.61304260914E-05

 0

 0

 0

 0

 0

 0

 0

 0

 0

 0

 0

 0

 0

 0

 0

 0

 1.53768086971E-05

 0

 1.53768086971E-05

 0

 0

 0

 0

 0

 0

 0

 0

 0

 0

 0

 0

 0

 0

 0

 0

 0

 0

 1.53768086971E-05

 1.53768086971E-05

 0

 0

 0

 0

 0

 0

 0

 0

 0

 0

 0

 0

 0

 1.53768086971E-05

 1.53768086971E-05

 0

 0

 0

 1.53768086971E-05

 0

 0

 0

 0

 0

 0

 0

 0

 0

 0

 0

 0

 0

 0

 .990281856903274

 0

 0

 1.53768086971E-05

 1.53768086971E-05

 .990266480094577

 .000123014469577

 .990143465625

 0

 1.998985130626E-04

 1.998985130626E-04

 0

 0

 4.61304260914E-05

 0

 0

 0

 3.07536173942E-05

 0

 0

 0

 .000123014469577

 0

 0

 0

 0

 0

 0

 0

 0

 0

 0

 0

 0

 0

 0

 0

 0

 0

 0

 0

 0

 0

 0

 0

 0

 0

 0

 0

 0

 0

 0

 0

 0

 0

 0

 0

 0

 0

 0

 0

 0

 0

 0

 0

 0

 0

 0

 0

 0

 0

 0

 0

 0

 0

 0

 0

 0

 0

 0

 0

 0

 0

 0

 2.1373764088985E-03

 0

 0

 0

 0

 0

 0

 0

 0

 0

 0

 0

 1.4454200175291E-03

 0

 0

 .001307028739255

 9.22608521827E-05

 0

 .000384420217428

 0

 0

 0

 .000553565113096

 0

 0

 1.53768086971E-05

 0

 3.07536173942E-05

 0

 0

 0

 0

 0

 .000230652130457

 0

 0

 0

 0

 0

 0

 0

 0

 0

 0

 0

 0

 0

 0

 0

 0

 0

 0

 0

 0

 0

 0

 0

 0

 0

 0

 0

 0

 0

 0

 0

 0

 0

 0

 0

 1.383912782741E-04

 7.68840434856E-05

 4.61304260914E-05

 0

 1.53768086971E-05

 0

 0

 0

 0

 0

 0

 0

 0

 0

 0

 0

 0

 0

 0

 0

 3.690434087301E-04

 3.690434087301E-04

 .000184521704365

 0

 0

 0

 .000169144895668

 0

 0

 0

 0

 0

 0

 0

 0

 0

 0

 1.53768086971E-05

 0

 0

 0

 0

 0

 0

 0

 0

 0

 0

 0

 0

 0

 0

 0

 0

 0

 0

 0

 0

 0

 0

 0

 0

 0

 1.53768086971E-05

 1.53768086971E-05

 1.53768086971E-05

 2.460289391537E-04

 .000153768086971

 .000153768086971

 0

 0

 0

 0

 0

 9.22608521827E-05

 9.22608521827E-05

 0

 0

 0

 0

 0

 0

 0

 0

 0

 0

 0

 0

 0

 0

 0

 0

 0

 0

 0

 6.15072347885E-05

 6.15072347885E-05

 0

 0

 0

 0

 6.15072347885E-05

 0

 0

 0

 0

 0

 0

 0

 0

 0

 0

 0

 0

 0

 0

 0

 0

 0

 0

 0

 0

 0

 0

 0

 0

 0

 0

 4.61304260913E-05

 4.61304260913E-05

 1.53768086971E-05

 0

 0

 0

 0

 0

 1.53768086971E-05

 1.53768086971E-05

 0

 0

 0

 0

 0

 0

 0

 0

 0

 0

 0

 0

 0

 0

 0

 0

 0

 0

 0

 3.07536173942E-05

 0

 0

 0

 0

 0

 0

 3.07536173942E-05

 0

 0

 0

 3.07536173942E-05

 0

 0

 0

 0

 0

 0

 0

 0

 0
