# Supplementary material for: First Insight into Microbiome Profiles of Myrmecophilous Beetles and Their Host, Red Wood Ant Formica polyctena (Hymenoptera: Formicidae)—A Case Study
Source: Insects. 2020 Feb 19;11(2):134. doi: 10.3390/insects11020134 (PMC7073670; doi:10.3390/insects11020134)
Supplement: Supplementary file 1 [file insects-11-00134-s001.zip › Supplementary Files/S2_Figure.zip!/Supplemental_Figure_S2/Supplemental_Figure_S2_TA6.html]

Javascript must be enabled to view this page.

magnitude

 .99999999999908

 .99999999999908

 0

 0

 0

 0

 0

 0

 7.671770951614E-04

 7.671770951614E-04

 5.523675085164E-04

 5.523675085164E-04

 3.06870838064E-05

 0

 .000122748335226

 0

 0

 .000184122502839

 0

 0

 .000214809586645

 0

 0

 0

 0

 0

 0

 .000214809586645

 .000214809586645

 .000214809586645

 0

 0

 0

 0

 0

 0

 0

 0

 0

 0

 0

 0

 0

 0

 0

 0

 0

 0

 0

 0

 0

 0

 0

 0

 0

 0

 0

 0

 0

 0

 0

 0

 0

 0

 0

 0

 0

 0

 0

 0

 0

 0

 0

 0

 0

 0

 0

 0

 0

 0

 0

 0

 0

 0

 0

 0

 0

 0

 0

 0

 0

 0

 0

 .01215208518734

 0

 0

 0

 0

 0

 0

 0

 0

 0

 0

 0

 0

 0

 0

 0

 0

 0

 0

 0

 0

 0

 0

 0

 0

 0

 0

 0

 0

 0

 0

 0

 0

 0

 0

 0

 0

 0

 0

 1.20907110197272E-02

 0

 0

 0

 0

 0

 0

 0

 0

 0

 0

 9.20612514193E-05

 9.20612514193E-05

 9.20612514193E-05

 4.4496271519311E-03

 4.909933409033E-04

 3.06870838064E-05

 0

 .000122748335226

 .000276183754258

 6.13741676129E-05

 0

 0

 0

 0

 .00282321171019

 .00282321171019

 6.137416761288E-04

 3.06870838064E-05

 .000184122502839

 0

 .000368245005677

 0

 0

 3.06870838064E-05

 0

 0

 .000521680424709

 .000521680424709

 0

 0

 .000460306257097

 0

 0

 .000276183754258

 .000276183754258

 0

 0

 0

 .000184122502839

 0

 .000184122502839

 0

 0

 0

 0

 0

 0

 0

 0

 0

 0

 0

 0

 0

 0

 0

 0

 0

 0

 0

 0

 0

 1.3195446036714E-03

 0

 0

 0

 0

 0

 0

 3.06870838064E-05

 3.06870838064E-05

 .000214809586645

 0

 0

 0

 0

 .000214809586645

 0

 0

 0

 0

 0

 0

 0

 0

 0

 0

 0

 0

 0

 0

 0

 0

 0

 0

 0

 0

 0

 0

 0

 0

 0

 0

 0

 0

 0

 0

 0

 0

 0

 0

 0

 0

 0

 0

 0

 .00107404793322

 0

 0

 0

 .00107404793322

 0

 0

 0

 0

 0

 0

 0

 0

 0

 0

 0

 0

 0

 0

 0

 0

 3.1914567158674E-03

 7.978641789674E-04

 0

 .000583054592322

 0

 .000184122502839

 3.06870838064E-05

 .0023935925369

 .0023935925369

 0

 0

 0

 0

 .000337557921871

 .000337557921871

 0

 .000122748335226

 0

 0

 0

 .000214809586645

 0

 .00224015711787

 .00224015711787

 0

 0

 .00224015711787

 0

 0

 0

 0

 0

 0

 0

 0

 0

 0

 0

 0

 0

 0

 0

 6.13741676128E-05

 0

 0

 0

 0

 0

 0

 0

 0

 6.13741676128E-05

 0

 0

 3.06870838064E-05

 3.06870838064E-05

 0

 3.06870838064E-05

 0

 3.06870838064E-05

 0

 0

 0

 0

 0

 0

 0

 0

 0

 0

 0

 0

 0

 0

 0

 0

 0

 0

 0

 0

 3.682450056771E-04

 3.682450056771E-04

 2.454966704514E-04

 0

 0

 3.06870838064E-05

 3.06870838064E-05

 .000214809586645

 .000214809586645

 0

 0

 0

 0

 3.06870838064E-05

 0

 0

 3.06870838064E-05

 0

 3.06870838064E-05

 0

 0

 0

 0

 0

 0

 0

 0

 0

 0

 0

 0

 0

 0

 0

 0

 0

 0

 0

 0

 0

 0

 0

 0

 0

 9.20612514193E-05

 0

 0

 0

 0

 0

 0

 0

 9.20612514193E-05

 6.13741676129E-05

 3.06870838064E-05

 0

 0

 0

 0

 0

 0

 0

 0

 0

 0

 0

 0

 0

 0

 0

 0

 0

 0

 0

 0

 0

 0

 0

 0

 0

 0

 0

 1.227483352257E-04

 3.06870838064E-05

 3.06870838064E-05

 3.06870838064E-05

 3.06870838064E-05

 0

 0

 0

 0

 0

 0

 0

 0

 0

 0

 0

 0

 0

 0

 9.20612514193E-05

 0

 0

 0

 0

 0

 9.20612514193E-05

 9.20612514193E-05

 0

 9.20612514193E-05

 0

 0

 0

 0

 0

 0

 0

 0

 0

 0

 0

 0

 0

 0

 0

 0

 0

 0

 0

 0

 0

 0

 0

 0

 0

 0

 0

 0

 0

 0

 0

 0

 0

 0

 0

 0

 0

 0

 0

 0

 0

 0

 0

 0

 0

 0

 0

 0

 0

 0

 0

 0

 0

 0

 0

 0

 0

 0

 0

 0

 0

 0

 0

 0

 0

 0

 0

 0

 0

 0

 0

 0

 0

 0

 0

 0

 0

 0

 0

 0

 6.137416761283E-04

 6.137416761283E-04

 .000276183754258

 0

 0

 0

 0

 0

 0

 0

 0

 0

 0

 0

 0

 0

 0

 0

 0

 0

 0

 0

 0

 0

 0

 0

 0

 0

 0

 0

 0

 0

 0

 0

 0

 .000276183754258

 0

 0

 .000276183754258

 0

 0

 3.375579218703E-04

 0

 0

 0

 0

 0

 0

 0

 0

 0

 0

 0

 0

 0

 0

 0

 3.375579218703E-04

 9.20612514193E-05

 .000245496670451

 0

 0

 0

 0

 0

 0

 0

 0

 0

 0

 0

 0

 0

 0

 0

 0

 0

 0

 0

 0

 0

 0

 0

 0

 0

 0

 0

 0

 0

 0

 0

 0

 0

 0

 0

 0

 0

 0

 0

 0

 0

 0

 0

 0

 0

 0

 0

 0

 0

 0

 0

 0

 0

 0

 0

 0

 0

 0

 0

 0

 0

 0

 0

 0

 0

 0

 0

 0

 0

 0

 0

 0

 0

 0

 0

 0

 0

 0

 0

 0

 0

 0

 0

 0

 0

 0

 0

 0

 0

 0

 0

 0

 0

 0

 0

 0

 0

 0

 0

 0

 0

 0

 0

 0

 0

 0

 0

 0

 0

 0

 0

 0

 0

 0

 0

 0

 0

 0

 0

 0

 0

 0

 0

 0

 0

 0

 0

 0

 0

 0

 0

 0

 0

 0

 0

 0

 0

 0

 0

 0

 0

 0

 0

 0

 0

 0

 0

 0

 0

 0

 0

 0

 0

 .401018811181741

 .39791941571729

 1.534354190324E-04

 1.534354190324E-04

 0

 0

 0

 0

 0

 0

 3.06870838064E-05

 0

 0

 0

 0

 0

 0

 0

 0

 0

 0

 .000122748335226

 0

 0

 0

 0

 0

 0

 0

 9.20612514193E-05

 9.20612514193E-05

 0

 0

 0

 0

 0

 9.20612514193E-05

 0

 0

 0

 0

 0

 0

 0

 3.06870838064E-05

 3.06870838064E-05

 0

 0

 0

 0

 3.06870838064E-05

 0

 0

 0

 0

 0

 0

 0

 0

 0

 0

 0

 0

 0

 0

 0

 0

 0

 3.06870838064E-05

 3.06870838064E-05

 3.06870838064E-05

 0

 1.534354190322E-04

 0

 0

 0

 0

 6.13741676129E-05

 0

 0

 0

 0

 0

 0

 0

 0

 0

 0

 6.13741676129E-05

 0

 0

 0

 0

 0

 0

 0

 0

 0

 0

 0

 0

 0

 0

 0

 0

 0

 0

 0

 0

 0

 0

 0

 0

 0

 0

 0

 0

 0

 0

 0

 0

 0

 0

 0

 0

 0

 0

 0

 0

 0

 9.20612514193E-05

 0

 9.20612514193E-05

 0

 0

 0

 0

 0

 0

 0

 0

 0

 0

 0

 0

 4.603062570969E-04

 4.603062570969E-04

 6.13741676129E-05

 0

 0

 .000214809586645

 .000184122502839

 0

 0

 0

 0

 0

 0

 0

 0

 0

 0

 0

 0

 0

 .396538496946

 0

 0

 .170221253874

 .170221253874

 .226317243072

 0

 .226317243072

 0

 4.603062570965E-04

 4.603062570965E-04

 0

 0

 0

 0

 0

 6.13741676129E-05

 .000214809586645

 0

 0

 0

 9.20612514193E-05

 9.20612514193E-05

 0

 0

 0

 0

 0

 0

 0

 0

 0

 0

 0

 0

 0

 0

 0

 0

 0

 0

 0

 0

 0

 0

 0

 0

 0

 0

 0

 0

 0

 0

 0

 0

 0

 0

 0

 0

 0

 0

 0

 0

 0

 0

 0

 0

 0

 0

 0

 0

 0

 0

 0

 0

 0

 0

 0

 0

 0

 0

 0

 0

 0

 .003099395464451

 0

 0

 0

 3.06870838064E-05

 3.06870838064E-05

 3.06870838064E-05

 0

 0

 0

 0

 0

 1.5650412741277E-03

 0

 0

 1.3195446036765E-03

 3.06870838064E-05

 0

 0

 0

 3.06870838064E-05

 0

 .000644428759935

 0

 0

 9.20612514193E-05

 0

 0

 0

 0

 0

 0

 0

 .000490993340903

 0

 0

 0

 0

 0

 3.06870838064E-05

 0

 0

 0

 0

 0

 0

 0

 0

 0

 0

 0

 0

 0

 0

 3.06870838064E-05

 3.06870838064E-05

 0

 0

 0

 0

 1.841225028384E-04

 .000153435419032

 3.06870838064E-05

 0

 0

 0

 0

 0

 0

 3.06870838064E-05

 0

 0

 0

 3.06870838064E-05

 0

 0

 0

 0

 0

 0

 0

 0

 0

 0

 0

 0

 0

 0

 0

 1.4116058550977E-03

 1.4116058550977E-03

 .000153435419032

 3.06870838064E-05

 0

 0

 6.13741676129E-05

 0

 0

 0

 0

 0

 0

 0

 0

 3.06870838064E-05

 0

 0

 0

 0

 .00113542210084

 0

 0

 0

 0

 0

 0

 0

 0

 0

 0

 0

 0

 0

 0

 0

 0

 0

 0

 0

 0

 0

 0

 0

 0

 0

 6.13741676128E-05

 3.06870838064E-05

 3.06870838064E-05

 0

 0

 0

 0

 0

 3.06870838064E-05

 3.06870838064E-05

 0

 0

 0

 0

 0

 0

 0

 0

 0

 0

 0

 0

 0

 0

 0

 0

 0

 0

 0

 3.06870838064E-05

 3.06870838064E-05

 0

 0

 0

 0

 0

 0

 3.06870838064E-05

 0

 0

 0

 0

 0

 0

 0

 0

 0

 0

 0

 0

 0

 0

 0

 0

 0

 0

 0

 .584926504434

 .584926504434

 .584926504434

 .584926504434

 .584926504434

 3.06870838064E-05

 3.06870838064E-05

 0

 0

 0

 0

 0

 0

 0

 0

 0

 0

 0

 0

 0

 0

 0

 0

 0

 0

 0

 0

 0

 0

 0

 0

 0

 0

 0

 3.06870838064E-05

 0

 0

 0

 0

 3.06870838064E-05

 3.06870838064E-05

 0

 0

 0

 0

 0

 0

 0

 0

 0

 0

 0

 0

 0

 0
